# Third Order Upper Bound for the Ground State Energy of the Dilute Bose Gas

**DOI:** 10.1007/s00220-026-05656-8

**Published:** 2026-07-09

**Authors:** Morris Brooks, Jakob Oldenburg, Diane Saint Aubin, Benjamin Schlein

**Affiliations:** Institute for Mathematics, University of Zurich, Winterthurerstrasse 190, 8057 Zurich, Switzerland

## Abstract

We consider a dilute Bose gas in the thermodynamic limit. We prove an upper bound for the ground state energy per unit volume, capturing the expected third order term, as predicted by Wu, Hugenholtz–Pines and Sawada.

## Introduction

We consider a dilute Bose gas, consisting of *N* particles moving in a large, thermodynamic box $\Lambda _t$ with Dirichlet boundary conditions and interacting through a two-body potential *V*. The Hamilton operator of the system is given by

1 $$\begin{aligned} H_{\Lambda _t} = \sum _{i=1}^N -\Delta _i + \sum _{1\le i < j \le N} V (x_i-x_j) \end{aligned}$$

and acts on $L^2_s (\Lambda _t^N)$, the subspace of $L^2 (\Lambda _t^N)$ consisting of all functions that are symmetric with respect to permutations of the *N* particles.

We are going to assume that $V \in L^2 (\mathbb {R}^3)$ is radial, stable (see [35, Section 3.5]), with compact support, with no two-body bound states, and with positive scattering length ${\AA }> 0$ as defined in [30, Appendix C]. In particular, we can consider any non-negative, radial and compactly supported $V\in L^2(\mathbb {R}^3)$. Recall that the scattering length of *V* can be recovered from the solution of the zero-energy scattering equation

2 $$\begin{aligned} \left[ -\Delta + \frac{1}{2} V \right] f = 0 \end{aligned}$$

with the boundary condition f(x)→1 as $|x| \rightarrow \infty$, since $f (x) = 1 - \mathfrak {a} / |x|$, outside the support of *V*. Equivalently,

$$\begin{aligned} 8\pi \mathfrak {a} = \int V(x) f(x) dx \,. \end{aligned}$$

Notice that $f \in L^\infty (\mathbb {R}^3)$, as shown in [27, Theorem 11.7].

We denote by $E (N,\Lambda _t)$ the ground state energy of the gas, ie. the lowest eigenvalue of the operator (1). We consider the thermodynamic limit $N, |\Lambda _t| \rightarrow \infty$, at fixed density $\rho = N/ |\Lambda _t|$. We are interested in the energy per unit volume

$$\begin{aligned} e(\rho ) = \lim _{N, |\Lambda _t| \rightarrow \infty , \rho = N / |\Lambda _t|} \frac{E (N, \Lambda _t)}{| \Lambda _t |} \end{aligned}$$

in the dilute limit $\rho \mathfrak {a}^3 \rightarrow 0$. The following theorem is the main result of our paper.

### Theorem 1

Let $V\in L^2(\mathbb {R}^3)$ be radial, stable and with compact support. Assume that *V* does not admit two-body bound states and let $\mathfrak {a} > 0$ be its scattering length. Then, there is a constant C>0 such that

3 $$\begin{aligned} e(\rho ) \le 4\pi \mathfrak {a}\rho ^2 \left( 1+\frac{128}{15\sqrt{\pi }}\sqrt{\rho \mathfrak {a}^3} + 8\left( \frac{4\pi }{3}-\sqrt{3}\right) \rho \mathfrak {a}^3 \log (\rho \mathfrak {a}^3) + C \rho \mathfrak {a}^3 \right) \end{aligned}$$

for all $\rho \mathfrak {a}^3 > 0$ small enough.

The bound (3) is expected to capture the correct ground state energy per unit volume, up to corrections of order ${\AA }\rho ^2 (\rho \mathfrak {a}^3)$, in the dilute limit $\rho \mathfrak {a}^3 \rightarrow 0$, that are expected to no longer depend universally on $\mathfrak {a}$. The expression on the r.h.s. of (3) has a long history in the physics literature. The first two terms in the asymptotic expansion have been predicted in 1957, by Lee–Huang–Yang [26], based on previous work of Bogoliubov [9]. Two years later, in 1959, Wu [39] and then also Hugenholtz–Pines [25] and Sawada [36] predicted the third logarithmic correction. At the level of rigorous mathematical derivation, the leading order term has been established by Dyson [17] as an upper bound and, 40 years later, by Lieb-Yngvason [32] as a lower bound. For potentials with negative part, the corresponding result was obtained in [38]. An upper bound including the Lee–Huang–Yang term has been proven in [37], for sufficiently regular interactions (previously, an upper bound reaching the Lee–Huang–Yang order, but only recovering the correct constant in the limit of weak coupling, was shown in [18]). More recently, a simpler upper bound has been derived in [5], for a larger class of interactions. The case of hard-sphere potential, however, remains open (for hard-spheres, the currently best available upper bound on the ground state energy per unit volume has been shown in [3], with an error of the Lee–Huang–Yang order). A lower bound resolving the Lee–Huang–Yang contribution has been first shown in [19] and, for more general interactions including hard spheres, in [20]. Recently, a new proof of the lower bound, applying also to the free energy at low positive temperature, has been obtained in [21] (a matching low temperature upper bound has been derived in [22]). The result of [21] has been extended to potentials including hard spheres in [23].

Theorem 1 establishes an upper bound on the ground state energy per unit volume of a dilute Bose gas, interacting through $V \in L^2 (\mathbb {R}^3)$, consistent with Wu’s prediction. A complete proof of the validity of Wu’s formula in the thermodynamic limit requires a matching lower bound, which remains a challenging open problem. Remark, however, that a rigorous derivation of Wu’s correction is available in the Gross-Pitaevskii regime, where *N* particles are confined in the unit torus and interact through a potential with scattering length of the order 1/*N*. In this limit, the ground state energy and the low-energy excitation spectrum have been resolved to the Lee–Huang–Yang order in [6, 7], through a rigorous version of Bogoliubov theory (leading order estimates and a proof of Bose–Einstein condensation have been previously obtained in [28, 29, 31]). Recently, extending the approach of [6, 7], Wu’s logarithmic correction to the ground state energy has been resolved in [10]; we will use ideas from [10] also in the current paper, in particular in Sect. 2.2 to define the kernel of the cubic transformation. Related results in the Gross-Pitaevskii regime (and beyond) have been derived in [1, 4, 8, 11–16, 24, 33, 34].

The proof of Theorem 1 is based on a well-known localization technique; given a grand canonical trial state $\psi _{L}$ on a periodic box of size $L = \rho ^{-\gamma }$, with approximately $\rho L^3$ particles and with approximately the correct energy per unit volume, we can construct a grand canonical trial state on the thermodynamic box $\Lambda _t$ with approximately the correct energy, first extending $\psi _{L}$ to a trial state $\psi _{L}^\text {dir}$ on a Dirichlet box of size slightly larger than *L* and then combining several copies of $\psi _{L}^\text {dir}$ to cover the whole volume of $\Lambda _t$ (to avoid interactions among particles in different boxes, they must be separated by small corridors, broader than the support of *V*). At the end, (3) follows by the equivalence of ensembles.

The main part of the paper is devoted to the construction of the grand canonical trial state on the periodic box $\Lambda _L$, of size $L = \rho ^{-\gamma }$ for appropriate γ>0 (to control the localization error produced by imposing Dirichlet boundary conditions, we will need $\gamma \ge 3/2$). In fact, we find it more convenient to rescale lengths, and work on the unit torus $\Lambda \equiv \Lambda _1 \simeq [-1/2;1/2]^3$. Denoting by $N = \rho L^3 = \rho ^{1-3\gamma }$ the expected number of particles in $\Lambda _L$, we find $\rho = N^{-1/(3\gamma -1)}$ and $L = N^{\gamma / (3\gamma -1)} = N^{1-\kappa }$, where we introduced the parameter $\kappa = (2\gamma -1)/(3\gamma -1)$. For $\kappa \in [0;2/3)$, we consider the Hamilton operator

4 $$\begin{aligned} \mathcal {H}_N = \sum _{p\in \Lambda ^*} p^2 a_p^* a_p + \frac{N^\kappa }{2N} \sum _{p,q,r\in \Lambda ^*} \hat{V}(r/N^{1-\kappa }) a_{p+r}^* a_{q-r}^* a_q a_p \end{aligned}$$

acting on the bosonic Fock space $\mathcal {F}(\Lambda ) = \bigoplus _{n\ge 0}L^2_s(\Lambda ^n)$. Here, $\Lambda ^*= 2\pi \mathbb {Z}^3$ is the dual lattice and, for $p \in \Lambda ^*$, $a_p^*, a_p$ are the usual creation and annihilation operators, satisfying canonical commutation relations.

### Theorem 2

Under the same assumptions on *V* as in Theorem 1, we have that for any $\kappa \in [1/2,2/3)$ and *N* large enough there exists $\Psi _N\in \mathcal {F}(\Lambda )$, $\Vert \Psi _N \Vert =1$ such that

5 $$\begin{aligned} \langle \Psi _N, \mathcal {N}\Psi _N \rangle \ge N, \quad \langle \Psi _N, \mathcal {N}^2 \Psi _N \rangle \le CN^2 \end{aligned}$$

and

6 $$\begin{aligned} \begin{aligned} \langle \Psi _N, \mathcal {H}_N \Psi _N \rangle \le \;&4\pi \mathfrak {a} N^{1+\kappa } +\frac{512\sqrt{\pi }}{15}\mathfrak {a}^{5/2}N^{5\kappa /2}\\&+ 32\pi \left( \frac{4\pi }{3}-\sqrt{3}\right) \mathfrak {a}^4 N^{4\kappa -1} \log (N^{3\kappa -2}) + C N^{4\kappa -1}. \end{aligned} \end{aligned}$$

*Remark.* The restriction to $\kappa \ge 1/2$ makes sure that finite size errors of order $N^{2\kappa }$ are negligible.

With Theorem 2, we can prove our main result. Throughout this manuscript we will use the symbols *c* and *C* for constants that may vary from line to line.

### Proof of Theorem 1

We first discuss the case V≥0. To translate the upper bound (6) into a bound for the ground state energy per unit volume in the thermodynamic limit we use [5, Prop. 1.2], whose proof relies on [35], requiring, in particular, stability of the potential. For V≥0, proposition [5, Prop. 1.2] states that, if R<ℓ<L, with *R* the radius of the support of the potential *V*, and if $\psi _L \in \mathcal {F}(\Lambda _L)$ is normalized and such that

7 $$\begin{aligned} \langle \psi _L, \mathcal {N}\psi _L \rangle \ge \rho (1+c \rho ) (L+2\ell +R)^3, \qquad \langle \psi _L, \mathcal {N}^2 \psi _L \rangle \le C \rho ^2 (L+2\ell +R)^6 \end{aligned}$$

then we have

8 $$\begin{aligned} e(\rho ) \le \frac{\langle \psi _L, \mathcal {H}\psi _L \rangle }{L^3} + \frac{C}{L^4 \ell } \langle \psi _L, \mathcal {N}\psi _L \rangle \end{aligned}$$

with the Hamilton operator

$$\begin{aligned} \mathcal {H}= \sum _{p \in \Lambda _L^*} p^2 a_p^* a_p + \frac{1}{2L^3} \sum _{p,q,r \in \Lambda ^*_L} \hat{V} (r) a_{p+r}^* a_{q-r}^* a_q a_p \end{aligned}$$

acting on $\mathcal {F}(\Lambda _L)$ (where $\Lambda ^*_L = 2\pi \mathbb {Z}^3 / L$).

Notice that the state $\psi _L \in \mathcal {F}(\Lambda _L)$, whose energy appears on the r.h.s. of (8) and provides an upper bound to e(ρ), must have, effectively, a slightly larger density than ρ (to compensate the fact that, to construct the thermodynamic trial state, we first need to increase a bit the size of the box $\Lambda _L$ to impose Dirichlet boundary conditions and to make sure that particles in different boxes do not interact).

Given a density ρ>0, we thus choose $\widetilde{\rho } > \rho$ (to be specified later on) and we apply Theorem 2. Rescaling lengths, we use the trial state $\Psi _N \in \mathcal {F}(\Lambda )$ to define

$$\begin{aligned} \psi ^{(m)}_L (x_1,\dots ,x_m) := L^{-3m/2} \Psi ^{(m)}_N (L^{-1}x_1,\dots ,L^{-1}x_m) \,. \end{aligned}$$

We obtain, for any γ>1 (reflecting the condition $\kappa = (2\gamma -1)/(3\gamma -1) \in (1/2; 2/3)$), a normalized $\psi _L = \{ \psi ^{(m)}_L \}_{m \in \mathbb {N}} \in \mathcal {F}(\Lambda _L)$ on the periodic box of size $L = \widetilde{\rho }^{-\gamma }$ (so that $N:=\widetilde{\rho } L^3= \widetilde{\rho }^{1-3\gamma }$), with

$$\begin{aligned} \langle \psi _L, \mathcal {N}\psi _L \rangle \ge \widetilde{\rho } L^3, \qquad \langle \psi _L, \mathcal {N}^2 \psi _L \rangle \le C \widetilde{\rho }^2 L^6 \end{aligned}$$

and (since $\mathcal {H}_N$ is unitarily equivalent to $L^2 \mathcal {H}$) with

$$\begin{aligned} \frac{\langle \psi _L, \mathcal {H}\psi _L \rangle }{L^3} \le 4 \pi \mathfrak {a} \widetilde{\rho }^2 \left( 1 + \frac{128}{15\sqrt{\pi }} \sqrt{\widetilde{\rho } \mathfrak {a}^3} + 8 \left( \frac{4\pi }{3} - \sqrt{3} \right) \widetilde{\rho } \mathfrak {a}^3 \log (\widetilde{\rho } \mathfrak {a}^3) + C \widetilde{\rho } \mathfrak {a}^3 \right) . \end{aligned}$$

To make sure that (7) holds true, we set $\ell = L^\alpha$, for 0<α<1, and we apply the implicit function theorem to find $\widetilde{\rho } > \rho$ such that

$$\begin{aligned} \widetilde{\rho } = \rho (1 + c \rho ) (1 + 2 \widetilde{\rho }^{\gamma (1-\alpha )} + R \widetilde{\rho }^{\gamma })^3. \end{aligned}$$

From (8), we conclude therefore that

$$\begin{aligned} e (\rho ) \!\le \! 4 \pi \mathfrak {a} \widetilde{\rho }^2 \left( 1 \!+\! \frac{128}{15\sqrt{\pi }} \sqrt{\widetilde{\rho } \mathfrak {a}^3} \!+\! 8 \left( \frac{4\pi }{3} \!-\! \sqrt{3} \right) \widetilde{\rho } \mathfrak {a}^3 \log (\widetilde{\rho } \mathfrak {a}^3) + C \widetilde{\rho } \mathfrak {a}^3 \right) + C \widetilde{\rho }^{1+\gamma (1+\alpha )}. \end{aligned}$$

Since $\widetilde{\rho }^2 \le \rho ^2 (1 + C \rho + C \rho ^{\gamma (1-\alpha )})$, we obtain

9 $$\begin{aligned} \begin{aligned} e (\rho ) \le \;&4 \pi \mathfrak {a} \rho ^2 \left( 1 + \frac{128}{15\sqrt{\pi }} \sqrt{\rho \mathfrak {a}^3} + 8 \left( \frac{4\pi }{3} - \sqrt{3} \right) \rho \mathfrak {a}^3 \log (\rho \mathfrak {a}^3) \right) \\&+ C (\rho ^3 + \rho ^{2+ \gamma (1-\alpha )} + \rho ^{1+ \gamma (1+\alpha )}). \end{aligned} \end{aligned}$$

Choosing $\alpha = 1/(2\gamma )$, we find the error $\rho ^{3/2+\gamma }$. Choosing $\gamma \ge 3/2$ (which corresponds to $\kappa \ge 4/7$, using the language of Theorem 2), we arrive at the desired bound, concluding the proof of theorem, for V≥0.

Without the condition V≥0 (but still assuming stability), we can proceed similarly. In this case, however, we generate an additional error term on the r.h.s. of (8), arising from the periodization of $V_-$, the negative part of *V*. Proceeding as in [5], this error term can be bounded by

$$\begin{aligned} \frac{\ell }{L^4} \langle \psi _L, d\Gamma (V_-) \psi _L \rangle \end{aligned}$$

with $d\Gamma (V_-)$ denoting the second quantization of $V_-$. From our analysis, we can clearly estimate $\langle \psi _L, d\Gamma (V_-) \psi _L \rangle \le C \tilde{\rho }^2 L^3$; hence, the additional error can be bounded by the term $\rho ^{2+\gamma (1-\alpha )}$, already appearing on the r.h.s. of (9). Therefore, the proof can be concluded as above. □

The rest of the paper is devoted to the proof of Theorem 2. Let us briefly explain, on a heuristic level, the main ideas involved in the proof of (3). We start from Bogoliubov theory [9]. First, we factor out the Bose–Einstein condensate, conjugating the Hamiltonian (4) with a Weyl operator generating a coherent state with, in average, $N_0$ particles with momentum p=0. In the second step, we conjugate the resulting excitation Hamiltonian with a Bogoliubov transformation $e^{B^* - B}$, with

10 $$\begin{aligned} B = \frac{1}{2} \sum _{|p| \ge N^{\kappa /2-\beta /4}} \mu _p a_p a_{-p} \end{aligned}$$

where $\beta =2-3\kappa$ and with coefficientssatisfying11We note that the argument  of the logarithm is positive for all momenta $p \in \Lambda ^*$, if *N* is large enough, as  and thus  for ξ small enough and  by our assumptions on *V*. This leads us to a new, unitarily equivalent, Hamiltonian

12 $$\begin{aligned} \widetilde{\mathcal {H}}_N \simeq C_N + \mathcal {K}+ \mathcal {C}_N + \mathcal {C}_N^* + \mathcal {V}_N \end{aligned}$$

where the constant $C_N$ gives the correct ground state energy to leading order (and already contains some terms contributing to the Lee–Huang–Yang correction),

$$\begin{aligned} \mathcal {K}= \sum _{p\in \Lambda ^*} p^2 a_p^* a_p,\end{aligned}$$

is the kinetic energy operator,

$$\begin{aligned} \mathcal {C}^*_N = \frac{1}{\sqrt{N}} \sum _{p,r \in \Lambda ^*} \hat{V} (r/N^{1-\kappa }) \sigma _p a_{p+r}^* a_{-r}^* a_p^* \end{aligned}$$

with $$\sigma _p = \sinh \mu _p$$ and

$$\begin{aligned} \mathcal {V}_N = \frac{1}{2N} \sum _{r,p,q \in \Lambda ^*} \hat{V} (r/N^{1-\kappa }) a_{p+r}^* a_q^* a_{q+r} a_p \,. \end{aligned}$$

The two Bogoliubov transformations that have been used, for example, in [6, 7], are combined here into (10). For large momenta $|p| \gg N^{\kappa /2}$,  approximates the solution of the zero-energy scattering equation (2) and the action of (10) helps renormalizing the interaction potential. For $|p| \lesssim N^{\kappa /2}$, on the other hand, the Bogoliubov transformation diagonalizes the quadratic terms in the excitation Hamiltonian (the identity (11) makes sure that $e^{B^* - B}$ diagonalizes exactly a quadratic Hamiltonian with potential *Vf*). In (10), we restrict the sum to momenta $|p| \ge N^{\kappa /2-\beta /4}$, with the exponent $\beta = 2-3\kappa$; contributions arising from $|p| < N^{\kappa /2-\beta /4}$ do not change the energy, up to order $N^{4\kappa -1}$ (to resolve the Lee–Huang–Yang energy, it is enough to consider $|p| \gtrsim N^{\kappa /2}$).

Wu [39] proposed to use second order perturbation theory to obtain a better approximation for the ground state energy, essentially treating $\mathcal {K}$ as the unperturbed Hamiltonian and $\mathcal {C}_N + \mathcal {C}_N^* +\mathcal {V}_N$ as the perturbations (first order perturbation theory gives a vanishing contribution, because the ground state of $\mathcal {K}$ is the vacuum and the vacuum expectation of $\mathcal {C}_N, \mathcal {C}_N^*, \mathcal {V}_N$ is zero). This remark suggests that a good trial state for the renormalized Hamiltonian $\widetilde{\mathcal {H}}_N$ could have the form13where we used the renormalized potential to make sure that the energy of ξ only depends on the scattering length and where we restricted the sum to $|r| \ge N^{\kappa /2}$ and $|v| \ge N^{\kappa /2-\beta /4}$, since contributions from smaller momenta do not affect the energy, to order $N^{4\kappa -1}$. It turns out that, with this choice of ξ,

14 $$\begin{aligned} \langle \xi , (\mathcal {K}+ \mathcal {C}_N + \mathcal {C}_N^* + \mathcal {V}_N) \xi \rangle = \langle \Omega , (A \mathcal {C}_N^* + \mathcal {C}_N A^* ) \Omega \rangle + \langle \Omega , A (\mathcal {K}+ \mathcal {V}_N ) A^* \Omega \rangle \end{aligned}$$

leads, together with the constant term $C_N$ in (12), exactly to the correct expression for the ground state energy, including the third order term in (3). The problem with this approach is the fact that ξ is not normalized. Since

$$\begin{aligned} \Vert \xi \Vert ^2 = 1 + \Vert A^* \Omega \Vert ^2 \simeq C N^{3\kappa -1} \end{aligned}$$

the trial state $\xi / \Vert \xi \Vert$ only provides a good upper bound for the ground state energy, if κ=0. To avoid problems with the normalization, we will consider a trial state defined through a unitary map. We define, formally, $\xi = e^{A^* - A} \Omega$, with *A* as in (13). Since

$$\begin{aligned} \langle \Omega , [ \mathcal {C}_N + \mathcal {C}_N^*, A^* - A ] \Omega \rangle = \langle \Omega , ( \mathcal {C}_N A^* + A \mathcal {C}_N^* ) \Omega \rangle \end{aligned}$$

and

$$\begin{aligned} \langle \Omega , [ [ \mathcal {K}+ \mathcal {V}_N, A^* - A], A^* - A ] \Omega \rangle = 2 \langle \Omega , A (\mathcal {K}+ \mathcal {V}_N) A^* \Omega \rangle \end{aligned}$$

the trial state ξ does indeed generate the contributions (14), leading to Wu’s correction. To obtain a rigorous upper bound, though, we would have to show that $e^{A^* - A}$ is well-defined as a unitary operator and we would have to show convergence of its Duhamel expansion.

On the one hand, we observe that the vacuum expectation of multiple commutators like $[ A, [ A^*, [ A, \dots [ A^*, O] \dots ]$ gets smaller, with increasing number of commutators. In fact, for every two additional commutators, we have four more momenta to sum up. When considering the vacuum expectation, however, we also have three more delta functions. Effectively, every new pair of commutators with $A,A^*$ produces therefore only one additional momentum sum, typically of the order $\Vert \sigma \Vert _2^2 \simeq N^{3\kappa /2}$. Since each factor $A,A^*$ carries a small constant $N^{-1/2}$, every two new commutators generate an additional small factor $N^{3\kappa /2 - 1} \ll 1$.

On the other hand, to control error terms arising in the Duhamel expansion, it is very useful to introduce a cutoff on the local number of particles. For $x \in \Lambda$ and ℓ>0, we consider the operator

$$\begin{aligned} \mathcal {N}_\ell (x) = \int _{|x-y| \le \ell } dy \, a_y^* a_y \end{aligned}$$

measuring the number of particles in a ball of radius ℓ around *x*. Since we used an infrared cutoff in momentum space (in fact, we will work with smooth cutoffs), the kernel $\check{\sigma } (x)$ exhibits fast decay, for $|x| \gg \ell _\sigma := N^{-\kappa /2+\beta /4}$. Similarly, expanding the cubic operator *A* defined in (13) in position space as

$$\begin{aligned} A = \int dx_1 dx_2 dx_3 \, \check{A} (x_1-x_2,x_1-x_3) a_{x_1} a_{x_2} a_{x_3} \end{aligned}$$

we find that the kernel $\check{A} (y,x)$ is very small for $|x| \gg \ell _\sigma$ or for $|y| \gg \ell _\eta := N^{-\kappa /2}$. In fact, $\check{\sigma } (x)$ and $\check{A} (y,x)$ also decay for $|x| < \ell _\sigma$, with rate proportional to $|x|^{-5/2}$. This implies that their $L^2$-norm is essentially supported in the region $|x| \le \ell _B = N^{-\kappa /2 + \beta /12} \ll \ell _\sigma$, in the sense that, if we introduce a lattice $\Lambda _B = \ell _B \mathbb {Z}^3$ and, for every $u \in \Lambda _B \cap \Lambda$, we define $\sigma _u = \sigma \mathbb {1}_{B_u}$, with $B_u$ the box with side length $\ell _B$ centered at *u*, then

15 $$\begin{aligned} \sum _{u \in \Lambda _B} \Vert \check{\sigma }_u \Vert _2 \le C N^{3\kappa /4 + \epsilon } \end{aligned}$$

for any ϵ>0. Up to the additional factor $N^\epsilon$, this is comparable with $\Vert \check{\sigma } \Vert _2$. To take advantage from the decay of the kernels $\check{\sigma }$, $\check{A}$, we will therefore modify the trial state, defining $\xi = e^{A^* \Theta - \Theta A} \Omega$, where we introduced an operator Θ, making sure that

16 $$\begin{aligned} \Theta \mathcal {N}_{\ell _B} (x) \Theta \le C N^\epsilon \end{aligned}$$

for any ϵ>0 and every $x \in \Lambda$, cutting off the local number of particles (more precisely, the local number of cubic excitations) in balls of radius $\ell _B = N^{-\kappa /2 + \beta /12}$. Since the expected (by Duhamel expansion) average number of particles generated by the cubic transformation in a ball of radius $\ell _B$ is of the order

$$\begin{aligned} \ell ^3_B \Vert A^* \Omega \Vert ^2 \simeq \ell ^3_B N^{3\kappa -1} \simeq N^{-\beta /4} \ll 1, \end{aligned}$$

the cutoff does not substantially change the energy of the trial state (but it is nevertheless important to control error terms).

To explain how we use the cutoff Θ, let us try to estimate the number of particles generated by the unitary operator $e^{\Theta \widetilde{A}^* - \widetilde{A} \Theta }$, where we replaced *A* by its simplified version

$$\begin{aligned} \widetilde{A} = \frac{1}{\sqrt{N}} \sum _{|p| \ge N^{\kappa /2}, |q| \ge N^{\kappa /2 - \beta /4}} \eta _p \sigma _q a_{p+q} a_{-p} a_{-q} \end{aligned}$$

with . We have

17 $$\begin{aligned} \langle e^{\Theta \widetilde{A}^* - \widetilde{A}\Theta } \Omega , \mathcal {N}e^{\Theta \widetilde{A}^* - \widetilde{A}\Theta } \Omega \rangle = 2 \text {Re } \int _0^1 ds \langle e^{s(\Theta \widetilde{A}^* - \widetilde{A} \Theta )} \Omega , [\mathcal {N}, \widetilde{A} ] \Theta e^{s(\Theta \widetilde{A}^* - \widetilde{A} \Theta )} \Omega \rangle .\nonumber \\ \end{aligned}$$

Setting $\xi _s = e^{s(\Theta \widetilde{A}^* -\widetilde{A} \Theta )} \Omega$ and switching to position space, we find

$$\begin{aligned} \begin{aligned} \big | \langle \xi _s, [ \mathcal {N}, \widetilde{A} ] \Theta \xi _s \rangle \big |&\le 3 \int dx dy \, | \check{\eta } (x-y)| |\check{\sigma } (x-z)| \Vert a_x a_y a_z \Theta \xi _s \Vert \Vert \xi _s \Vert \\  &\le 3 \sum _{u \in \Lambda _B} \int dx dy \, |\check{\eta } (x-y)| |\check{\sigma }_u (x-z)| \Vert a_x a_y a_z \Theta \xi _s \Vert \Vert \xi _s \Vert . \end{aligned} \end{aligned}$$

By Cauchy–Schwarz and using that $\check{\sigma }_u (x-z)$ is supported on $|x-z-u| \le \ell _B$ and $\check{\eta } (x-y)$ is essentially supported on $|x-y| \le \ell _\eta$, we conclude that

18 $$\begin{aligned} \big | \langle \xi _s, [ \mathcal {N}, \widetilde{A} ] \Theta \xi _s \rangle \big |&\le 3\Vert \check{\eta } \Vert _2 \sum _{u \in \Lambda _B} \Vert \check{\sigma }_u \Vert _2 \Big ( \int dx \, \Vert \mathcal {N}^{1/2}_{\ell _B} (x-u) \mathcal {N}^{1/2}_{\ell _\eta } (x) a_x \Theta \xi _s \Vert ^2 \Big )^{1/2} \Vert \xi _s \Vert +\mathcal {E}. \nonumber \\ \end{aligned}$$

Here, $\mathcal {E}$ is a very small error coming from the rapid decay. With $\Vert \eta \Vert _2 \le C N^{3\kappa /4}$ and using (15) and (16) (after passing Θ through $a_x$), we obtain

$$\begin{aligned} \big | \langle \xi _s, [ \mathcal {N}, \widetilde{A} ] \Theta \xi _s \rangle \big | \le C N^{3\kappa /2-1/2+ \epsilon } \Vert \mathcal {N}^{1/2} \xi _s \Vert \Vert \xi _s \Vert \le C N^{3\kappa -1+\epsilon } \Vert \xi _s \Vert ^2 + C \Vert \mathcal {N}^{1/2} \xi _s \Vert ^2. \end{aligned}$$

Inserting in (17) and applying Gronwall’s Lemma, we arrive at

$$\begin{aligned} \langle e^{\widetilde{A}^* \Theta - \Theta \widetilde{A}} \Omega , \mathcal {N}e^{\widetilde{A}^* \Theta - \Theta \widetilde{A}} \Omega \rangle \le C N^{3\kappa -1+2\epsilon } \end{aligned}$$

which is almost the optimal result (one can remove the additional $N^\epsilon$, expanding (17) to the next order; this will be shown for the full cubic operator *A* in Sect. 3.2). Deriving such an estimate bounding the r.h.s. of (18) with the global, rather than the local, number of particles operator would have been impossible. Also, localizing σ on boxes of size $\ell _\sigma$, rather than $\ell _B \ll \ell _\sigma$, would have led to a worse, non-optimal, dependence on *N*.

Let us briefly compare the cutoff to the one used in [5] for obtaining the upper bound for the Lee–Huang–Yang order. In [5], a cutoff in momentum space is used which allows to define a non-unitary exponential of a cubic operator acting on the vacuum used to implement cubic correlations on top of the condensate and pair correlations. The crucial role of that cutoff is that it ensures that all relevant triplets of momenta adding up to 0 are generated by the same copy of $A^*$ in states of the form ${A^*}^n\Omega$ and therefore have the right correlation structure. Note that the number of momenta that cannot be occupied due to this restriction scales roughly quadratically in the number of cubic excitations, and thus, for the number of triplets that is necessary for finding the Lee–Huang–Yang term, it becomes comparable to the total number of relevant momenta when κ=7/12. This is precisely the threshold until which the trial state in the extension [2] of [5] works. The third order correction requires new ideas due to the higher precision that is required. In particular, by plugging in the estimated number of triplets necessary here, the same heuristics explains why the critical threshold of κ=4/7 cannot be reached with this cutoff. The cutoff introduced here resolves this issue by imposing a restriction in position space rather than in momentum space. As a result, it works for κ arbitrarily close to 2/3.

The plan of the paper is as follows. In the next section, we define our trial state $\psi _N = W_{N_0} e^{B^* - B} e^{A^* \Theta - \Theta A} \Omega$, where $W_{N_0}$ is the Weyl operator, generating the condensate in the zero momentum state, $e^{B^* - B}$ is the Bogoliubov transformation and $e^{A^* \Theta - \Theta A}$ is the cubic transformation. To this end, in Sects. 2.1, 2.2 we define the coefficients of *B* and *A* and we study their properties, in momentum and in position space. In Sect. 2.3, we introduce the cutoff Θ for the local number of particles operator. In Sect. 3, we will then compute the energy of our trial state; in Sect. 3.1 we first compute the action of the Bogoliubov transformation on the Hamilton operator. This reduces the problem to the computation of certain expectations, in the state $\xi = e^{A^* \Theta - \Theta A} \Omega$, which will be discussed in Sects . 3.2, 3.3. This will allow us to complete the proof of Theorem 2 in Sect. 3.4.

## The Trial State

This section is devoted to the construction of the trial state $\psi _N \in \mathcal {F}(\Lambda )$, which will be later shown to satisfy (5) and (6).

We will consider a trial state of the form

19 $$\begin{aligned} \psi _N = W_{N_0} e^{B-B^*} e^{A^* \Theta - \Theta A} \Omega . \end{aligned}$$

Here, $\Omega \in \mathcal {F}(\Lambda )$ is the vacuum vector. Moreover,

20 $$\begin{aligned} W_{N_0} = \exp (\sqrt{N_0} a_0^* - \text {h.c.} ) \end{aligned}$$

is a Weyl operator, satisfying

21 $$\begin{aligned} W_{N_0}^* a_p W_{N_0} = a_p + \delta _{p,0} \sqrt{N_0} \end{aligned}$$

for all $p \in \Lambda ^* = 2\pi \mathbb {Z}^3$ and generating a coherent state with (in average) $N_0$ particles with momentum p=0 (the Bose–Einstein condensate).

On the other hand, $e^{B-B^*}$ and $e^{A^* \Theta - \Theta A}$ are unitary maps, with

22 $$\begin{aligned} B = \frac{1}{2} \sum _{p \in \Lambda ^* \backslash \{ 0 \}} \mu _p a_p a_{-p} \end{aligned}$$

quadratic and

23 $$\begin{aligned} A = \sum _{p,q \in \Lambda ^*} A_{p,q} \, a_{p+q} a_{-p} a_{-q} \end{aligned}$$

cubic in creation and annihilation operators. The operator Θ, appearing in the cubic transformation, is a cutoff, controlling the local particle density. In the next subsections, we are going to define the kernels $\mu _p$, $A_{p,q}$ and the cutoff Θ precisely and we are going to study their most important properties.

### Coefficients of the Bogoliubov transformation

We use the solution *f* of the zero-energy scattering equation (2) to define the coefficients24and

25 $$\begin{aligned} \mu _{\infty ,p} = -\frac{1}{4} \log (1-4\eta _{\infty ,p}) \end{aligned}$$

for $p\in \Lambda ^*_+ = 2\pi \mathbb {Z}^3\backslash \{0\}$. We also define $\eta _{\infty ,0}= \mu _{\infty ,0}=0$. Recall that analogously to the definition after (10), the argument of the logarithm is positive as $\eta _p \le 0$ for $|p |\lesssim N ^{1-\kappa }$ and $|\eta _p |\le C N ^{3\kappa -2} \ll 1$ for $|p |\gtrsim N ^{1-\kappa }$. Notice thatHence, a Bogoliubov transformation with coefficients $\mu _{\infty , p}$ would exactly diagonalize a quadratic Hamiltonian with interaction *Vf* instead of *V*. To describe the action of such a Bogoliubov transformation, we define the coefficients $\sigma _{\infty ,p} = \sinh \mu _{\infty ,p}$ and $\gamma _{\infty ,p} = \cosh \mu _{\infty ,p}$. We have26Some useful properties of the coefficients $\eta _{\infty ,p}$, $\sigma _{\infty ,p}$ and $\gamma _{\infty ,p}$ are collected in the following lemma.

#### Lemma 3

The coefficients $\eta _{\infty ,q}$ satisfy the equation

27 $$\begin{aligned} p^2 \eta _{\infty ,p} +\frac{N^\kappa }{2} \hat{V}(p/N^{1-\kappa }) +\frac{N^\kappa }{2N} \sum _{q\in \Lambda _+^*} \hat{V}((p-q)/N^{1-\kappa })\eta _{\infty ,q} = O(N^{2\kappa -1}) \end{aligned}$$

uniformly in $p \in \Lambda ^*_+$. Furthermore,

28 $$\begin{aligned} \sum _{p\in \Lambda _+^*} N^\kappa \hat{V}(p/N^{1-\kappa })\eta _{\infty ,p} = (8 \pi \mathfrak {a}- \hat{V}(0)) N^{1+\kappa } + O(N^{2\kappa }). \end{aligned}$$

The coefficients $\gamma _{\infty ,p}$ and $\sigma _{\infty ,p}$ satisfy29and therefore, for s∈(3/2,6),

30 $$\begin{aligned} \sum _{p\in \Lambda _+^*} |\gamma _{\infty ,p}-1 |^s \le \sum _{p\in \Lambda _+^*} |\sigma _{\infty ,p} |^s \le C N^{3\kappa /2} \end{aligned}$$

as well as

31 $$\begin{aligned} \sum _{p\in \Lambda _+^*} |\gamma _{\infty ,p}-1 | \le \sum _{p\in \Lambda _+^*} |\sigma _{\infty ,p} | \le C N. \end{aligned}$$

We also have

32 $$\begin{aligned} |\gamma _{\infty ,p}\sigma _{\infty ,p} - \eta _{\infty ,p} | \le C \min \Big ( \frac{N^{\kappa }}{|p |^2}, \frac{N^{2\kappa }}{|p |^4} \Big ) \end{aligned}$$

and therefore

33 $$\begin{aligned} \sum _{p\in \Lambda _+^*} |\gamma _{\infty ,p}\sigma _{\infty ,p} - \eta _{\infty ,p} | \le C N^{3\kappa /2}. \end{aligned}$$

Before proving Lemma 3, we establish some preliminary bounds. In the following $\mathcal {F}[g] = \{ \mathcal {F}[g] \}_{p \in \Lambda ^*}$ denotes the Fourier coefficients of $g: \Lambda \rightarrow \mathbb {C}$. Note that, for a function $g:\mathbb {R}^3\longrightarrow \mathbb {C}$ supported in $[-1/2,1/2]^3$, such as $(Vf)(N^{1-\kappa }\cdot )$ for large enough *N*, $\mathcal {F}[g] (p) = \hat{g} (p)$ for all $p \in \Lambda ^*$ (here, $\hat{g}: \mathbb {R}^3 \rightarrow \mathbb {C}$ denotes the Fourier transform of *g* on the full space). By a slight abuse of notation we denote *g* and its restriction to Λ (respectively its periodic extension) in the same way, i.e. we simply write $\mathcal {F}[g]$ instead of $\mathcal {F}[g|_{\Lambda }]$. Moreover, for $k = (k_1, k_2, k_3) \in \mathbb {N}^3$, we use the standard notation $|k| = k_1 + k_2 + k_3$ and $x^k = x_1^{k_1} x_2^{k_2} x_3^{k_3}$.

#### Lemma 4

For $m \in \mathbb {N}$, let34Then, for every $m \in \mathbb {N}$ there is a constant $C_m > 0$ such that

35 $$\begin{aligned} \Vert F_m \Vert _\infty \le C_m \end{aligned}$$

and

36 $$\begin{aligned} \begin{aligned} \sum _{p\in \Lambda ^*} F_{m}^2((p+\zeta )/N^{1-\kappa }) \le C_m N^{3-3\kappa } \end{aligned} \end{aligned}$$

for all $N \in \mathbb {N}$ and all $\zeta \in \mathbb {R}^3$.

#### Proof

The bound (35) follows immediately from . Also, using this inequality we havesince *Vf* has compact support and $\Vert Vf\Vert _1\le C$. This shows (36). □

We are now ready to show Lemma 3.

#### Proof of Lemma 3

We define w=1-f and note that by the scattering equation (2) we have $w=(-\Delta )^{-1} Vf / 2$, or equivalently . For p≠0, we obtain37where we used in particular the definition (24) of $\eta _{\infty ,p}$ and where the error $O (N^{2\kappa -1})$ arises from the contribution associated with q=0, since $\Vert Vf\Vert _1+\Vert V\Vert _1 \le C$. Henceand, since the *s*-derivative evaluated at s=0 is odd in ζ,38Using the function $F_2$, defined in (34), we can boundTo bound the sum over $|q+t \zeta | > N^{1-\kappa }$, we used Cauchy–Schwarz and (36). From (38), we obtain (27), uniformly in $p \in \Lambda ^*_+$.

To prove (28) we observe that, starting from the second line, (37) also holds for p=0. This implies thatWith , we obtain (28).

The bounds (29) to (33) are easy to check using the explicit form in (26). □

Let $\beta = 2-3\kappa > 0$. Noticing with (29) that

39 $$\begin{aligned} N^\kappa \sum _{|p| < N^\alpha } \sigma _{\infty ,p}^2 \le C N^{3\kappa /2+2\alpha } \le CN^{4\kappa -1} \end{aligned}$$

if $\alpha \le \kappa /2 - \beta /4$, we conclude that excitations with momentum below $N^{\kappa /2-\beta /4}$ are negligible, to resolve the energy to the precision we are aiming for (the l.h.s. of (39) arises from the quadratic term $N^\kappa \sum _p \hat{V} (p/N^{1-\kappa }) a_p^* a_p$, after conjugation with the Bogoliubov transformation; also the contribution of the other quadratic terms in the excitation Hamiltonian is negligible, for $|p| < N^{\kappa /2 - \beta /4}$). Thus, we can impose an infrared cutoff on the coefficients $\mu _p$ of the Bogoliubov transformation defined in (22). In order to guarantee decay in position space, it is important to use a smooth cutoff. We choose $\chi _l \in C^{\infty }(\mathbb {R})$ with $0\le \chi _l \le 1$, $\chi _l(x)=0$ if x≤1, $\chi _l(x)=1$ if x≥2, and we set $\chi (p) = \chi _l (|p |/N^{\kappa /2-\beta /4})$. For $p \in \Lambda ^*$, we define

40 $$\begin{aligned} \mu _p :=\mu _{\infty ,p} \chi (p) \end{aligned}$$

with $\mu _{\infty ,p}$ as in (25). With these coefficients we set

$$\begin{aligned} B = \frac{1}{2} \sum _{p \in \Lambda ^*} \mu _p a_p a_{-p} \,. \end{aligned}$$

The trial state (19) is defined through the Bogoliubov transformation $e^{B-B^*}$, whose action on creation and annihilation operators is determined by

41 $$\begin{aligned} e^{B-B^*} a_p e^{B^*-B} = \gamma _p a_p + \sigma _p a_{-p}^*, \qquad e^{B-B^*} a^*_p e^{B^*-B} = \gamma _p a^*_p + \sigma _p a_{-p} \end{aligned}$$

where, similarly as in (26), we introduced the notation $\sigma _p = \sinh \mu _p$, $\gamma _p = \cosh \mu _p$, for all $p \in \Lambda ^*$. In position space, we setWe will also make use of continuous versions of the coefficients $\mu _p, \sigma _p, \gamma _p$. Analogously to (24), (25), we define therefore, for every $p \in \mathbb {R}^3$,as well as $\sigma _\infty (p) = \sinh \mu _\infty (p)$, $\gamma _\infty (p) = \cosh \mu _\infty (p)$ and $\mu (p) = \mu _\infty (p) \chi (p)$, $\sigma (p) = \sinh \mu (p)$ and $\gamma (p) = \cosh \mu (p)$. Notice that

42 $$\begin{aligned} \mu (p) = \left\{ \begin{array}{ll} 0 & \text { if } |p |\le N^{\kappa /2-\beta /4}, \\ \mu _{\infty } (p) & \text { if } |p | \ge 2 N^{\kappa /2-\beta /4} \end{array} \right. \end{aligned}$$

and also that $|\mu (p)| \le |\mu _\infty (p)|$ for all $p \in \mathbb {R}^3$. This implies that $|\sigma (p)| \le |\sigma _\infty (p)|$, $0 \le \gamma (p) - 1 \le \gamma _\infty (p) - 1$, σ(p)=0 and γ(p)=1 for $|p| < N^{\kappa /2-\beta /4}$.

In the next lemma, we collect various properties of the coefficients $\sigma _p, \gamma _p$ and of the corresponding Fourier series.

#### Lemma 5

In momentum space we have the bounds43which imply

44 $$\begin{aligned} \Vert \sigma \Vert _\infty , \Vert \gamma -1 \Vert _\infty \le CN^{\beta /8}, \end{aligned}$$

45 $$\begin{aligned} \sum _{p\in \Lambda ^*} |\sigma _p |, \sum _{p\in \Lambda ^*} |\gamma _p-1 | \le C N \end{aligned}$$

and, for s∈(3/2,6),

46 $$\begin{aligned} \sum _{p\in \Lambda ^*} |\gamma _{p}-1 |^s \le \sum _{p\in \Lambda ^*} |\sigma _{p} |^s \le C N^{3\kappa /2}. \end{aligned}$$

Furthermore, we have the following bounds in position space:474849and, for any m≥2, there exists $C_m > 0$ such that50where $\ell _\sigma =N^{-\kappa /2+\beta /4}$.

*Remark.* The decay $|x|^{-5/2}$ in (49) corresponds to the bound proportional to $|p|^{-1/2}$ in (43).

#### Proof

The bounds in momentum space follow similarly to Lemma 3, since $|\sigma (p)| \le |\sigma _\infty (p)|$, $|\gamma (p) - 1| \le |\gamma _\infty (p) - 1|$.

To establish the estimates in position space, we study the derivatives of $\mu (p), \sigma (p), \gamma (p)$. First, we observe that $|\mu (p)| \le C \log N$, for $|p| < N^{\kappa /2}$, and $|\mu (p)| \le C N^\kappa / |p|^2$, for $|p| > N^{\kappa /2}$. Hence, we have

51 $$\begin{aligned} |\mu (p) | \le C (\log N) \frac{N^\kappa }{N^\kappa +p^2} \end{aligned}$$

for all $p \in \mathbb {R}^3$. Also,52Thus, recalling the definition (34), we find, for any integer m≥1,Setting $n = n_1 + \dots + n_k$ and absorbing combinatorial factors from the sums over $m_1, \dots , m_k \ge 1$ with $m_1 + \dots +m_k = m$ and over $n_1, \dots , n_k \ge 0$ with $n_1 + \dots + n_k = n \le m$, we arrive at

53 $$\begin{aligned} \begin{aligned} |\partial _{p_j}^m\mu _\infty (p) |&\le C_m F_{m}(p/N^{1-\kappa }) \frac{N^\kappa }{p^2+N^\kappa }\sum _{n=0}^{m} |p |^{-n} N^{-(1-\kappa ) (m-n)}. \end{aligned} \end{aligned}$$

Recalling that $\chi (p) = \chi _l (|p|/N^{\kappa /2-\beta /4})$, we find, for integer m≥1,

54 $$\begin{aligned} \begin{aligned} |\partial _{p_j}^m \chi (p) |&\le C_m N^{-m (\kappa /2 - \beta /4)}\mathbb {1}_{[N^{\kappa /2-\beta /4}, 2N^{\kappa /2-\beta /4}]}(|p |) \\  &\le C_m |p |^{-m} \mathbb {1}_{[N^{\kappa /2-\beta /4}, 2N^{\kappa /2-\beta /4}]}(|p |) \,. \end{aligned} \end{aligned}$$

Combining this bound with (53), we obtain, for m≥0,

$$\begin{aligned} |\partial _{p_j}^m\mu (p) |&\le C_m F_{m}(p/N^{1-\kappa }) (\log N) \frac{N^\kappa }{N^\kappa +p^2} \\ \times \mathbb {1}(|p |&\ge N^{\kappa /2-\beta /4}) \sum _{n=0}^{m} |p |^{-n} N^{-(1-\kappa ) (m-n)} \end{aligned}$$

where the factor (logN) comes from (51), to handle the contribution in which all derivatives hit χ. We apply now the last bound to control derivatives of $\sigma (p), \gamma (p)$. For m≥0, we obtain

$$\begin{aligned} \begin{aligned} |\partial _{p_j}^m\sigma (p) | \le \;&C_m \sum _{k=1}^m |\sinh ^{(k)}(\mu (p)) | \sum _{\begin{array}{c} m_1,\dots ,m_k\ge 1: \\ \sum _{i=1}^k m_i =m \end{array}} \prod _{i=1}^k |\partial _{p_j}^{m_i} \mu (p) |\\ \le \;&C_m \left( 1+\min \left( \frac{N^{\kappa /4}}{|p |^{1/2}}, \frac{N^\kappa }{|p |^{2}} \right) \right) (\log N)^m\frac{N^\kappa }{N^\kappa +p^2} \, F_{m}(p/N^{1-\kappa }) \\  &\quad \times \sum _{n=0}^{m} |p |^{-n} N^{-(1-\kappa ) (m-n)} \mathbb {1}(|p |\ge N^{\kappa /2-\beta /4}) \end{aligned} \end{aligned}$$

where we used (43) to estimate $|\sinh ^{(k)} (\mu (p))|$ and the bound $\prod _{j=1}^k F_{m_j} (p) \le F_m (p)$, if $m_1 + \dots + m_k = m$ (several combinatorial factors, arising from sums over $m_1, \dots , m_k \ge 1$ with fixed $m = m_1 +\dots +m_k$ and also from sums over $n_1, \dots , n_k \ge 0$ with fixed $n_1 + \dots + n_k = n$ have been absorbed in the constant $C_m$). Distinguishing $|p| > N^{\kappa /2}$ and $|p| \le N^{\kappa /2}$ and using the factor $N^\kappa / (N^\kappa + p^2)$ as well as using Young’s inequality for the sum over *n*, we obtain

55 $$\begin{aligned} \begin{aligned} |\partial _{p_j}^m\sigma (p) |&\le C_m (\log N)^m \min \left( \frac{N^{\kappa /4}}{|p |^{1/2}}, \frac{N^\kappa }{|p |^{2}} \right) F_{m}(p/N^{1-\kappa }) \mathbb {1}(|p |\ge N^{\kappa /2-\beta /4}) \\  &\quad \times \big (|p|^{-m}+N^{-m(1-\kappa )}\big ). \end{aligned} \end{aligned}$$

Analogously, we find

$$\begin{aligned} \begin{aligned} |\partial _{p_j}^m\gamma (p) |&\le C_m (\log N)^m \min \left( \frac{N^{\kappa /4}}{|p |^{1/2}}, \frac{N^\kappa }{|p |^{2}} \right) F_{m}(p/N^{1-\kappa }) \mathbb {1}(|p |\ge N^{\kappa /2-\beta /4}) \\&\quad \times \big (|p|^{-m}+N^{-m(1-\kappa )}\big ) \,. \end{aligned} \end{aligned}$$

Now we apply these bounds to show the pointwise bounds in position space. The bounds (47) follow immediately from (45). Next, we observe that, for $x\in \Lambda = [-1/2; 1/2]^3$ and any integer m≥1, we have

56 $$\begin{aligned} \begin{aligned} c^m |x_j |^m |\check{\sigma }(x) |&\le |(e^{2\pi i x_j}-1)^m\check{\sigma }(x) | = |(e^{2\pi i x_j}-1)^m \sum _{p\in \Lambda ^*} \sigma (p)e^{ip\cdot x} |\\&= |(e^{2\pi i x_j}-1)^{m-1} \sum _{p\in \Lambda ^*} (\sigma (p-2\pi e_j)-\sigma (p))e^{ip\cdot x} |\\&= \Big | \int _0^{2\pi } ds \sum _{p\in \Lambda ^*} (e^{2\pi i x_j}-1)^{m-1} \partial _{p_j}\sigma (p- s e_j)e^{ip\cdot x} \Big | \\&\le \int _0^{2\pi } ds_1\dots \int _0^{2\pi } ds_m \sum _{p\in \Lambda ^*} \Big | \partial _{p_j}^m\sigma (p-\sum _{i=1}^m s_i e_j) \Big | \\&\le (2\pi )^{m-1} \int _0^{2\pi m} ds \sum _{p\in \Lambda ^*} | \partial _{p_j}^m\sigma (p-s e_j) |. \end{aligned} \end{aligned}$$

Taking m=1 and using (55), we find

$$\begin{aligned} \begin{aligned} |x_j ||\check{\sigma }(x) |&\le C (\log N) \int _0^{2\pi } ds \sum _{\begin{array}{c} p\in \Lambda ^*_+: \\ |p-se_j| \ge N^{\kappa /2-\beta /4} \end{array}} \frac{N^{\kappa }(N^{-(1-\kappa )}+|p-s e_j |^{-1})}{|p- s e_j |^2} \\&\quad \times F_1((p-s e_j)/ N^{1-\kappa }). \end{aligned} \end{aligned}$$

We divide the sum into the two regions $N^{\kappa /2 - \beta /4} \le |p-s e_j| \le N^{1-\kappa }$ and $|p-se_j| > N^{1-\kappa }$. In the first region, we bound $F_1$ in the $\ell ^\infty$-norm. In the second region, we use instead the $\ell ^2$-norm of $F_1$. With (36), we obtain

$$\begin{aligned} |x_j ||\check{\sigma }(x) | \le C (\log N)^2 N^\kappa . \end{aligned}$$

Combining the bounds with different choices of *j*, we obtain

57 $$\begin{aligned} |\check{\sigma }(x) | \le C (\log N)^2 N^\kappa |x |^{-1}. \end{aligned}$$

Since the estimate for γ-1 can be shown similarly, this concludes the proof of (48).

Next, we prove (49). To this end, we can restrict our attention to $|x| \ge N^{-\kappa /2}$ (for $|x| < N^{-\kappa /2}$, (48) is stronger). We split $\check{\sigma }(x) = \check{\sigma }_{1} (x) + \check{\sigma }_{2} (x)$, with

$$\begin{aligned} \check{\sigma }_{1} (x) = \sum _{p\in \Lambda ^*} \sigma (p) (1-\chi _l (|p ||x |)) e^{ip \cdot x}, \qquad \check{\sigma }_2 (x) = \sum _{p\in \Lambda ^*} \sigma (p) \chi _l (|p ||x |) e^{ip\cdot x}.\end{aligned}$$

Recalling that σ(p)=0 for $|p| < N^{\kappa /2 - \beta /4}$, we have

$$\begin{aligned} |\sigma (p) |(1-\chi _l(|p | |x |)) \le |\sigma (p) | \mathbb {1}_{[N^{\kappa /2-\beta /4}, 2|x |^{-1}]}(|p |). \end{aligned}$$

With the bound (43), we immediately obtain

58 $$\begin{aligned} |\check{\sigma }_{1} (x) | \le C \sum _{\begin{array}{c} p\in \Lambda _+^*: \\ N^{\kappa /2-\beta /4}\le |p | \le 2|x |^{-1} \end{array}} N^{\kappa /4}|p |^{-1/2} \le C N^{\kappa /4} |x |^{-5/2}. \end{aligned}$$

As for the second term, note that, for integer m≥0,

59 $$\begin{aligned} \big | {\partial _{p_j}^m \chi _l(|p ||x |)}\big | \le C_m |p |^{-m}\mathbb {1}(|p |\ge |x |^{-1}), \end{aligned}$$

and therefore

60 $$\begin{aligned} \begin{aligned} |\partial _{p_j}^m(\sigma (p)\chi _l(|p ||x |)) |&\le C_m (\log N)^m \min \Big ( \frac{N^{\kappa /4}}{|p |^{1/2}}, \frac{N^\kappa }{|p |^{2}} \Big ) \\&\quad \times F_{m}(p/N^{1-\kappa }) \mathbb {1}(|p |\ge |x |^{-1}) \big (|p|^{-m}+N^{-m(1-\kappa )}\big ). \end{aligned} \end{aligned}$$

Proceeding as in (56), we find

$$\begin{aligned} \begin{aligned} c^3 |x_j |^3 |\check{\sigma }_{2} (x) |&\le (2\pi )^2 \int _0^{6\pi } ds \sum _{p\in \Lambda ^*} |\partial _{p_j}^3(\sigma \chi _l(\cdot |x |))(p- s e_j) |\\&\le C_3 (\log N)^3 \int _0^{6\pi } ds \sum _{\begin{array}{c} p\in \Lambda ^*\\ |p-s e_j |\ge |x |^{-1} \end{array}} \frac{N^{\kappa /4}}{ |p-s e_j |^{7/2}} F_{3} ((p-se_j)/N^{1-\kappa }) \\&\quad +C_3(\log N)^3\int _0^{6\pi }ds \sum _{\begin{array}{c} p\in \Lambda ^*\\ |p- s e_j | \ge |x|^{-1} \end{array}} \frac{N^{\kappa }}{|p-se_j|^2} F_3 ((p-s e_j)/N^{1-\kappa }) N^{-3(1-\kappa )}. \end{aligned} \end{aligned}$$

Estimating $F_3$ with its $\ell ^\infty$-norm (in the first term) and its $\ell ^2$-norm (in the second term) and applying Lemma 4, we obtain

$$\begin{aligned} |x_j |^3 |\check{\sigma }_{2} (x) | \le C (\log N)^3 N^{\kappa /4} |x|^{1/2}.\end{aligned}$$

Combined with (58), this implies (49) (the bound for 1-γ can be shown similarly).

Finally, we show (50). Here, it is enough to consider $|x| > \ell _\sigma = N^{-\kappa /2 + \beta /4}$ and m≥2. Then, we have, combining (56) and (55),

$$\begin{aligned} \begin{aligned} c^m&|x_j |^m |\check{\sigma }(x) | \\&\quad \le (2\pi )^m\int _0^{2\pi m} ds \sum _{p\in \Lambda ^*} |(\partial _{p_j}^m\sigma (p- s e_j) |\\&\quad \le C_m (\log N)^m \int _0^{2\pi m} ds \sum _{\begin{array}{c} p\in \Lambda ^*\\ |p-s e_j | \ge N^{\kappa /2-\beta /4} \end{array}} \frac{N^\kappa }{|p- s e_j |^{2}} F_m((p-s e_j)/N^{1-\kappa }) N^{-(1-\kappa ) m}\\&\qquad + C_m (\log N)^m \int _0^{2\pi m} ds \sum _{\begin{array}{c} p\in \Lambda ^*\\ |p- s e_j | \ge N^{\kappa /2-\beta /4} \end{array}} \frac{N^\kappa }{ |p- s e_j |^{m+2} } F_m ((p-se_j)/N^{1-\kappa }). \end{aligned} \end{aligned}$$

Estimating $F_m$ with its $\ell ^2$-norm in the first term and with its $\ell ^\infty$-norm in the second term, we conclude that

$$\begin{aligned} \begin{aligned} c^m |x_j |^m |\check{\sigma }(x) |&\le C_m (\log N)^{m} N N^{-m(\kappa /2-\beta /4)}. \end{aligned} \end{aligned}$$

Bounds for γ-1 can be proven analogously; this implies (50). □

In our analysis of the action of the cubic transformation $e^{A^* \Theta - \Theta A}$, it will often be convenient to localize kernels in small boxes, to take advantage of the presence of the cutoff Θ, controlling the local number of particles. Recalling that $\beta = 2-3\kappa > 0$, we introduce the length scale $\ell _B = N^{-\kappa /2 + \beta /12}$. We consider the lattice $\Lambda _B = \Lambda \cap \ell _B \mathbb {Z}^3$ and, for $u\in \Lambda _B$, we define the box $B_u = \{x\in \Lambda , \inf _{v\in \Lambda _B} |x-v | =|x-u | \}$. We denote by $\check{\sigma }_u = \check{\sigma }\mathbb {1}_{B_u}$ and  the restrictions of $\check{\sigma }$ and  on $B_u$. Clearly, $\check{\sigma }= \sum _{u \in \Lambda _B} \check{\sigma }_u$ and .

#### Lemma 6

For every ϵ>0, there is C>0 such that61and62

*Remark.* Eq. (61) implies that $\sum _{u \in \Lambda _B} \Vert \check{\sigma }_u \Vert _2$ is comparable (up to the additional factor $N^\epsilon$) with $\Vert \check{\sigma } \Vert _2$. In fact, $\ell _B$ is the smallest length scale with this property (this is the reason for fixing $\ell _B = N^{-\kappa /2 + \beta /12}$).

#### Proof

We only show the bounds for $\check{\sigma }$, the others follow analogously. For any δ>0, we have

$$\begin{aligned} \begin{aligned}&\sum _{u\in \Lambda _B} \Vert \check{\sigma }_u \Vert _2 = \Vert \check{\sigma }_0 \Vert _2 + \sum _{0\ne u\in \Lambda _B, |u |\le \ell _\sigma N^\delta } \Vert \check{\sigma }_u \Vert _2 + \sum _{u\in \Lambda _B, |u |> \ell _\sigma N^\delta } \Vert \check{\sigma }_u \Vert _2. \end{aligned} \end{aligned}$$

The first term can be bounded by $\Vert \check{\sigma }_0 \Vert _2 \le \Vert \check{\sigma } \Vert _2 \le C N^{3\kappa /4}$, using (46). To estimate the second and third terms, we use the pointwise bounds (49) and, respectively, (50). We find, for any integer m≥2,

$$\begin{aligned} \begin{aligned} \sum _{u\in \Lambda _B} \Vert \check{\sigma }_u \Vert _2 \le \;&C N^{3\kappa /4} + C (\log N)^3 \sum _{0\ne u\in \Lambda _B, |u |\le \ell _\sigma N^\delta } N^{\kappa /4} \ell _B^{3/2} |u |^{-5/2} \\  &+ C \sum _{u\in \Lambda _B, |u |> \ell _\sigma N^\delta } N N^{-\delta m} \ell _B^{3/2} \\ \le \;&C N^{3\kappa /4} + C (\log N)^3 N^{\kappa /4} \ell _\sigma ^{1/2} N^{\delta /2} \ell _B^{-3/2} + C N N^{-\delta m} \ell _B^{-3/2}\\ \le \;&C (\log N)^3 N^{3\kappa /4} N^{\delta /2} + C N N^{-\delta m} \ell _B^{-3/2}. \end{aligned} \end{aligned}$$

Choosing δ sufficiently small and then *m* sufficiently large, we obtain (61). To prove (62), we observe that $\sum _{u \in \Lambda _B} \Vert \check{\sigma }_u \Vert _1 \le \Vert \check{\sigma } \Vert _1$ and we use the pointwise bounds (49) for $|x| \le N^{-\kappa /2 + \beta /4 + \delta }$ and (50) for $|x| > N^{-\kappa /2 + \beta /4 + \delta }$. □

### Coefficients of the cubic transformation

In this subsection, we are going to introduce the coefficients $A_{p,q}$, defining the cubic transformation (23). We proceed similarly as in [10]. From (40), we recall the cutoff function $\chi _l \in C^{\infty }(\mathbb {R})$, satisfying $0\le \chi _l \le 1$, $\chi _l(x)=0$ if x≤1, $\chi _l(x)=1$ if x≥2. We define $\tilde{\chi } (p) = \chi _l (|p |/N^{\kappa /2})$ and, for every $p, q \in \mathbb {R}^3 \backslash \{ 0 \}$,63with σ(q) as defined above (42) and64Notice that the infrared cutoffs act differently in the two variables. In *p*, we restrict to momenta larger than $N^{\kappa /2}$. In *q*, on the other hand, we have to consider momenta larger than $N^{\kappa /2 - \beta /4}$. For $p,q \in \Lambda ^*_+$, we also use the notation $\eta _p = \eta (p)$ and $A_{p,q} = A (p,q)$. For completeness, we set $A_{p,q} = 0$, if p=0 or q=0. In position space, we denote

65 $$\begin{aligned} \check{A}(x,y) = \sum _{p,q \in \Lambda ^*} A_{p,q} e^{ipx}e^{iqy}. \end{aligned}$$

Several properties of the coefficients $A_{p,q}, \eta _p$ and of the function $\check{A}$ are collected in the next lemma.

#### Lemma 7

First, we list properties in momentum space. We have66and $\eta _p = 0$ for $|p| < N^{\kappa /2}$, as well as

67 $$\begin{aligned} \Vert \eta \Vert _\infty \le C, \quad \Vert \eta \Vert _2^2\le C N^{3\kappa /2}, \quad \Vert \eta \Vert _1\le CN. \end{aligned}$$

Moreover68uniformly in *p*. Furthermore, we have the bounds

69 $$\begin{aligned} \sum _{p\in \Lambda ^*}|\eta _p-\sigma _p | \le C N^{3\kappa /2}, \qquad \sum _{p\in \Lambda ^*}p^4|\eta _p-\sigma _p |^2 \le C N^{7\kappa /2}. \end{aligned}$$

The coefficients $A_{q,p}$ satisfy

70 $$\begin{aligned} |A_{p,q} | \le 2N^{-1/2}|\eta _p ||\sigma _q | \end{aligned}$$

for all $p, q\in \Lambda ^*$. Moreover,

71 $$\begin{aligned} \sum _{p\in \Lambda ^*} |A_{p,-p-q} | \le C N^{2\kappa -1/2} |q |^{-1} \end{aligned}$$

for all $q \in \Lambda ^*$, and

72 $$\begin{aligned} \sum _{p,q\in \Lambda ^*}|A_{p,q} |^2 \le C N^{3\kappa -1}. \end{aligned}$$

In position space, there is C>0 and, for all m≥2, $C_m > 0$ such that

73 $$\begin{aligned} \begin{aligned} |\check{A}(x,y) | \le&C N^{-1/2} \min \Big ( N, (\log N) \frac{N^\kappa }{|x |}, C_m N \Big (\frac{\ell _\eta }{|x |}\Big )^m\Big ) \\&\times \min \left( N, (\log N)^2 \frac{N^\kappa }{|y |}, (\log N)^3 \frac{N^{\kappa /4}}{|y |^{5/2}}, C_m N \left( \frac{\ell _\sigma }{|y |}\right) ^m\right) \end{aligned} \end{aligned}$$

where $$\ell _\eta =N^{-\kappa /2}$$ and $$\ell _\sigma =N^{-\kappa /2+\beta /4}$$.

#### Proof

By the definition of $\eta _p$ we have $\eta _p = 0$ for $|p| < N^{\kappa /2}$. Together with the trivial bound (66), we immediately find (67). The bound (70) follows directly from the definition of $A_{p,q}$, and (71) and (72) are a consequence of (70). Equation (68) follows from (37) (whose second line is shown to be $O(N^{2\kappa -1})$), because

$$\begin{aligned} \frac{N^\kappa }{N} \sum _{q \in \Lambda ^*_+} |\eta _q - \eta _{\infty , q}| \le C\frac{N^\kappa }{N} \sum _{|q| < 2 N^{\kappa /2}} \frac{N^\kappa }{q^2} \le C N^{5\kappa /2 -1}. \end{aligned}$$

To prove (69), we observe that, from Lemma 3,

$$\begin{aligned} \begin{aligned} |\eta _p - \sigma _p|&\le |\eta _p - \eta _{\infty ,p}| + |\sigma _p - \sigma _{\infty ,p}| + |\eta _{\infty ,p} - \gamma _{\infty ,p} \sigma _{\infty ,p}| + |\sigma _{\infty ,p}| | \gamma _{\infty ,p} - 1| \\  &\le C \min \Big ( \frac{N^\kappa }{p^2}, \frac{N^{2\kappa }}{|p|^4} \Big ). \end{aligned} \end{aligned}$$

As for the position space bound (73), we proceed similarly as in the proof of Lemma 5. Analogously to (56) we find

74 $$\begin{aligned} c^m c^l |x_j |^m |y_k |^l|\check{A} (x,y) | \le C \int _0^{2\pi m} ds \int _0^{2\pi l} dt \sum _{p,q\in \Lambda ^*} |(\partial _{p_j}^m\partial _{q_k}^l A (p- s e_j, q- t e_k) |\nonumber \\ \end{aligned}$$

which again allows to relate position space decay to derivatives in momentum space. For the derivatives of the kernel we findRecalling the definition (34) and the bound (55) for the derivatives of σ(q), we obtain

$$\begin{aligned} \begin{aligned} |\partial _{p_j}^m\partial _{q_k}^l A (p, q) |&\le C_{m,l} N^{-1/2+\kappa } \\  &\quad \times \sum _{\begin{array}{c} m_i\ge 0\\ m_1+m_2+m_3=m \end{array}} |p |^{-(m_1+ m_3 +2)} \mathbb {1}(|p |\ge N^{\kappa /2}) N^{-(1-\kappa ) m_2} F_m (p/N^{1-\kappa }) \\&\quad \times \sum _{\begin{array}{c} l_i\ge 0\\ l_1+l_2 =l \end{array}} |q |^{- l_1} (\log N)^{l_2} \min \Big ( \frac{N^{\kappa /4}}{|q |^{1/2}}, \frac{N^\kappa }{|q |^{2}} \Big ) F_{l_2}(q/N^{1-\kappa }) \\  &\quad \times \sum _{l_3 =0}^{l_2} |q |^{-l_3} N^{-(1-\kappa ) (l_2-l_3)} \mathbb {1}(|q |\ge N^{\kappa /2-\beta /4}). \end{aligned} \end{aligned}$$

Setting $n = m_1+m_3$, $i= l_1+l_3$ (and absorbing appropriate combinatorial factors in the constant $C_{m,l}$), we arrive at

$$\begin{aligned} \begin{aligned} \big | \partial _{p_j}^m&\partial _{q_k}^l A (p, q) \big | \\&\le C_{m,l} N^{-1/2} \frac{N^\kappa }{|p |^2} F_m(p/N^{1-\kappa }) \mathbb {1}(|p |\ge N^{\kappa /2}) \sum _{n=0}^m |p |^{-n} N^{-(1-\kappa ) (m-n)} \\&\quad \times (\log N)^l \min \Big ( \frac{N^{\kappa /4}}{|q |^{1/2}}, \frac{N^\kappa }{|q |^{2}} \Big ) F_{l}(q/N^{1-\kappa }) \mathbb {1}(|q |\\&\ge N^{\kappa /2-\beta /4}) \sum _{i=0}^l |q |^{-i} N^{-(1-\kappa ) (l-i)}. \end{aligned} \end{aligned}$$

Since the r.h.s. is a product of a function of *p* with a function of *q*, we can apply this bound to (74) and proceed exactly as in the proof of Lemma 5, decoupling the variables *x* and *y*. □

Similarly as discussed before Lemma 6 for $\check{\sigma }$, it will also be useful to decompose $\check{A}$ into a sum of functions $\check{A}_{u,v}$ supported in small boxes around the points $u,v \in \Lambda _B$ (this will allow us to take advantage of the cutoff Θ on the local number of particles).

#### Lemma 8

Recall that $\ell _B = N^{-\kappa /2 + \beta /12}$, $\Lambda _B = \Lambda \cap \ell _B \mathbb {Z}^3$ and that, for $u\in \Lambda _B$, we defined $B_u = \{x\in \Lambda , \inf _{v\in \Lambda _B} |x-v | =|x-u | \}$. We denote by $\check{A}_{u,v} = \check{A} (\mathbb {1}_{B_u}\otimes \mathbb {1}_{B_v})$ the restriction of $\check{A}$ to $B_u \times B_v$, so that $\check{A} = \sum _{u, v \in \Lambda _B} \check{A}_{u,v}$. Then, for every ϵ>0, there exists C>0 such that

75 $$\begin{aligned} \sum _{u,v \in \Lambda _B} \Vert \check{A}_{u,v} \Vert _2 \le C N^{(3\kappa -1)/2 +\epsilon }. \end{aligned}$$

#### Proof

We proceed as in the proof of Lemma 6, decomposing

$$\begin{aligned} \begin{aligned} \sum _{u\in \Lambda _B} \Vert \check{A}_{u,v} \Vert _2&= \Vert \check{A}_{0,0} \Vert _2 + \sum _{0\ne v\in \Lambda _B, |v |\le \ell _\sigma N^\delta } \Vert \check{A}_{0,v} \Vert _2 + \sum _{v\in \Lambda _B, |v |> \ell _\sigma N^\delta } \Vert \check{A}_{0,v} \Vert _2\\&\quad +\sum _{0\ne u\in \Lambda _B} \Vert \check{A}_{u,0} \Vert _2 + \sum _{0\ne u,v \in \Lambda _B, |v |\le \ell _\sigma N^\delta } \Vert \check{A}_{u,v} \Vert _2\\&\quad + \sum _{0\ne u, v\in \Lambda _B, |v |> \ell _\sigma N^\delta } \Vert \check{A}_{u,v} \Vert _2. \end{aligned} \end{aligned}$$

We estimate $\Vert \check{A}_{0,0} \Vert _2 \le \Vert \check{A} \Vert _2 \le C N^{(3\kappa -1)/2}$ by (72). For the other terms, we use the pointwise bound (73). To estimate $\Vert \check{A}_{0,v} \Vert _2$, for $|v| \le \ell _\sigma N^\delta$, we divide the integral over $x \in B_0$ in the two regions $|x| \le \ell _\eta N^\delta$ (where we use the bound for $\check{A} (x,y)$ proportional to $N^\kappa / |x|$) and $|x| > \ell _\eta N^\delta$ (where we use the arbitrarily fast decay $(\ell _\eta / |x|)^m$). We find

$$\begin{aligned} \begin{aligned} \Vert \check{A}_{0,v} \Vert _2&\le C (\log N)^4 N^{-1/2 + 5\kappa /4} |v|^{-5/2} \ell _B^{3/2} \Big [ \int _{|x| \le \ell _\eta N^\delta } |x|^{-2} dx + N^{1-\delta m} \ell _B^3 \Big ]^{1/2} \\  &\le C (\log N)^4 N^{-1/2 +5\kappa /4} |v|^{-5/2} \ell _B^{3/2} \big [ \ell _\eta ^{1/2} N^{\delta /2} + N^{(1-\delta m)/2} \ell _B^{3/2} \big ] \end{aligned} \end{aligned}$$

for all $|v| \le \ell _\sigma N^\delta$. Hence

$$\begin{aligned} \sum _{0\ne v \in \Lambda _B, |v| \le \ell _\sigma N^\delta } \Vert \check{A}_{0,v} \Vert _2 \le C (\log N)^4 N^{-1/2 +5\kappa /4} \ell _\sigma ^{1/2} \ell _B^{-3/2} \ell _\eta ^{1/2} N^{\delta } \le C N^{(3\kappa -1)/2+ 2\delta }. \end{aligned}$$

For $|v| > \ell _\sigma N^\delta$ and also for all terms with u≠0, we can use the arbitrary fast decay of $\check{A} (x,y)$ (in *y*, for $|v| > \ell _\sigma N^\delta$, and in *x*, if u≠0). □

To describe commutators involving the cubic operator (23), it is useful to introduce the kernels

76 $$\begin{aligned} \begin{aligned} \check{A}^{(3)} (x,y)&= \check{A} (x,y) + \check{A} (y,x) + \check{A} (-y, x-y) \\ \check{A}^{(6)} (x,y)&= \check{A}^{(3)} (x,y) + \check{A}^{(3)} (-x, -x+y). \end{aligned} \end{aligned}$$

It is sometimes useful to use the notation $\check{A}^{(1)} = \check{A}$. Observe that, by definition,

77 $$\begin{aligned} \check{A}^{(6)} (x,y) = \check{A}^{(6)} (y,x) = \check{A}^{(6)} (x-y, -y) = \check{A}^{(6)} (y-x, -x). \end{aligned}$$

In Fourier space, we have

78 $$\begin{aligned} \begin{aligned} A^{(3)}_{p,q}&= A_{p,q} + A_{q,p} + A_{-p-q,p}, \\ A^{(6)}_{p,q}&= A^{(3)}_{p,q} + A^{(3)}_{-p-q,q}\\&= A_{p,q} + A_{q,p} + A_{-p-q,p} + A_{-p-q,q} + A_{q,-p-q} + A_{p,-p-q}. \end{aligned} \end{aligned}$$

From (77), we find

79 $$\begin{aligned} A^{(6)}_{p,q} = A^{(6)}_{q,p} = A^{(6)}_{-p-q, q} = A^{(6)}_{p, -p-q}. \end{aligned}$$

Additionally, from (63), we also have $A^{(j)}_{-p, -q} = A^{(j)}_{p,q}$, for j=1,3,6.

We collect some properties of these kernels in the next lemma.

#### Lemma 9

The Fourier coefficients $A^{(j)}_{p,q}$ satisfy

$$\begin{aligned} \sum _{p,q \in \Lambda ^*} |A^{(j)}_{p,q}|^2 \le C N^{3\kappa -1} \end{aligned}$$

for j=1,3,6. Moreover

80 $$\begin{aligned} \begin{aligned} \sum _{p \in \Lambda ^*} |A_{p,q}^{(j)}|^2&\le C N^{3\kappa /2 - 1} (1 + \sigma _q^2), \qquad \sum _{q \in \Lambda ^*} |A_{p,q}^{(j)}|^2 \le C N^{3\kappa /2 - 1} (1 + \sigma _p^2) \\ \sum _{p \in \Lambda ^*} |A_{p,q}^{(j)}|&\le C N^{1/2} (1 + |\sigma _q|), \quad \qquad \; \sum _{q \in \Lambda ^*} |A_{p,q}^{(j)}| \le C N^{1/2} (1 + |\sigma _p|) \end{aligned} \end{aligned}$$

for j=1,3,6. Hence

81 $$\begin{aligned} \sum _{p,q \in \Lambda ^*} (\sigma _p^2 + \sigma _q^2) |A^{(j)}_{p,q}|^2 \le C N^{3\kappa -1} \end{aligned}$$

for j=1,3,6.

We denote, as in Lemma 8, $\check{A}^{(j)}_{u,v} = \check{A}^{(j)} \, (\mathbb {1}_{B_u} \otimes \mathbb {1}_{B_v})$, for $u,v \in \Lambda _B = \Lambda \cap \ell _B \mathbb {Z}^3$ and for j=1,3,6, so that $\check{A}^{(j)} = \sum _{u,v \in \Lambda _B} \check{A}^{(j)}_{u,v}$. Then, we have

82 $$\begin{aligned} \sum _{u,v \in \Lambda _B} \Vert \check{A}^{(j)}_{u,v} \Vert _2 \le C N^{(3\kappa -1)/2+ \epsilon } \end{aligned}$$

for any ϵ>0 and for j=1,3,6.

We further define $D^{(j)} (x) = \sup _s |\check{A}^{(j)} (s,x)|$, $\tilde{D}^{(j)} (x) = \sup _s |\check{A}^{(j)} (x,s)|$ for *j* in $\{1,3,6\}$. Then,

$$\begin{aligned} \Vert \tilde{D}^{(j)} \Vert _2, \Vert D^{(j)} \Vert _2 \le C N^{3\kappa /4 + 1/2+ \epsilon } \end{aligned}$$

for any ϵ>0 and for j=1,3,6. Setting $D_u^{(j)} = D^{(j)} \, \mathbb {1}_{B_u}$ for $u \in \Lambda _B$, we find

$$\begin{aligned} \sum _{u \in \Lambda ^*} \Vert D^{(j)}_u \Vert _2 \le C N^{3\kappa /4 + 1/2 +\epsilon } \end{aligned}$$

for any ϵ>0.

*Remark.* We have introduced the kernels $D^{(j)}$ and $\tilde{D}^{(j)}$ in Lemma 9, as it is sometimes useful to take the supremum in one of the two variables of $\check{A}^{(j)}$.

#### Proof

The estimates in Fourier space follow from $|A_{p,q}| \le C N^{-1/2} |\eta _p| |\sigma _q|$ and from the estimates for $\eta _p, \sigma _q$ in Lemma 5 and Lemma 7 (recalling also that $\eta _p= 0$, if $|p| < N^{\kappa /2}$, and that $\sigma _q = 0$ if $|q| \le N^{\kappa /2 -\beta /4}$). To show (81), we use (46).

The bounds (82) can be proven proceeding similarly as in the proof of Lemma 8 (it is easy to check that the proof of Lemma 8 also extends to terms like $\check{A} (-y, x-y) \mathbb {1} (x \in B_u) \mathbb {1} (y \in B_v)$). Finally, the estimates for $D^{(j)}, \tilde{D}^{(j)}$ and their localized versions follow from the pointwise bound

83 $$\begin{aligned} \begin{aligned} |D^{(j)} (x)|, |\tilde{D}^{(j)} (x)| \!\le \! C N^{1/2} \min \big (N, (\log N)^2 \frac{N^\kappa }{|x|}, (\log N)^3 \frac{N^{\kappa /4}}{|x|^{5/2}}, N (\ell _\sigma / |x|)^m \big ),\end{aligned} \nonumber \\\end{aligned}$$

valid for j=1,3,6, which can be proven with (73). To show decay of terms like $\check{A} (-y,x-y)$ in the *x*-direction, we use that either |y|>|x|/2 or |x-y|>|x|/2 is always true. □

The commutator of the cubic transformation (23) with the kinetic energy operator is again a cubic operator, with the modified coefficientsTo handle this term, it will be useful to remove the cutoff $\tilde{\chi }$. To this end, we will need to control the expectation of the cubic operator, with coefficients84satisfying $A_{\mathcal {K}}^\textrm{low} (p,q) = 0$ if $|p| > 2 N^{\kappa /2}$. In the next lemma, we collect some bounds on (84), which will be used below, in the proof of Lemma 26.

#### Lemma 10

We have

85 $$\begin{aligned} \sum _{p,q\in \Lambda ^*} |A_{\mathcal {K}}^\textrm{low}(p,q) |^2 \le C N^{5\kappa -1}. \end{aligned}$$

Let $\check{A}_{\mathcal {K}}^\textrm{low}(x,y) = \sum _{p,q\in \Lambda ^*_+} A_{\mathcal {K}}^\textrm{low}(p,q) e^{ip\cdot x}e^{iq\cdot y}$. For every $m \in \mathbb {N}$, we find $C_m > 0$ such that

86 $$\begin{aligned} |\check{A}_{\mathcal {K}}^\textrm{low}(x,y) | \le C_m N^{5\kappa /2 - 1/2} \left( \frac{\ell _\eta }{|x |}\right) ^m |\check{\sigma }(y) |. \end{aligned}$$

Recall $\ell _B = N^{-\kappa /2 + \beta /12}$, $\Lambda _B = \Lambda \cap \ell _B \mathbb {Z}^3$. For $u\in \Lambda _B$, let $B_u = \{x\in \Lambda , \inf _{v\in \Lambda _B} |x-v | =|x-u | \}$ and denote by $\check{A}_{\mathcal {K},u,v}^\textrm{low} = \check{A}_{\mathcal {K}}^\textrm{low} (\mathbb {1}_{B_u}\otimes \mathbb {1}_{B_v})$ the restriction of $\check{A}_{\mathcal {K}}^\textrm{low}$ to $B_u \times B_v$. Then, for every ϵ>0 there is C>0 such that

87 $$\begin{aligned} \sum _{u,v \in \Lambda _B} \Vert \check{A}_{\mathcal {K},u,v}^\textrm{low} \Vert _2 \le C N^{(5\kappa -1)/2 +\epsilon }. \end{aligned}$$

#### Proof

From (43), we find

$$\begin{aligned} \sum _{p,q \in \Lambda ^*} |A_{\mathcal {K}}^\textrm{low} (p,q)|^2 \le C N^{-1+2\kappa } \sum _{|p| \le 2 N^{\kappa /2}, q\in \Lambda ^*} |\sigma _q|^2 \le C N^{5\kappa -1}. \end{aligned}$$

The bound (86) follows by noticing that $\check{A}_{\mathcal {K}}^\text {low} (x,y)$ is the product of a function of *x* (the Fourier series with coefficients ) with $\check{\sigma } (y)$ and by estimating(where we used the fact that $N^{-1+\kappa } \ll N^{-\kappa /2}$). Thus, proceeding as in (56) and recalling $\Vert F_m \Vert _\infty \le C_m$ from Lemma 4, we findwhich implies (86). With (86), the bound (87) follows, proceeding similarly as in the proof of Lemma 8. The main contributions to the sum are $\Vert \check{A}_{\mathcal {K},0,0}^\text {low} \Vert _2 \le \Vert \check{A}_{\mathcal {K}}^\text {low} \Vert _2 \le C N^{5\kappa -1}$ and the sum over $|v| \le \ell _\sigma N^\delta$ of $\Vert \check{A}_{\mathcal {K},0,v}^\text {low} \Vert _2$. Here, we integrate separately over $|x| \le \ell _\eta N^\delta$ (where we estimate $| \check{A}_{\mathcal {K}}^\text {low} (x,y)| \le C N^{(5\kappa -1)/2} |\check{\sigma } (y)|$) and over $|x| > \ell _\eta N^\delta$ (where we bound $| \check{A}_{\mathcal {K}}^\text {low} (x,y)| \le C N^{(5\kappa -1)/2-\delta m} |\check{\sigma } (y)|$). Using (49) to control $|\check{\sigma } (y)|$, we obtain

$$\begin{aligned} \begin{aligned}&\sum _{v \in \Lambda _B, |v| \le \ell _\sigma N^\delta } \Vert \check{A}_{\mathcal {K},0,v}^\text {low} \Vert _2 \\&\qquad \le C (\log N)^3 N^{(5\kappa -1)/2} N^{\kappa /4} \ell _B^{3/2} \left[ \ell _\eta ^3 N^{3\delta } + N^{-\delta m} \ell _B^3 \right] ^{1/2} \sum _{v\in \Lambda _B, |v| \le \ell _\sigma N^\delta } |v|^{-5/2} \\&\qquad \le C (\log N)^3 N^{(5\kappa -1)/2} N^{\kappa /4} \ell _B^{-3/2} \ell _\eta ^{3/2} \ell _\sigma ^{1/2} N^{2\delta } \le C N^{(5\kappa -1)/2+\epsilon } \end{aligned} \end{aligned}$$

for an arbitrarily small ϵ>0 (where we first chose δ>0 small enough and then $m \in \mathbb {N}$ sufficiently large). All other contributions can be bounded using the fast decay for $|x| > \ell _\eta N^\delta$ or for $|y| > \ell _\sigma N^\delta$. □

### The Local Particle Number Cutoff

We now define the cutoff Θ to be inserted in the cubic transformation appearing in (19) to control the local density of the excitations. We consider the length scale $\ell _{B} = N^{-\kappa /2+\beta /12}$, with $\beta = 2-3\kappa$ (the choice of $\ell _B$ is explained in the remark after Lemma 6). Through Θ, we will require that balls of radius $\ell _B$ only contain $N^\epsilon$ particles, for a small ϵ>0. Note that the typical number of cubic excitations in a ball with radius $\ell _B$ is of the order $\ell _B^3 N^{3\kappa -1} \simeq N^{-\beta /4} \ll 1$ (we will show that the total number of cubic excitations is of the order $\Vert A \Vert _2^2 \simeq N^{3\kappa -1}$). For this reason, Θ will not substantially change the energy of our trial state.

Pick a radial function $\chi _{\ell _B} \in C_c^\infty (\mathbb {R}^3)$ such that $0\le \chi _{\ell _B}\le 1$, $\chi _{\ell _B}(x)=1$ if $|x |\le \ell _B$ and $\chi _{\ell _B}(x)=0$ if $|x |\ge 2\ell _B$. Define

88 $$\begin{aligned} \mathcal {N}_{\chi _{\ell _B}} (w) :=\int dv \, \chi _{\ell _B}(w-v) a_v^* a_v. \end{aligned}$$

For ℓ>0, we also define the operator

89 $$\begin{aligned} \mathcal {N}_{\ell } (w) :=\int dv \mathbb {1}(|w-v |\le \ell ) \, a_v^* a_v \end{aligned}$$

counting the number of particles in a ball of radius ℓ around *w*. We think of $\mathcal {N}_{\chi _{\ell _B}} (w)$ as a smoothed out version of $\mathcal {N}_{\ell _B} (w)$. For $n \in \mathbb {N}$, we set

90 $$\begin{aligned} \mathcal {L}_n :=\int dw \, (\mathcal {N}_{\chi _{\ell _B}} (w) +1)^n. \end{aligned}$$

Choosing $\Theta \in C^\infty (\mathbb {R})$ monotonically decreasing with $0\le \Theta \le 1$, Θ(x)=1 if x≤1 and Θ(x)=0 if x≥2, and for α>0 to be specified later on, we define the cutoff $\Theta (\mathcal {L}_n/N^\alpha ) \,.$

Note that $[\mathcal {N}_{\chi _{\ell _B}} (w), a_x^*] = \chi _{\ell _B}(w-x)a_x^*.$ Hence, for every $k \in \mathbb {N}$, there is a $C_k > 0$ such that

91 $$\begin{aligned} C_k^{-1}\Vert \prod _{i=1}^k a_{x_i} (\mathcal {N}_{\chi _{\ell _B}} (w) +1)\zeta \Vert\le &   \Vert (\mathcal {N}_{\chi _{\ell _B}} (w) +1)\prod _{i=1}^k a_{x_i}\zeta \Vert \nonumber \\\le &   \Vert \prod _{i=1}^k a_{x_i} (\mathcal {N}_{\chi _{\ell _B}} (w) +1)\zeta \Vert . \end{aligned}$$

With the cutoff $\Theta (\mathcal {L}_n/N^\alpha )$, we introduce the unitary cubic transformation

92 $$\begin{aligned} \exp \Big (\int dxdydz \check{A}(x-y,x-z) a_x^* a_y^* a_z^* \Theta (\mathcal {L}_n/N^\alpha )-\text {h.c.} \Big ) =:\exp (A^*\Theta -\text {h.c.} )\nonumber \\ \end{aligned}$$

with the kernel $\check{A}$ defined in (65). We note that $A^* \Theta$ is a bounded (everywhere-defined) operator. Consequently, the expression $A^*\Theta -\text {h.c.}$ is anti-selfadjoint and its exponential is a unitary operator.

#### Lemma 11

There is a constant C>0 such that, for every ϵ>0 and α>0, and T>0, we have

93 $$\begin{aligned} \mathcal {N}_{k \ell _B} (w)\Theta (T^{-n}\mathcal {L}_n/N^\alpha ) \le C T k^3 N^{\epsilon } \Theta (T^{-n} \mathcal {L}_n/N^\alpha ) \end{aligned}$$

and also

94 $$\begin{aligned} \Vert \mathcal {N}_{k\ell _B}^{1/2}(w)\prod _{j=1}^m a_{x_j}\Theta (T^{-n}\mathcal {L}_n/N^\alpha )\zeta \Vert ^2 \le C T k^3 N^{\epsilon } \Vert \prod _{j=1}^m a_{x_j}\Theta (T^{-n}\mathcal {L}_n/N^\alpha )\zeta \Vert ^2\nonumber \\ \end{aligned}$$

for all *n* large enough, $w \in \Lambda$, k≥1, $\zeta \in \mathcal {F}(\Lambda )$ and $m \in \mathbb {N}$. Moreover, for every α>0, we have

95 $$\begin{aligned} \langle \exp (A^*\Theta -\text {h.c.} )\Omega , \mathcal {L}_n \exp (A^*\Theta -\text {h.c.} )\Omega \rangle&\le C,\end{aligned}$$

96 $$\begin{aligned} \Vert (1-\Theta (\mathcal {L}_n/N^\alpha ))\exp (A^*\Theta -\text {h.c.} )\Omega \Vert&\le C N^{-\alpha /2} \end{aligned}$$

for all *n* large enough. Also, for every α>0 we have the bound (with the non-optimal exponent m-mβ/2)

97 $$\begin{aligned} \langle \exp (s (A^*\Theta -\Theta A)) \zeta , \mathcal {N}^m \exp (s (A^*\Theta -\Theta A)) \zeta \rangle \le C N^{m - m\beta /2} + \langle \zeta , \mathcal {N}^m \zeta \rangle \end{aligned}$$

for all *n* large enough and all normalized $\zeta \in \mathcal {F}(\Lambda )$ and s∈[0,1].

Finally, for any ν>0, we have

98 $$\begin{aligned} \langle \exp (A^*\Theta -\text {h.c.} )\Omega , A^* (1- \Theta (\mathcal {L}_n / N^\alpha )) A \exp (A^*\Theta -\text {h.c.} )\Omega \rangle \le C N^{-\nu } \end{aligned}$$

if α>0 and then $n \in \mathbb {N}$ are chosen large enough.

*Remark.* As it is clear from their proof, the bounds (96) and (98) remain true if we replace 1-Θ by a sharp cutoff, ie.

$$\begin{aligned} \begin{aligned} \Vert \mathbb {1}_{[k, \infty )} (\mathcal {L}_n / N^\alpha ) \exp (A^* \Theta -\text {h.c.} ) \Omega \Vert&\le C N^{-\alpha /2} \\ \langle \exp (A^* \Theta -\text {h.c.} ) \Omega , A^* \mathbb {1}_{[k, \infty )} (\mathcal {L}_n / N^\alpha ) A \exp (A^* \Theta -\text {h.c.} ) \Omega \rangle&\le N^{-\nu } \end{aligned} \end{aligned}$$

for k>0. In fact, the last bounds also holds if we include factors of $(\mathcal {N}+1)$, ie.

$$\begin{aligned} \langle \exp (A^* \Theta -\text {h.c.} ) \Omega , A^* (\mathcal {N}+1)^m \mathbb {1}_{[k, \infty )} (\mathcal {L}_n / N^\alpha ) A \exp (A^* \Theta -\text {h.c.} ) \Omega \rangle \le N^{-\nu } \end{aligned}$$

if α and *n* are chosen large enough (depending on *m*).

#### Proof

We begin with (93). For arbitrary *w* and *y* with $|w-y |\le \ell _B/2$ we have

$$\begin{aligned} (\mathcal {N}_{\ell _B/2} (w)+1)^n \le (\mathcal {N}_{\chi _{\ell _B}} (y) +1)^n. \end{aligned}$$

Hence

$$\begin{aligned} \begin{aligned} \mathcal {L}_n&= \int dy \, (\mathcal {N}_{\chi _{\ell _B}} (y) +1)^n \ge \int _{|w-y| \le \ell _B/2} dy \, (\mathcal {N}_{\chi _{\ell _B}} (y) +1)^n \\  &\ge \int _{|w-y| \le \ell _B/2} dy \, (\mathcal {N}_{\ell _B/2} (w) +1)^n \ge C \ell _B^3 (\mathcal {N}_{\ell _B/2} (w) +1)^n. \end{aligned} \end{aligned}$$

Taking the *n*-th root and writing $\mathcal {N}_{\ell _B} (w)$ as the sum over the number of particles in finitely many balls of radius $\ell _B/2$, we obtain that, on the support of $\Theta (T^{-n}\mathcal {L}_n/N^\alpha )$,

$$\begin{aligned} \mathcal {N}_{\ell _B} (w) \le C T\ell _B^{-3/n}N^{\alpha /n} \le C T N^{\epsilon }. \end{aligned}$$

if *n* is large enough. Decomposing balls of radius $k \ell _B$ into balls of radius $\ell _B$, we find

$$\begin{aligned} \mathcal {N}_{k\ell _B} (w) \le C T k^3 N^{\epsilon }. \end{aligned}$$

if *n* is large enough. Similarly, we can show (94). In fact,

99 $$\begin{aligned}  &   \Vert \mathcal {N}_{k \ell _B}^{1/2}(w)\prod _{j=1}^m a_{x_j}\Theta (T^{-n}\mathcal {L}_n/N^\alpha )\zeta \Vert ^2\nonumber \\  &   \quad \le \Vert \mathcal {N}_{k\ell _B}^{1/2}(w)\Theta \Big (T^{-n} \int dy \, \big (\mathcal {N}_{\chi _{\ell _B}} (y) +1 +\sum _{j=1}^m \chi _{\ell _B}(y-x_m) \big )^n / N^\alpha \Big ) \, \prod _{j=1}^m a_{x_j}\zeta \Vert ^2\nonumber \\  &   \quad \le C T k^3 N^{\epsilon } \Vert \prod _{j=1}^m a_{x_j}\Theta (T^{-n}\mathcal {L}_n/N^\alpha )\zeta \Vert ^2. \end{aligned}$$

To prove (95), we set $\xi =\exp (A^*\Theta -\Theta A)\Omega$ and we observe that, splitting $\check{A}$ as we did in Lemma 8,

$$\begin{aligned} \begin{aligned}&|\langle \xi , [\mathcal {L}_n, \int dx_1 dx_2 dx_3 \check{A}(x_1-x_2,x_1-x_3) a_{x_1}^* a_{x_2}^* a_{x_3}^* \Theta (\mathcal {L}_n/N^\alpha )]\xi \rangle |\\&\quad \le \int dx_1 dx_2 dx_3 dw \, | \check{A}(x_1-x_2,x_1-x_3)| \, |\langle \xi ,[(\mathcal {N}_{\chi _{\ell _B}} (w) +1)^n, a_{x_1}^* a_{x_2}^* a_{x_3}^*] \Theta (\mathcal {L}_n/N^\alpha )\xi \rangle |\\&\quad \le \sum _{k=0}^{n-1}\int dx_1 dx_2 dx_3 dw \, |\check{A}(x_1-x_2,x_1-x_3)| \\  &\hspace{.4cm} \times \big | \langle \xi ,(\mathcal {N}_{\chi _{\ell _B}} (w) +1)^k \sum _{i=1}^3\chi _{\ell _B} (x_i-w) a_{x_1}^* a_{x_2}^* a_{x_3}^* (\mathcal {N}_{\chi _{\ell _B}} (w) +1)^{n-k-1} \Theta (\mathcal {L}_n/N^\alpha )\xi \rangle \big | \\&\le C \sum _{u,v\in \Lambda _B} \sum _{i=1}^3 \int dx_1 dx_2 dx_3 dw |\check{A}_{u,v}(x_1-x_2,x_1-x_3) | \chi _{\ell _B}(x_i-w)\\&\hspace{.4cm} \times \Vert a_{x_1} a_{x_2} a_{x_3} \Theta (4^{-n}\mathcal {L}_n/N^\alpha ) (\mathcal {N}_{\chi _{\ell _B}} (w) +1)^{(n-1)/2} \xi \Vert \Vert (\mathcal {N}_{\chi _{\ell _B}} (w) +1)^{(n-1)/2} \xi \Vert .\\ \end{aligned} \end{aligned}$$

Here we used the fact that Θ is monotonically decreasing

$$\begin{aligned} \Theta (\mathcal {L}_n/N^\alpha )\le \Theta \Big (4^{-n}\int dw \, (\mathcal {N}_{\chi _{\ell _B}} (w)+\sum _{i=1}^3\chi _{\ell _B} (x_i-w) +1)^n/N^\alpha \Big ) \end{aligned}$$

and the pull-through formula

$$\begin{aligned} \int dw \, (\mathcal {N}_{\chi _{\ell _B}} (w)+\sum _{i=1}^3\chi _{\ell _B} (x_i-w) +1)^n a_{x_1} a_{x_2} a_{x_3}=a_{x_1} a_{x_2} a_{x_3}\mathcal {L}_n. \end{aligned}$$

Cauchy–Schwarz yields

$$\begin{aligned} \begin{aligned}&|\langle \xi , [\mathcal {L}_n, \int dxdydz \check{A}(x_1-x_2,x_1-x_3) a_{x_1}^* a_{x_2}^* a_{x_3}^* \Theta (\mathcal {L}_n/N^\alpha )]\xi \rangle |\\&\le C \sum _{u,v\in \Lambda _B} \sum _{i=1}^3 \Big (\int dx_1 dx_2 dx_3 dw \, \chi _{\ell _B}(x_i-w)\mathbb {1}(|x_1-x_2-u |,|x_1-x_3-v |\le \ell _B)\\&\quad \times \Vert a_{x_1} a_{x_2} a_{x_3} \Theta (4^{-n}\mathcal {L}_n/N^\alpha ) (\mathcal {N}_{\chi _{\ell _B}} (w) +1)^{\frac{(n-1)}{2}} \xi \Vert ^2 \Big )^{1/2}\\&\quad \times \Big (\int dx_1 dx_2 dx_3 dw |\check{A}_{u,v}(x_1-x_2,x_1-x_3) |^2 \chi _{\ell _B}(x_i-w)\Vert (\mathcal {N}_{\chi _{\ell _B}} (w) +1)^{\frac{(n-1)}{2}} \xi \Vert ^2\Big )^{1/2}. \end{aligned} \end{aligned}$$

Consider, for example, the term associated with i=1 (the other terms can be handled similarly). For every $\alpha , \epsilon > 0$ we can bound this contribution by

100 $$\begin{aligned} \begin{aligned} C&\sum _{u,v\in \Lambda _B} \Vert \check{A}_{u,v} \Vert _2 \Big (\int dx_1 dw \chi _{\ell _B}(x_1 - w)\\&\quad \times \Vert \mathcal {N}_{\ell _B}^{1/2}(x_1 -u) \mathcal {N}_{6\ell _B}^{1/2}(x_1- v) a_{x_1} \Theta (4^{-n}\mathcal {L}_n/N^\alpha ) (\mathcal {N}_{\chi _{\ell _B}} (w) +1)^{\frac{(n-1)}{2}} \xi \Vert ^2 \Big )^{1/2}\\&\quad \times \Big (\int dx_1 dw \, \chi _{\ell _B}(x_1-w)\Vert (\mathcal {N}_{\chi _{\ell _B}} (w) +1)^{\frac{(n-1)}{2}} \xi \Vert ^2\Big )^{1/2} \\&\le C N^\epsilon \ell _B^{3/2} \sum _{u,v\in \Lambda _B} \Vert \check{A}_{u,v} \Vert _2 \Big (\int dw \, \Vert (\mathcal {N}_{\chi _{\ell _B}} (w) +1)^{\frac{n}{2}} \xi \Vert ^2 \Big )^{1/2} \\  &\quad \times \Big (\int dw \, \Vert (\mathcal {N}_{\chi _{\ell _B}} (w) +1)^{\frac{(n-1)}{2}}\xi \Vert ^2\Big )^{1/2}\\&\le C N^{- \beta /8 + \epsilon } \langle \xi , \mathcal {L}_n\xi \rangle \end{aligned} \end{aligned}$$

if *n* is large enough. Here, we used (94) twice, to control $\mathcal {N}_{\ell _B}^{1/2}(x_1 -u)$ and $\mathcal {N}_{\ell _B}^{1/2}(x_1- v)$, and we applied Lemma 8. Choosing $0< \epsilon < \beta /8$, we conclude that

$$\begin{aligned} \Big | \frac{d}{dt} \langle e^{t (A^* \Theta - \Theta A)} \Omega , \mathcal {L}_n e^{t (A^* \Theta - \Theta A)} \Omega \rangle \Big | \le C \langle e^{t (A^* \Theta - \Theta A)} \Omega , \mathcal {L}_n e^{t (A^* \Theta - \Theta A)} \Omega \rangle \end{aligned}$$

for every $t \in \mathbb {R}$. Applying Gronwall’s lemma in the interval t∈[0;1], we obtain (95). Equation (96) is an immediate consequence.

To show (97), we first observe that

$$\begin{aligned} \mathcal {N}\le C \ell _B^{-3} \int dw \, \mathcal {N}_{\chi _{\ell _B}} (w) \le C \ell _B^{-3} \mathcal {L}^{1/n}_n. \end{aligned}$$

Therefore, on the support of the cutoff Θ, we have

101 $$\begin{aligned} \mathcal {N}^m \le C \ell _B^{-3m} N^{m \epsilon } \le C N^{m (3\kappa /2 - \beta /4 +\epsilon )} \end{aligned}$$

for any ϵ>0, if *n* is large enough (depending on $\alpha , \epsilon$). Setting $\zeta _s = \exp (s (A^* \Theta - \Theta A)) \zeta$, we obtain

102 $$\begin{aligned} \begin{aligned}&\Big | \langle \exp (A^*\Theta -\text {h.c.} )\zeta , \mathcal {N}^m \exp (A^*\Theta -\text {h.c.} )\Omega \rangle - \langle \xi , \mathcal {N}^m \zeta \rangle \Big | \\&\quad \le C \int _0^1 ds \, \Big | \langle \zeta _s, \big [\mathcal {N}^m, A^* \big ] \Theta (\mathcal {L}_n / N^\alpha ) \zeta _s \rangle \Big | \\&\quad \le C \int _0^1 ds \, \int dx_1 dx_2 dx_3 \, \big | \check{A} (x_1 - x_2, x_1 - x_3) \big | \\&\qquad \times \Vert a_{x_1} a_{x_2} a_{x_3} \Theta (\mathcal {L}_n / N^\alpha ) (\mathcal {N}+ 1)^{(m-1)/2} \zeta _s \Vert \big | \Vert (\mathcal {N}+ 1)^{(m-1)/2} \Theta (\mathcal {L}_n / N^\alpha ) \zeta _s \Vert \\&\quad \le C N^{(3\kappa -1)/2 + \epsilon } \int _0^1 ds \, \Vert (\mathcal {N}+ 1)^{(m-1)/2} \Theta (\mathcal {L}_n / N^\alpha ) \zeta _s \Vert \Vert (\mathcal {N}+ 1)^{m/2} \Theta (\mathcal {L}_n / N^\alpha ) \zeta _s \Vert \end{aligned} \end{aligned}$$

for any ϵ>0, if *n* is large enough. Here we proceeded similarly as in (100), decomposing $\check{A}$ into a sum of $\check{A}_{u,v}$, for $u,v \in \Lambda _B$. With (101), we conclude that

$$\begin{aligned} \begin{aligned} \langle \exp (A^*\Theta -\text {h.c.} )&\zeta , \mathcal {N}^m \exp (A^*\Theta -\text {h.c.} )\zeta \rangle \\  &\le \langle \zeta , \mathcal {N}^m \zeta \rangle + C N^{(3\kappa -1)/2 + \epsilon } N^{(3\kappa /2-\beta /4 + \epsilon ) (m-1/2)} \\  &\le \langle \zeta , \mathcal {N}^m \zeta \rangle + C N^{m (1 - 3\beta /4 + 2\epsilon )} \le \langle \zeta , \mathcal {N}^m \zeta \rangle + C N^{m (1-\beta /2)} \end{aligned} \end{aligned}$$

if *n* is large enough (depending on α>0).

Finally, we prove (98). Since

$$\begin{aligned}  &   \int dw \, \big ( \mathcal {N}_{\chi _{\ell _B}} (w) + 1 \big )^n a_x^* a_y^* a_z^*\\  &   \quad = a_x^* a_y^* a_z^* \int dw \, \big ( \mathcal {N}_{\chi _{\ell _B}} (w) +\chi _{\ell _B} (x) +\chi _{\ell _B} (y) +\chi _{\ell _B} (z) + 1 \big )^n\end{aligned}$$

we find

$$\begin{aligned} \begin{aligned} \langle e^{A^* \Theta - \Theta A} \Omega , A^* (1-&\Theta ) A e^{A^* \Theta - \Theta A} \Omega \rangle \\= \;&\langle e^{A^* \Theta - \Theta A} \Omega , \mathbb {1}_{[1,\infty )} (\mathcal {L}_n / N^\alpha ) A^* (1-\Theta ) A e^{A^* \Theta - \Theta A} \Omega \rangle . \end{aligned} \end{aligned}$$

With $\Vert A^* (1-\Theta ) A (\mathcal {N}+1)^{-3} \Vert \le C N^\tau$, for some τ>0, we conclude

$$\begin{aligned}  &   \langle e^{A^* \Theta - \Theta A} \Omega , A^* (1- \Theta ) A e^{A^* \Theta - \Theta A} \Omega \rangle \\  &   \quad \le C N^\tau \Vert \mathbb {1}_{[1,\infty )} (\mathcal {L}_n / N^\alpha ) e^{A^* \Theta - \Theta A} \Omega \Vert \Vert \mathcal {N}^3 e^{A^* \Theta - \Theta A} \Omega \Vert . \end{aligned}$$

From (96) (the fact that here we have $\mathbb {1}_{[1,\infty )}$ rather than 1-Θ does not change the argument) and (97) we obtain

$$\begin{aligned} \langle e^{A^* \Theta - \Theta A} \Omega , A^* (1-\Theta ) A e^{A^* \Theta - \Theta A} \Omega \rangle \le C N^{-\nu } \end{aligned}$$

if α>0 and then $n \in \mathbb {N}$ are large enough. □

### The Number of Particles in the Trial State

With the coefficients $\mu _p$ from (40), the coefficients $A_{p,q}$ from (63) and the local particle number cutoff $\Theta = \Theta (\mathcal {L}_n / N^\alpha )$ introduced in Sect. 2.3, we can define the quadratic operator *B* and the cubic operator *A* as in (22) and (23) and the trial state

103 $$\begin{aligned} \psi _N = W_{N_0} e^{B-B^*} e^{A^* \Theta - \Theta A} \Omega \end{aligned}$$

as in (19), with $W_{N_0}$ the Weyl operator (20) generating the Bose–Einstein condensate. Here, we choose the condensate number of particles $N_0$ so that

$$\begin{aligned} N = N_0 + \sum _{p \in \Lambda _+^*} \sigma _p^2 \end{aligned}$$

with $\sigma _p = \sinh \mu _p$ as defined in (41) (the norm $\Vert \sigma \Vert _2^2$ is the number of excitations generated by the Bogoliubov transformation). Since, from Lemma 5, $\Vert \sigma \Vert _2^2 \le C N^{3\kappa /2} \ll N$ for κ<2/3, it is always possible to find such $N_0$.

In the next lemma, we check that, with these definitions, our trial state has the correct number of particles. In the next section, we compute its energy.

#### Lemma 12

Consider the trial state (103). Then, we have

104 $$\begin{aligned} \langle \Psi _N, \mathcal {N}\Psi _N \rangle \ge N, \quad \langle \Psi _N, \mathcal {N}^2 \Psi _N \rangle \le CN^2 \end{aligned}$$

if α>0 and then $n \in \mathbb {N}$ are large enough.

#### Proof

With (21), we have

$$\begin{aligned} \begin{aligned} \langle \Psi _N, \mathcal {N}\Psi _N \rangle&= \langle e^{B^*-B} e^{A^*\Theta -\text {h.c.} }\Omega , (\mathcal {N}+ N_0 + \sqrt{N_0} (a_0^* + a_0)) e^{B^*-B} e^{A^*\Theta -\text {h.c.} }\Omega \rangle \\  &= \langle e^{B^*-B} e^{A^*\Theta -\text {h.c.} }\Omega , (\mathcal {N}+ N_0) e^{B^*-B} e^{A^*\Theta -\text {h.c.} }\Omega \rangle \end{aligned} \end{aligned}$$

because the expectation of $a_0, a_0^*$ is zero in the state $e^{B^*-B} e^{A^*\Theta -\text {h.c.} }\Omega$. From (41), we find

105 $$\begin{aligned} e^{B-B^*} \mathcal {N}e^{B^* - B} = \sum _{p \in \Lambda _+^*} (\gamma _p^2 + \sigma _p^2) a_p^* a_p + \sum _{p \in \Lambda ^*_+} \gamma _p \sigma _p (a_p^* a_{-p}^* + a_p a_{-p}) + \sum _{p \in \Lambda _+^*} \sigma _p^2.\nonumber \\\end{aligned}$$

Since the expectation of $a_p^* a_{-p}^*$ vanishes in the state $e^{A^* \Theta - \text {h.c.} } \Omega$, we immediately conclude that

$$\begin{aligned} \langle \Psi _N, \mathcal {N}\Psi _N \rangle \ge N_0 + \sum _{p \in \Lambda ^*_+} \sigma _p^2 = N. \end{aligned}$$

On the other hand, recalling from Lemma 5 that $\Vert \sigma \Vert _\infty , \Vert \gamma \Vert _\infty \le C N^{\beta /8}$, (105) implies that

$$\begin{aligned} e^{B-B^*} \mathcal {N}e^{B^* - B} \le C N^{\beta /4} \mathcal {N}+ C N \,. \end{aligned}$$

Similarly,

$$\begin{aligned} e^{B-B^*} \mathcal {N}^2 e^{B^* - B} \le C N^{\beta /2} \mathcal {N}^2 + C N^2.\end{aligned}$$

Thus

$$\begin{aligned} \begin{aligned} \langle \Psi _N, \mathcal {N}^2 \Psi _N \rangle&\le C \langle e^{B^*-B} e^{A^*\Theta -\text {h.c.} }\Omega , (\mathcal {N}^2 + N^2_0) e^{B^*-B} e^{A^*\Theta -\text {h.c.} }\Omega \rangle \\&\le C \langle e^{A^*\Theta -\text {h.c.} }\Omega , (N^{\beta /2} \mathcal {N}^2 + N^2) e^{A^*\Theta -\text {h.c.} }\Omega \rangle \\&\le C N^{\beta /2} N^{2 - \beta } + C N^2 \le C N^2 \end{aligned} \end{aligned}$$

where in the last step we used the rough bound (97). □

## Energy of the Trial State

In this section, we are going to estimate the energy $\langle \psi _N, \mathcal {H}_N \psi _N \rangle$ of the trial state $\psi _N$ defined in (103). Together with Lemma 12, this will complete the proof of Theorem 2. Throughout the section, we will use the notation $\xi = e^{A^* \Theta - \Theta A} \Omega$ for the action of the cubic transformation on the vacuum, so that $\psi _N = W_{N_0} e^{B^* - B} \xi$.

### Action of Weyl and Bogoliubov Transformations on $\mathcal {H}_N$

In the next lemma, we compute the action of $W_{N_0}$ and of $e^{B^*-B}$ on the Hamiltonian

106 $$\begin{aligned} \mathcal {H}_N = \mathcal {K}+ \mathcal {V}_N = \sum _{p\in \Lambda ^*} p^2 a_p^* a_p + \frac{N^\kappa }{2N} \sum _{p,q,r\in \Lambda ^*} \hat{V}(r/N^{1-\kappa }) a_{p+r}^* a_{q-r}^* a_q a_p. \end{aligned}$$

This will allow us to express the energy $\langle \psi _N, \mathcal {H}_N \psi _N \rangle$ in terms of certain expectations in the state ξ, which will be estimated in the next subsections.

#### Lemma 13

We have107where

108 $$\begin{aligned} \begin{aligned} \mathcal {C}_N^*&= \frac{N^\kappa N_0^{1/2}}{N} \sum _{p, r\in \Lambda ^*} \hat{V}(r/N^{1-\kappa }) \sigma _p a_{p+r}^* a_{-r}^* a_{-p}^*, \\ \tilde{\mathcal {C}}_N^*&= \frac{N^\kappa N_0^{1/2}}{N} \sum _{p, r\in \Lambda ^*} \hat{V}(r/N^{1-\kappa }) ((\gamma _{p+r}\gamma _r-1)\sigma _p + \sigma _{p+r}\sigma _r\gamma _p) a_{p+r}^* a_{-r}^* a_{-p}^*, \\ \tilde{\mathcal {V}}_N&=\frac{N^\kappa }{2N} \sum _{p,q,r\in \Lambda ^*} \hat{V}(r/N^{1-\kappa }) (\gamma _{p+r} \gamma _{q-r}\gamma _{q}\gamma _{p}-1 +\sigma _{p+r}\sigma _{q-r}\sigma _{q}\sigma _{p} +2\gamma _{p+r}\sigma _{q-r}\sigma _{q}\gamma _p ) \\  &\quad \times a_{p+r}^* a_{q-r}^* a_{q}a_{p} \\&\quad +\frac{N^\kappa }{N} \sum _{p,q,r\in \Lambda ^*} \hat{V}(r/N^{1-\kappa }) \gamma _{p+r}\sigma _{q-r}\gamma _{q}\sigma _{p} a_{p+r}^*a_{-p}^* a_{-q+r} a_{q} \end{aligned} \end{aligned}$$

and $\mathcal {Q}$ is a quadratic operator satisfying

109 $$\begin{aligned} \pm \mathcal {Q}\le C N^\kappa (\mathcal {N}+ \tilde{\mathcal {N}}) \end{aligned}$$

with $$\tilde{\mathcal {N}} = \sum _{p\in \Lambda ^*} \sigma _p^2 a_p^* a_p$$.

*Remark:* in the next subsection, we will show that the expectations of the operators $\mathcal {Q}, \tilde{\mathcal {C}}_N, \tilde{\mathcal {V}}_N$ in the state $\xi = e^{A^* \Theta - \Theta A} \Omega$ are all negligible (they only contribute to order $N^{4\kappa -1}$ or lower).

#### Proof

We proceed as in [5, Section 3]. With (21), we find

110 $$\begin{aligned} \langle \Psi _N, \mathcal {H}_N \Psi _N \rangle = \sum _{n=0}^4 \langle e^{B^*-B} \xi , \mathcal {W}_n e^{B^*-B} \xi \rangle \end{aligned}$$

where

111 $$\begin{aligned} \begin{aligned} \mathcal {W}_0&= \frac{N^\kappa N_0^2}{2N} \hat{V}(0)\\ \mathcal {W}_1&= \frac{N^\kappa N_0^{3/2}}{N} \hat{V}(0) a_0^*+\text {h.c.} \\ \mathcal {W}_2&= \mathcal {K}+\frac{N^\kappa N_0}{2N} \sum _{p\in \Lambda ^*} \hat{V}(p/N^{1-\kappa }) \left( a_p^* a_{-p}^* + a_p a_{-p} + 2a_p^* a_{p} \right) \\&\quad +\frac{N^\kappa \hat{V}(0) N_0}{N} \sum _{p\in \Lambda ^*} a_p^* a_{p}\\ \mathcal {W}_3&= \frac{N^\kappa N_0^{1/2}}{N} \sum _{p,r \in \Lambda ^*} \hat{V}(r/N^{1-\kappa }) a_{p+r}^* a_{-r}^* a_p +\text {h.c.} \\ \mathcal {W}_4&= \mathcal {V}_N. \end{aligned} \end{aligned}$$

Setting $\mathcal {G}_n = e^{-B^* + B} \mathcal {W}_n e^{B^*-B}$ for n=0,⋯,4, we find

112 $$\begin{aligned} \langle \Psi _N, \mathcal {H}_N \Psi _N \rangle = \sum _{n=0}^4 \langle \xi , \mathcal {G}_n \xi \rangle . \end{aligned}$$

Clearly, we have $\langle \xi , \mathcal {G}_0 \xi \rangle = N^\kappa N_0^2 \hat{V}(0) / 2N$ and $\langle \xi , \mathcal {G}_1 \xi \rangle = 0$. Since $\langle \xi , a_p^* a_{-p}^* \xi \rangle = 0$ (because $A^*$ only creates triples), we obtain

113 $$\begin{aligned} \begin{aligned} \langle \xi , \mathcal {G}_2 \xi \rangle = \;&\langle \xi , \mathcal {K}\xi \rangle + \sum _{p\in \Lambda ^*} 2 p^2 \sigma _p^2 \langle \xi , a_p^* a_p \xi \rangle + \sum _{p\in \Lambda ^*} p^2 \sigma _p^2\\&\quad + \frac{N^\kappa N_0}{N} \sum _{p\in \Lambda ^*} \hat{V}(p/N^{1-\kappa }) (\gamma _p +\sigma _p)^2 \langle \xi , a_p^* a_{p}\xi \rangle \\  &\quad + \frac{N^\kappa N_0}{N} \sum _{p\in \Lambda ^*} \hat{V}(p/N^{1-\kappa }) (\gamma _p\sigma _p + \sigma _p^2)\\&\quad + \frac{N^\kappa \hat{V}(0) N_0}{N} \sum _{p\in \Lambda ^*} (\gamma _p^2 + \sigma _p^2)\langle \xi , a_p^* a_{p}\xi \rangle + \frac{N^\kappa \hat{V}(0) N_0}{N} \Vert \sigma \Vert _2^2 \,. \end{aligned} \end{aligned}$$

Taking into account that the expectation of operators having the form $a^* a^* a$ and $a^* a a$ in the state ξ is zero, we find

114 $$\begin{aligned} \langle \xi , \mathcal {G}_3 \xi \rangle= &   \frac{N^\kappa N_0^{1/2}}{N} \sum _{p, r\in \Lambda ^*} \hat{V}(r/N^{1-\kappa }) (\gamma _{p+r}\gamma _r\sigma _p\nonumber \\  &   + \sigma _{p+r}\sigma _r\gamma _p) \langle \xi , (a_{p+r}^* a_{-r}^* a_{-p}^* +\text {h.c.} )\xi \rangle . \end{aligned}$$

Finally, since operators of the form $a^* a^* a^* a^*$, $a^* a^* a^* a$, $a^* a a a$, *aaaa* have vanishing expectation in the state ξ, we obtain

115 $$\begin{aligned} \begin{aligned} \langle \xi , \mathcal {G}_4 \xi \rangle&= \frac{N^\kappa }{2N} \sum _{p,q,r\in \Lambda ^*} \hat{V}(r/N^{1-\kappa }) \big (\gamma _{p+r} \gamma _{q-r}\gamma _{q}\gamma _{p} +\sigma _{p+r}\sigma _{q-r}\sigma _{q}\sigma _{p} +2\gamma _{p+r}\sigma _{q-r}\sigma _{q}\gamma _p \big ) \\&\quad \times \langle \xi , a_{p+r}^* a_{q-r}^* a_{q} a_{p} \xi \rangle \\&\quad + \frac{N^\kappa }{N} \sum _{p,q,r\in \Lambda ^*} \hat{V}(r/N^{1-\kappa }) \gamma _{p+r}\sigma _{q-r}\gamma _{q}\sigma _{p} \langle \xi , a_{p+r}^*a_{-p}^* a_{-q+r} a_{q} \xi \rangle \\&\quad + \frac{N^\kappa }{N}\hat{V}(0) \Vert \sigma \Vert _2^2\sum _{p\in \Lambda ^*} (\gamma _{p}^2 +\sigma _p^2) \langle \xi , a_{p}^* a_{p} \xi \rangle \\&\quad + \frac{N^\kappa }{N} \sum _{p,r\in \Lambda ^*} \hat{V}(r/N^{1-\kappa }) \sigma _{p+r}^2 (\gamma _{p}^2 +\sigma _{p}^2) \langle \xi , a_{p}^* a_{p} \xi \rangle \\&\quad + \frac{2N^\kappa }{N} \sum _{p,r\in \Lambda ^*} \hat{V}(r/N^{1-\kappa }) \sigma _{p+r}\gamma _{p+r} \gamma _{p}\sigma _{p} \langle \xi , a_{p}^*a_{p} \xi \rangle \\&\quad + \frac{N^\kappa }{2N} \sum _{p,r\in \Lambda ^*} \hat{V}(r/N^{1-\kappa }) (\sigma _{p+r}\gamma _{p+r} \gamma _{p}\sigma _{p}+ \sigma _{p+r}^2\sigma _{p}^2) + \frac{N^\kappa }{2N} \hat{V}(0) \Vert \sigma \Vert _2^4. \end{aligned} \end{aligned}$$

Going through the r.h.s. of (113) and (115), we observe that all quadratic terms, with the exception of the kinetic energy operator $\mathcal {K}$, can be bounded as in (109) (here, we use the bounds from Lemma 5, which imply, in particular, that $p^2 \sigma _p^2 \le C N^\kappa$, for all $p \in \Lambda ^*$). The cubic terms in (114) correspond exactly to the sum of the two cubic terms defined in (108). Moreover, the quartic terms on the r.h.s. of (115) are clearly the sum of $\mathcal {V}_N$ and $\tilde{\mathcal {V}}_N$, with $\tilde{\mathcal {V}}_N$ as defined in (108). To complete the proof of Lemma 13, we only have to check that the constant terms arising from $\mathcal {G}_0, \mathcal {G}_2, \mathcal {G}_4$ match the r.h.s. of (107). To this end we notice, first of all, that the contribution $\langle \xi , \mathcal {G}_0 \xi \rangle$ can be combined with the last term on the r.h.s. of (113) and the last term on the r.h.s. of (115) to yield

$$\begin{aligned}  &   \frac{N^\kappa N_0^2}{2N} \hat{V} (0) + \frac{N^\kappa N_0}{N} \hat{V} (0) \Vert \sigma \Vert _2^2 + \frac{N^\kappa }{2N} \hat{V} (0) \Vert \sigma \Vert _2^4\\  &   \quad = \frac{N^\kappa }{2N} \hat{V} (0) \big (N_0 + \Vert \sigma \Vert _2^2 \big )^2 = \frac{N^{1+\kappa }}{2} \hat{V} (0). \end{aligned}$$

Next, we observe that the term

$$\begin{aligned} \Big | \frac{N^\kappa }{2N} \sum _{p,r} \hat{V} (r/ N^{1-\kappa }) \sigma _{p+r}^2 \sigma _p^2 \Big | \le C N^{\kappa -1} \Vert \hat{V} \Vert _\infty \Vert \sigma \Vert _2^4 \le C N^{4\kappa - 1} \end{aligned}$$

appearing on the r.h.s. of (115) is negligible. The remaining constant term on the r.h.s. of (115) can be rewritten as

$$\begin{aligned} \begin{aligned}&\frac{N^\kappa }{2N} \sum _{p,r} \hat{V} (r/N^{1-\kappa }) \sigma _{p+r} \gamma _{p+r}\gamma _p \sigma _p \\&\quad = \frac{N^\kappa }{2N} \sum _{p,r} \hat{V} (r/N^{1-\kappa }) \sigma _{\infty , p+r} \gamma _{\infty , p+r}\gamma _{\infty ,p} \sigma _{\infty ,p} + O (N^{4\kappa -1}) \end{aligned} \end{aligned}$$

because the contribution associated with low momenta $|p|, |p+r| \le N^{\kappa /2 - \beta /4}$ is negligible. Using the bound for $\gamma _{\infty ,p} \sigma _{\infty ,p} - \eta _{\infty ,p}$ in Lemma 3, we obtain

$$\begin{aligned} \begin{aligned}&\frac{N^\kappa }{2N} \sum _{p,r} \hat{V} (r/N^{1-\kappa }) \sigma _{p+r} \gamma _{p+r}\gamma _p \sigma _p \\&\quad = \frac{N^\kappa }{2N} \sum _{p,r} \hat{V} (r/N^{1-\kappa }) \eta _{\infty ,p+r} \eta _{\infty ,p} \\&\qquad + \frac{N^\kappa }{N} \sum _{p,r} \hat{V} (r/N^{1-\kappa }) \eta _{\infty , p+r} \big ( \gamma _{\infty ,p} \sigma _{\infty ,p} - \eta _{\infty ,p} \big ) + O (N^{4\kappa -1}).\end{aligned} \end{aligned}$$

As for the remaining constant terms in (113), we write

$$\begin{aligned} \begin{aligned}&\frac{N^\kappa N_0}{N} \sum _p \hat{V} (p/N^{1-\kappa }) \gamma _p \sigma _p \\&\quad = \frac{N^\kappa N_0}{N} \sum _p \hat{V} (p/N^{1-\kappa }) \eta _{\infty ,p}\\&\qquad + \frac{N^\kappa N_0}{N} \sum _p \hat{V} (p/N^{1-\kappa }) (\gamma _{\infty ,p} \sigma _{\infty ,p} - \eta _{\infty ,p}) + O (N^{4\kappa -1}) \\&\quad = N^\kappa \sum _p \hat{V} (p/N^{1-\kappa }) \eta _{\infty ,p} - \frac{N^\kappa \Vert \sigma \Vert _2^2}{N} \sum _p \hat{V} (p/N^{1-\kappa }) \eta _{\infty ,p} \\&\qquad + N^\kappa \sum _p \hat{V} (p/N^{1-\kappa }) (\gamma _{\infty ,p} \sigma _{\infty ,p} - \eta _{\infty ,p}) + O(N^{4\kappa -1}) \end{aligned} \end{aligned}$$

and, similarly,

$$\begin{aligned} \begin{aligned} \frac{N^\kappa N_0}{N}&\sum _p \hat{V} (p/N^{1-\kappa }) \sigma _p^2 = N^\kappa \sum _p \hat{V} (p/N^{1-\kappa }) \sigma _{\infty ,p}^2 + O (N^{4\kappa -1}) \end{aligned} \end{aligned}$$

as well as

$$\begin{aligned} \sum _p p^2 \sigma _p^2 = \sum _p p^2 \sigma _{\infty ,p}^2 + O (N^{4\kappa -1}). \end{aligned}$$

Putting together all constant contributions, we find

116 $$\begin{aligned}  &   \frac{N^{1+\kappa }}{2} \hat{V} (0) + \sum _{p\in \Lambda ^*} p^2 \sigma _{\infty ,p}^2 + N^\kappa \sum _{p\in \Lambda ^*} \hat{V}(p/N^{1-\kappa }) \eta _{\infty ,p}\nonumber \\  &   \qquad - \frac{N^\kappa \Vert \sigma \Vert _2^2}{N} \sum _{p\in \Lambda ^*} \hat{V}(p/N^{1-\kappa }) \eta _{\infty ,p}\nonumber \\  &   \qquad + N^\kappa \sum _{p\in \Lambda ^*} \hat{V}(p/N^{1-\kappa }) (\gamma _{\infty ,p}\sigma _{\infty ,p}-\eta _{\infty ,p}) + N^\kappa \sum _{p\in \Lambda ^*} \hat{V}(p/N^{1-\kappa }) \sigma _{\infty ,p}^2\nonumber \\  &   \qquad +\frac{N^\kappa }{2N} \sum _{p,r\in \Lambda ^*} \hat{V}(r/N^{1-\kappa }) \eta _{\infty ,p+r} \eta _{\infty ,p}\nonumber \\  &   \qquad +\frac{N^\kappa }{N} \sum _{p,r\in \Lambda ^*} \hat{V}(r/N^{1-\kappa }) \eta _{\infty ,p+r} (\gamma _{\infty ,p}\sigma _{\infty ,p}-\eta _{\infty ,p}) + O(N^{4\kappa -1}). \end{aligned}$$

With the scattering equation (27) and with (28), we combine

$$\begin{aligned} \begin{aligned} N^\kappa \sum _{p\in \Lambda ^*} \hat{V}(p/N^{1-\kappa }) \eta _{\infty ,p}&+\frac{N^\kappa }{2N} \sum _{p,r\in \Lambda ^*} \hat{V}(r/N^{1-\kappa }) \eta _{\infty ,p+r} \eta _{\infty ,p} \\ =&- \sum _{p \in \Lambda ^*} p^2 \eta _{\infty ,p}^2 + \frac{N^\kappa }{2} \sum _{p \in \Lambda ^*} \hat{V} (p/N^{1-\kappa }) \eta _{\infty ,p} + O(N^{2\kappa })\\ =&- \sum _{p \in \Lambda ^*} p^2 \eta _{\infty ,p}^2 + \frac{8\pi \mathfrak {a} - \hat{V} (0)}{2} N^{1+\kappa } + O(N^{2\kappa })\end{aligned} \end{aligned}$$

and, using also (33),

$$\begin{aligned} \begin{aligned}&N^\kappa \sum _{p\in \Lambda ^*} \hat{V}(p/N^{1-\kappa }) (\gamma _{\infty ,p}\sigma _{\infty ,p}-\eta _{\infty ,p})\\&\qquad +\frac{N^\kappa }{N} \sum _{p,r\in \Lambda ^*} \hat{V}(r/N^{1-\kappa }) \eta _{\infty ,p+r} (\gamma _{\infty ,p}\sigma _{\infty ,p}-\eta _{\infty ,p}) \\&\quad = -2 \sum _{p \in \Lambda ^*} p^2 \eta _{\infty ,p} ( \gamma _{\infty ,p}\sigma _{\infty ,p}-\eta _{\infty ,p}) + O(N^{4\kappa -1}). \end{aligned} \end{aligned}$$

Moreover, we write

$$\begin{aligned} \begin{aligned} N^\kappa \sum _{p\in \Lambda ^*} \hat{V} (p/N^{1-\kappa }) \sigma _{\infty ,p}^2 =&-2 \sum _{p\in \Lambda ^*} p^2 \eta _{\infty ,p} \sigma _{\infty ,p}^2 \\  &- N^{\kappa -1} \sum _{p,r \in \Lambda ^*} \hat{V} (r/N^{1-\kappa }) \eta _{\infty ,p+r} \sigma _{p}^2 + O(N^{4\kappa -1}).\end{aligned} \end{aligned}$$

Therefore, (116) can be rewritten as

$$\begin{aligned} \begin{aligned}&4\pi \mathfrak {a} N^{1+\kappa } + \sum _{p\in \Lambda ^*} p^2 (\sigma _{\infty ,p}^2 + \eta _{\infty ,p}^2 -2\eta _{\infty ,p} \gamma _{\infty ,p} \sigma _{\infty ,p} -2 \eta _{\infty ,p} \sigma _{\infty ,p}^2) \\&\quad - \frac{N^\kappa \Vert \sigma \Vert _2^2}{N} \sum _{p\in \Lambda ^*} \hat{V}(p/N^{1-\kappa }) \eta _{\infty ,p}\\&\quad - N^{\kappa -1} \sum _{p,r\in \Lambda ^*} \hat{V} (r/N^{1-\kappa }) \eta _{p+r} \sigma _{p}^2 + O(N^{4\kappa -1} + N^{2\kappa }) \end{aligned} \end{aligned}$$

and thus, inserting (26), as

$$\begin{aligned} \begin{aligned}&4\pi \mathfrak {a} N^{1+\kappa } + \frac{1}{2}\sum _{p\in \Lambda ^*} p^2 \left( \left( \frac{1-2\eta _{\infty ,p}}{\sqrt{1-4\eta _{\infty ,p}}}-1\right) (1-2\eta _{\infty ,p}) + 2\eta _{\infty ,p}^2 - 4 \frac{\eta _{\infty ,p}^2}{\sqrt{1-4\eta _{\infty ,p}}} \right) \\&\qquad - \frac{N^\kappa \Vert \sigma \Vert _2^2}{N} \sum _{p\in \Lambda ^*} \hat{V}(p/N^{1-\kappa }) \eta _{\infty ,p}\\&\qquad - N^{\kappa -1} \sum _{p,r\in \Lambda ^*} \hat{V} (r/N^{1-\kappa }) \eta _{p+r} \sigma _{p}^2 + O(N^{4\kappa -1} + N^{2\kappa }) \\&\quad = 4\pi \mathfrak {a} N^{1+\kappa } + \frac{1}{2}\sum _{p\in \Lambda ^*} p^2 \left( \sqrt{1-4\eta _{\infty ,p}} - 1 + 2\eta _{\infty ,p} + 2\eta _{\infty ,p}^2 \right) \\&\qquad - \frac{N^\kappa \Vert \sigma \Vert _2^2}{N} \sum _{p\in \Lambda ^*} \hat{V}(p/N^{1-\kappa }) \eta _{\infty ,p}\\&\qquad - N^{\kappa -1} \sum _{p,r\in \Lambda ^*} \hat{V} (r/N^{1-\kappa }) \eta _{p+r} \sigma _{p}^2 + O(N^{4\kappa -1} + N^{2\kappa }). \end{aligned} \end{aligned}$$

With the definition (24) of $\eta _{\infty ,p}$, we obtain exactly the constant term in (107). □

### Bounds for Negligible Cubic Expectations

In this subsection, we estimate the expectations $\langle \xi , \mathcal {Q}\xi \rangle , \langle \xi , ( \tilde{\mathcal {C}}_N + \tilde{\mathcal {C}}_N^* ) \xi \rangle , \langle \xi , \tilde{\mathcal {V}}_N \xi \rangle$, appearing on the r.h.s. of (107), and we show that they are at most of order $N^{4\kappa -1}$ and therefore negligible for our purposes. We will make use of the following preliminary lemma to bound expectations of cubic operators.

#### Lemma 14

Let

117 $$\begin{aligned} \mathcal {C}_K = \int dy_1 dy_2 dy_3 \, K (y_1-y_2,y_1-y_3) a_{y_1}a_{y_2}a_{y_3} + \text {h.c.} \end{aligned}$$

with $K \in L^2 (\mathbb {R}^3 \times \mathbb {R}^3)$. We assume that $K (y,z) = \sum _{u,v \in \Lambda _B} K_{u,v} (y,z)$, where $K_{u,v} (y,z) = 0$, if $|y-u| > 10 \ell _B$ or $|z-v| > 10 \ell _B$. Then, for every ϵ>0 and ν>0, we have

118 $$\begin{aligned} \begin{aligned} \big | \langle&e^{t A^*\Theta -\text {h.c.} } \Omega , \mathcal {C}_K e^{t A^*\Theta -\text {h.c.} }\Omega \rangle \big | \\&\le |\langle \Omega , [\mathcal {C}_K, A^* ] \Omega \rangle | \\  &\qquad + C \ell _B^3 N^{(3\kappa -1)/2+\epsilon } \big ( \sum _{u,v \in \Lambda _B} \Vert K_{u,v} \Vert _2 \big ) \int _0^t ds \langle e^{sA^*\Theta -\text {h.c.} }\Omega , \mathcal {N}e^{sA^*\Theta -\text {h.c.} }\Omega \rangle \\&\qquad + C N^{-\nu } \end{aligned} \end{aligned}$$

for all t∈[0;1], if we choose α>0 and then *n* large enough.

*Remark:* Notice that, in (118), we can bound

$$\begin{aligned} |\langle \Omega , [\mathcal {C}_K, A^* ] \Omega \rangle | \le C \Vert A \Vert _2 \Vert K \Vert _2 \le C N^{(3\kappa -1)/2} \Vert K \Vert _2. \end{aligned}$$

#### Proof

We take t=1 (the extension to t∈[0;1] is straightforward). With Duhamel, we obtain

119 $$\begin{aligned} \begin{aligned}&\big | \langle e^{A^*\Theta -\text {h.c.} }\Omega , \mathcal {C}_K e^{A^*\Theta -\text {h.c.} }\Omega \rangle \big | \\&\quad \le \int _0^1 ds \, \big | \langle e^{sA^*\Theta -\text {h.c.} }\Omega , [\mathcal {C}_K, A^*\Theta ] e^{sA^*\Theta -\text {h.c.} }\Omega \rangle \big | \\&\quad \le \int _0^1 ds \, \big | \langle e^{sA^*\Theta -\text {h.c.} } \Omega , ( \Theta [\mathcal {C}_K, A^*] \Theta +(1-\Theta )[\mathcal {C}_K, A^*]\Theta \\&\qquad + A^* \mathcal {C}_K (\Theta -1) - A^* (\Theta -1) \mathcal {C}_K ) e^{sA^*\Theta -\text {h.c.} }\Omega \rangle \big | \\&\quad \le \int _0^1 ds \, \big | \langle e^{sA^*\Theta -\text {h.c.} } \Omega , \Theta [\mathcal {C}_K, A^*] \Theta e^{sA^*\Theta -\text {h.c.} }\Omega \rangle \big | + C N^{-\nu } \end{aligned} \end{aligned}$$

if α>0 and then $n \in \mathbb {N}$ are chosen sufficiently large in (92). Here we estimated all terms containing a factor Θ-1 with Lemma 11, in particular (96), (97) and (98), using also the bound $\Vert A^* (\mathcal {N}+1)^{-3/2} \Vert \le C \Vert A \Vert _2, \Vert \mathcal {C}_K (\mathcal {N}+1)^{-3/2} \Vert \le C \Vert K \Vert _2$.

We observe that

120 $$\begin{aligned} \begin{aligned} [ \mathcal {C}_K, A^*]&= \int dx_1 dx_2 dx_3 dy_1 dy_2 dy_3 \, K (y_1 - y_2, y_1 - y_3) \\  &\quad \times \check{A} (x_1 - x_2, x_1 - x_3) \big [ a_{y_1} a_{y_2} a_{y_3}, a_{x_1}^* a_{x_2}^* a_{x_3}^* \big ] \end{aligned} \end{aligned}$$

where

121 $$\begin{aligned} \big [ a_{y_1} a_{y_2} a_{y_3}, a_{x_1}^* a_{x_2}^* a_{x_3}^* \big ]= &   \sum _{\pi \in S_3} \delta (y_1 - x_{\pi 1}) \delta (y_2 - x_{\pi 2}) \delta (y_3 - x_{\pi 3}) \nonumber \\  &   + \sum _{i_1 < i_2}^3 \sum _{\pi \in S_3} \delta (y_{i_1} - x_{\pi i_1}) \delta (y_{i_2} - x_{\pi i_2}) \prod _{i_3 \not = i_1, i_2} a_{x_{\pi i_3}}^* a_{y_{i_3}}\nonumber \\  &   + \sum _{i,j=1}^3 \delta (y_i - x_j) \prod _{\ell \not = j} a^*_{x_\ell } \prod _{m \not = i} a_{y_m}. \end{aligned}$$

We decompose the main term on the r.h.s. of (119) in the sum $T_4 + T_2 + T_0$, where $T_4$ are the contributions arising from the quartic terms on the r.h.s. of (121), $T_2$ from the quadratic terms and $T_0 = \langle \Omega , [\mathcal {C}_K, A^* ] \Omega \rangle$ from the constant terms. It is easy to check that $|T_0| \le C \Vert K \Vert _2 \Vert A \Vert _2$. Let us focus on the term $T_2$. As an example, we consider the contribution $T_{2,0}$ associated with $i_1 = \pi i_1 = 1, i_2 = \pi i_2 = 2$ in (121). Localizing the kernels $K, \check{A}$, we can bound

$$\begin{aligned}\begin{aligned} |T_{2,0}|&\le \sum _{u,v,\tilde{u}, \tilde{v} \in \Lambda _B} \int _0^1 ds \int dx_1 dx_2 dx_3 dy_3 \, |K_{u,v} (x_1 - x_2, x_1 - y_3)| |\check{A}_{\tilde{u}, \tilde{v}} (x_1 - x_2, x_1 - x_3)| \\&\quad \times \big | \langle \Theta e^{s A^* \Theta - \text {h.c.}} \Omega , a_{x_3}^* a_{y_3} \Theta e^{s A^* \Theta - \text {h.c.}} \Omega \rangle \big | \\&\le \sum _{u,v,\tilde{u}, \tilde{v} \in \Lambda _B} \int _0^1 ds \Big ( \int dx_1 dx_2 dx_3 dy_3 \, |K_{u,v} (x_1 - x_2, x_1 - y_3)|^2 \\  &\quad \times \mathbb {1}(|x_1 - x_2 - \tilde{u}|, |x_1 - x_3 - \tilde{v}| \le 10 \ell _B) \Vert a_{x_3} \Theta e^{sA^* \Theta - \text {h.c.} } \Omega \Vert ^2 \Big )^{1/2} \\  &\quad \times \Big ( \int dx_1 dx_2 dx_3 dy_3 \, |\check{A}_{\tilde{u},\tilde{v}} (x_1 - x_2, x_1 - x_3)|^2 \\&\quad \times \mathbb {1}(|x_1 - x_2 - u|, |x_1 - y_3 - v| \le 10 \ell _B) \Vert a_{y_3} \Theta e^{sA^* \Theta - \text {h.c.} } \Omega \Vert ^2 \Big )^{1/2}. \end{aligned} \end{aligned}$$

Using the characteristic functions to gain a volume factor $\ell _B^3$ (after switching to variables $x_2' = x_1 - x_2$, $y'_3= y_3 - x_1$, $x'_1 = x_1 - x_3$ and $x'_3 = x_3$ in the first integral on the r.h.s. and to variables $x'_2 = x_2 - x_1$, $x'_3 = x_3 - x_1$, $x'_1 = x_1 - y_3$, $y'_3 = y_3$ in the second) and applying Lemma 8, we can estimate

$$\begin{aligned} |T_{2,0}| \le C \ell _B^3 N^{(3\kappa -1)/2 + \epsilon } \sum _{u,v \in \Lambda _B} \Vert K_{u,v} \Vert _2 \int _0^1 ds \, \langle e^{sA^* \Theta - \text {h.c.} } \Omega , \mathcal {N}e^{sA^* \Theta - \text {h.c.} } \Omega \rangle . \end{aligned}$$

The other contributions to $T_2$ can be bounded similarly. Let us now consider $T_4$. Also here, we focus on an example, taking the term $T_{4,0}$ associated with i=j=1 on the third line of (121). We find

122 $$\begin{aligned} \begin{aligned} |T_{4,0}|&\le \sum _{u,v, \tilde{u}, \tilde{v}\in \Lambda _B} \int dx_1 dx_2 dx_3 dy_2 dy_3 | K_{u,v} (x_1-y_2,x_1-y_3) | \, | \check{A}_{\tilde{u}, \tilde{v}}(x_1-x_2,x_1-x_3)| \\&\quad \times \int _0^1 ds \, |\langle \Theta e^{sA^*\Theta -\text {h.c.} }\Omega , a^*_{x_2}a^*_{x_3} a_{y_{2}}a_{y_{3}} \Theta e^{sA^*\Theta -\text {h.c.} }\Omega \rangle | \\&\le \sum _{u,v, \tilde{u}, \tilde{v}\in \Lambda _B} \int _0^1 ds \Big ( \int dx_1 dx_2 dx_3 dy_2 dy_3 \, | K_{u,v} (x_1-y_2,x_1-y_3) |^2\\  &\quad \times \mathbb {1}(|x_1 - x_2 - \tilde{u}|, |x_1 - x_3 - \tilde{v}| \le 10 \ell _B) \Vert a_{x_2} a_{x_3} \Theta e^{sA^*\Theta -\text {h.c.} }\Omega \Vert ^2 \Big )^{1/2} \\  &\quad \times \Big ( \int dx_1 dx_2 dx_3 dy_2 dy_3 \, | \check{A}_{\tilde{u},\tilde{v}} (x_1-x_2,x_1-x_3) |^2 \\  &\quad \times \mathbb {1}(|x_1 - y_2 - u|, |x_1 - y_3 - v| \le 10 \ell _B) \Vert a_{y_2} a_{y_3} \Theta e^{sA^*\Theta -\text {h.c.} }\Omega \Vert ^2 \Big )^{1/2}.\end{aligned} \end{aligned}$$

We apply Lemma 11 to estimate, with the notation $\xi _s = e^{sA^* \Theta - \text {h.c.} } \Omega$,

$$\begin{aligned}  &   \int \mathbb {1}(|x_1 - \tilde{v} - x_3| \le 10 \ell _B) \Vert a_{x_3} a_{x_2} \Theta \xi _s \Vert ^2 dx_3\\  &   \quad = \Vert \mathcal {N}_{10 \ell _B}^{1/2} (x_1 - \tilde{v}) a_{x_2} \Theta \xi _s \Vert \le C N^\epsilon \Vert a_{x_2} \Theta \xi _s \Vert \\  &   \int \mathbb {1}(|x_1 - v - y_3| \le 10 \ell _B) \Vert a_{y_3} a_{y_2} \Theta \xi _s \Vert ^2 dy_3 \\  &   \quad = \Vert \mathcal {N}_{10 \ell _B}^{1/2} (x_1 - v) a_{y_2} \Theta \xi _s \Vert \le C N^\epsilon \Vert a_{y_2} \Theta \xi _s \Vert \end{aligned}$$

if *n* is large enough. With Lemma 8, we conclude that

$$\begin{aligned} |T_{4,0}| \le C \ell _B^3 N^{(3\kappa -1)/2+\epsilon } \sum _{u,v \in \Lambda _B} \Vert K_{u,v} \Vert _2 \int _0^1 ds \, \langle e^{sA^* \Theta - \text {h.c.} } \Omega , \mathcal {N}e^{sA^* \Theta - \text {h.c.} } \Omega \rangle . \end{aligned}$$

Also the other contributions to $T_4$ can be handled similarly. □

An immediate application of Lemma 14 is the following bound for the expectation of the number of excitations generated by the cubic transformation.

#### Lemma 15

We have

123 $$\begin{aligned} \langle e^{t A^*\Theta -\text {h.c.} }\Omega , \mathcal {N}e^{t A^*\Theta -\text {h.c.} }\Omega \rangle \le C N^{3\kappa -1} \end{aligned}$$

for all t∈[0;1], provided in (92) we choose α and then *n* large enough.

#### Proof

With $[\mathcal {N}, A^* ] = 3 A^*$, we find

$$\begin{aligned}  &   \langle e^{t A^*\Theta -\text {h.c.} }\Omega , \mathcal {N}e^{t A^*\Theta -\text {h.c.} }\Omega \rangle \\  &   \quad = 2 \text {Re }\int _0^t ds \langle e^{sA^*\Theta -\text {h.c.} }\Omega , (3A^* + 3A^*(\Theta -1)) e^{sA^*\Theta -\text {h.c.} }\Omega \rangle . \end{aligned}$$

The term proportional to Θ-1 can be handled with Lemma 11; it can be made arbitrarily small, in the sense that, for any $m\in \mathbb {N}$, we can estimate it by $C_m N^{-m}$ for a constant $C_m>0$ large enough, choosing α>0 and then $n \in \mathbb {N}$ sufficiently large in (92). To estimate the expectation of $A^*$, we apply Lemma 14, with K=A. From Lemma 7 and Lemma 8, we obtain

$$\begin{aligned} \begin{aligned} \langle e^{t A^*\Theta -\text {h.c.} }&\Omega , \mathcal {N}e^{t A^*\Theta -\text {h.c.} }\Omega \rangle \\&\le C N^{3\kappa -1} + C N^{- \beta /4 + \epsilon } \int _0^t ds \langle e^{sA^*\Theta -\text {h.c.} }\Omega , \mathcal {N}e^{sA^*\Theta -\text {h.c.} }\Omega \rangle \end{aligned} \end{aligned}$$

for any ϵ>0, if α,n are large enough. Choosing ϵ>0 small enough, we can apply Gronwall’s lemma to show (123). □

Inserting the bound (123) back into (118), we obtain the following corollary.

#### Corollary 16

Suppose the operator $\mathcal {C}_K$ is defined as in Lemma 14. For every ϵ>0, we have

124 $$\begin{aligned} \begin{aligned} \big |&\langle e^{t A^*\Theta -\text {h.c.} } \Omega , \mathcal {C}_K e^{t A^*\Theta -\text {h.c.} }\Omega \rangle \big | \le C N^{(3\kappa -1)/2} \Big ( \Vert K \Vert _2 + N^{-\beta /4 + \epsilon } \sum _{u,v \in \Lambda _B} \Vert K_{u,v} \Vert _2 \Big ) \end{aligned}\nonumber \\ \end{aligned}$$

for all t∈[0;1], provided α and then *n* are large enough.

We will also need bounds similar to the one in Lemma 14 for cubic operators with slightly more general kernels.

#### Corollary 17

For $f \in L^1 (\Lambda )$ and a kernel *K* as in Lemma 14, let

$$\begin{aligned} \mathcal {C}_{K,f} = \int dy dy_1 dy_2 dy_3 \, f (y) K (y+ y_1 - y_2, y+ y_1 - y_3) a_{y_1}^* a_{y_2}^* a_{y_3}^* + \text {h.c.} .\end{aligned}$$

Then we have, for any fixed ϵ>0,

$$\begin{aligned} \begin{aligned} \big | \langle e^{t A^*\Theta -\text {h.c.} } \Omega ,&\mathcal {C}_{K,f} e^{t A^*\Theta -\text {h.c.} }\Omega \rangle \big | \\  &\le |\langle \Omega , [\mathcal {C}_{K,f}, A^* ] \Omega \rangle | + C \Vert f \Vert _1 N^{(3\kappa -1)/2 - \beta /4 + \epsilon } \sum _{u,v \in \Lambda _B} \Vert K_{u,v} \Vert _2 \end{aligned} \end{aligned}$$

for all t∈[0;1], provided α and then *n* are large enough.

#### Proof

We proceed as in the proof of Lemma 14. To bound contributions to $[ \mathcal {C}_{K,f}, A^*]$ that are quadratic or quartic in creation and annihilation operators, we write $\mathcal {C}_{K,f}$ as average with weight *f* of operators that have exactly the form considered in Lemma 14. This observation allows us to derive the desired bound. □

The last two corollaries allow us to control the expectation $\langle \xi , (\tilde{\mathcal {C}}_N + \tilde{\mathcal {C}}_N^*)\xi \rangle$ appearing on the r.h.s. of (107).

#### Lemma 18

From (107) recall the definition

$$\begin{aligned} \tilde{\mathcal {C}}_N = N^{\kappa -1} N_0^{1/2} \sum _{p, r\in \Lambda ^*} \hat{V}(r/N^{1-\kappa }) ((\gamma _{p+r}\gamma _r-1)\sigma _p + \sigma _{p+r}\sigma _r\gamma _p) a_{p+r} a_{-r} a_{-p}. \end{aligned}$$

We have

125 $$\begin{aligned} \big | \langle e^{A^*\Theta -\text {h.c.} }\Omega , \tilde{\mathcal {C}}_N e^{A^*\Theta -\text {h.c.} }\Omega \rangle \big | \le C N^{4\kappa -1} \end{aligned}$$

for α and *n* chosen large enough.

#### Proof

We decompose $\tilde{\mathcal {C}}_N = \sum _{j=1}^4 \tilde{\mathcal {C}}^{(j)}_N$, where

$$\begin{aligned} \tilde{\mathcal {C}}^{(j)}_N = \sum _{p,r \in \Lambda ^*} K^{(j)}_{r,p} a_{p+r} a_{-r} a_{-p} \end{aligned}$$

with the coefficients

$$\begin{aligned} \begin{aligned} K^{(1)}_{r,p}&= N^{\kappa -1} N_0^{1/2} \hat{V} (r/N^{1-\kappa }) (\gamma _r - 1) \sigma _p \\ K^{(2)}_{r,p}&= N^{\kappa -1} N_0^{1/2} \hat{V} (r/N^{1-\kappa })\gamma _r (\gamma _{p+r}-1) \sigma _p \\ K^{(3)}_{r,p}&= N^{\kappa -1} N_0^{1/2} \hat{V} (r/N^{1-\kappa }) \sigma _{p+r} \sigma _r \\ K^{(4)}_{r,p}&= N^{\kappa -1} N_0^{1/2} \hat{V} (r/N^{1-\kappa }) \sigma _{p+r} \sigma _r (\gamma _p -1). \end{aligned} \end{aligned}$$

With the momentum space bounds from Lemma 5, we get $\Vert K^{(j)} \Vert _2 \le C N^{(5\kappa -1)/2}$, for all j=1,⋯,4. Switching to position space, we write, for j=1,⋯,4,

$$\begin{aligned} \tilde{\mathcal {C}}^{(j)}_N = \int dx dy dz \, \check{K}^{(j)} (x-y, x-z) a_x a_y a_z \end{aligned}$$

withFor $u \in \Lambda _B= \Lambda \cap \ell _B \mathbb {Z}^3$, we recall $\check{\sigma }_u = \check{\sigma }\mathbb {1}_{B_u}$ and , with $B_u = \{x\in \Lambda , \inf _{v\in \Lambda _B} |x-v | =|x-u | \}$. For $u,v \in \Lambda _B$, we define the localized kerneland we notice that . With Lemma 6 we find

$$\begin{aligned} \sum _{u,v \in \Lambda _B} \Vert K^{(1)}_{u,v} \Vert _2 \le C N^{(5\kappa -1)/2 + \epsilon } \end{aligned}$$

for any ϵ>0, if α and then *n* are large enough. Recalling that $\Vert K^{(1)} \Vert _2 \le C N^{(5\kappa -1)/2}$, we obtain from Corollary 16 that

$$\begin{aligned} \big | \langle e^{A^*\Theta -\text {h.c.} }\Omega , \tilde{\mathcal {C}}^{(1)}_N e^{A^*\Theta -\text {h.c.} }\Omega \rangle \big | \le C N^{4\kappa -1} (1+ N^{ -\beta /4+\epsilon }) \le C N^{4\kappa -1}\end{aligned}$$

if ϵ>0 is chosen small enough (and α and then *n* are large enough). The expectation of $\tilde{\mathcal {C}}^{(3)}_N$ can be bounded analogously. To control the contribution of $\tilde{\mathcal {C}}^{(2)}_N$, we observe that

$$\begin{aligned} \check{K}^{(2)} (y,z) = \int dw \, f (w) \tilde{K}^{(2)} (w + y, w+z) \end{aligned}$$

with $f (w) = [N^{3-3\kappa } V (N^{1-\kappa } \cdot ) * \check{\gamma }] (w)$ and . From Lemma 6, we have

$$\begin{aligned} \sum _{u,v \in \Lambda _B} \Vert \tilde{K}^{(2)}_{u,v} \Vert _2 \le N^{5\kappa /2-1/2+\epsilon }.\end{aligned}$$

Since moreover

$$\begin{aligned} | \langle \Omega , [ \tilde{\mathcal {C}}_N^{(2)}, A^* ] \Omega \rangle | \le C \Vert K^{(2)} \Vert _2 \Vert A \Vert _2 \le C N^{4\kappa -1} \end{aligned}$$

and $\Vert f \Vert _1 \le C N^{\beta /8+\epsilon }$ (with the bounds in Lemma 5, we find , for any ϵ>0), Corollary 17 implies that

$$\begin{aligned} \big | \langle e^{A^*\Theta -\text {h.c.} }\Omega , \tilde{\mathcal {C}}^{(2)}_N e^{A^*\Theta -\text {h.c.} }\Omega \rangle \big | \le C N^{4\kappa -1} (1 + N^{-\beta /8 + \epsilon } ) \le C N^{4\kappa -1} \end{aligned}$$

if ϵ>0 is chosen sufficiently small. The expectation of $\tilde{\mathcal {C}}^{(4)}_N$ can be handled analogously. □

In the next lemma, we show a bound for the expectation of the operator $\tilde{\mathcal {N}} = \sum _{p \in \Lambda ^*} \sigma _p^2 a_p^* a_p$. Since it will be useful in the next sections, we actually prove a more general estimate, controlling the expectation of general non-negative quadratic operators, diagonal in momentum space. For $k \in L^1 (\Lambda )$, we introduce the notation

$$\begin{aligned} \mathcal {N}_k = \int dz dz' \, k (z-z') a_z^* a_{z'} \,.\end{aligned}$$

#### Lemma 19

Let $h \in L^2 (\Lambda )$, real-valued. We assume that $h (x) = \sum _{u \in \Lambda _B} h_u (x)$, where $h_u \in L^2 (\Lambda )$ for all $u \in \Lambda _B$, with $h_u (x) = 0$, if $|x-u| > 10 \ell _B$. Then, for every ϵ>0 there is a constant C>0 such that

126 $$\begin{aligned} \begin{aligned} \langle e^{t A^* \Theta - \text {h.c.} } \Omega ,&\mathcal {N}_{h \star h} e^{t A^* \Theta - \text {h.c.} } \Omega \rangle \\  &\le C \langle \Omega , \big [ A, \big [ \mathcal {N}_{h\star h}, A^* \big ] \big ] \Omega \rangle + C N^{-5\beta /8 + \epsilon } \Big (\sum _{u \in \Lambda _B} \Vert h_u \Vert _2 \Big )^2 \end{aligned} \end{aligned}$$

for all t∈[0;1], if α and then $n \in \mathbb {N}$ are large enough. Here, we used the notation

$$\begin{aligned} h \star h (x) = \int h (y-x) h (y) dy \end{aligned}$$

(h⋆h is the usual convolution, if h(-y)=h(y)). In particular, recalling that $\tilde{\mathcal {N}} = \mathcal {N}_{\check{\sigma } \star \check{\sigma }}$, we find C>0 such that, for all t∈[0;1],

$$\begin{aligned} \langle e^{t A^* \Theta - \text {h.c.} } \Omega , \tilde{\mathcal {N}} e^{t A^* \Theta - \text {h.c.} } \Omega \rangle \le C N^{3\kappa -1}.\end{aligned}$$

*Remark.* For $k \in L^1 (\Lambda )$, we also define the operator

127 $$\begin{aligned} \ddot{\mathcal {N}}_k = \int dz dz' \, k(z-z') [a_{z'}, A^* ] [ a_z^*, -A] \end{aligned}$$

contributing to the second derivative of $\mathcal {N}_k$ (hence the notation). From the proof of Lemma 19, we also obtain

$$\begin{aligned} \big | \langle e^{s A^* \Theta -\text {h.c.} } \Omega , \ddot{\mathcal {N}}_{h \star h} e^{s A^* \Theta - \text {h.c.} } \Omega \rangle \big | \le C N^{-5\beta /8 + \epsilon } \big ( \sum _{u \in \Lambda _B} \Vert h_u \Vert \big )^2 \end{aligned}$$

for all s∈[0;1] and for any ϵ>0, if α and then *n* are chosen large enough in the definition of the cutoff Θ. This bound will be used in the next subsection.

#### Proof

Throughout the proof, we will use the notation g=h⋆h. By Cauchy–Schwarz, we have

$$\begin{aligned} \Vert h \Vert _1 = \int |h (x)| dx \le \sum _{u \in \Lambda _B} \int |h_{u} (x)| dx \le C \ell _B^{3/2} \sum _{u \in \Lambda _B} \Vert h_{u} \Vert _2 \end{aligned}$$

and thus

$$\begin{aligned} \begin{aligned} \Vert \hat{h} \Vert _\infty \le C \ell _B^{3/2} \sum _{u \in \Lambda _B} \Vert h_{u} \Vert _2, \qquad \Vert \hat{g} \Vert _\infty \le \Vert g \Vert _1 \le C \ell _B^{3} \big (\sum _{u \in \Lambda _B} \Vert h_{u} \Vert _2 \big )^2.\end{aligned} \end{aligned}$$

Setting $\xi _t = e^{t A^* \Theta - \text {h.c.} } \Omega$ for t∈[0;1], we find

$$\begin{aligned} \begin{aligned}&\langle \xi _t, \mathcal {N}_g \xi _t \rangle = 2 \text {Re } \int _0^t ds \langle \xi _s, \big ( [\mathcal {N}_g, A^*] +[\mathcal {N}_g, A^*](\Theta -1) +A^* [\mathcal {N}_g,(\Theta -1)] \big ) \xi _s \rangle . \end{aligned} \end{aligned}$$

All terms containing a factor (Θ-1) can be handled with Lemma 11, in particular with (96), (97), (98). Since $\mathcal {N}_g \le \Vert \hat{g} \Vert _\infty \mathcal {N}$, $\Vert A (\mathcal {N}+1)^{-3/2}\Vert \le C N^{(3\kappa -1)/2}$, it is easy to check that they can be included in the error proportional to $(\sum _u \Vert h_u \Vert _2)^2$ on the r.h.s. of (126), if we choose α>0 and then $n \in \mathbb {N}$ large enough in (92). Also in the rest of the proof we will ignore these terms. With the definition (76), we compute

128 $$\begin{aligned} [\mathcal {N}_g, A^*] = \int dzdz'dx_1dx_2 \, g(z-z')\check{A}^{(3)} (x_1-x_2,x_1-z')a^*_za^*_{x_1}a^*_{x_2}. \end{aligned}$$

Expanding once more, we arrive at

$$\begin{aligned} \langle \xi _t, \mathcal {N}_g \xi _t \rangle \le 2 \text {Re } \int _0^t ds_1 \int _0^{s_1} ds_2 \, \langle \xi _{s_2}, [[ \mathcal {N}_g, A^* ], -A ] \xi _{s_2} \rangle + C N^{-5\beta /8 + \epsilon } \big (\sum _{u \in \Lambda _B} \Vert h_u \Vert _2 \big )^2 \end{aligned}$$

where

129 $$\begin{aligned} [[\mathcal {N}_g, A^*],-A]=\langle \Omega , [A,[\mathcal {N}_g,A^*]]\Omega \rangle +T_2+T_{4,1}+T_{4,2} \end{aligned}$$

with

$$\begin{aligned} \begin{aligned} T_{2}&=-\int dzdz'dx_1dx_2dx_3 \, g(z-z')\check{A}^{(6)} (x_1-x_2,x_1-z')\check{A}^{(6)} (x_3-x_2,x_3-z)a^*_{x_1}a_{x_3}\\  &\hspace{0.4cm} + \int dzdz'dx_1dx_2dx_3 \, g(z-z')\check{A}^{(3)} (x_1-x_2,x_1-z')\check{A}^{(6)} (x_3-x_2,x_3-x_1)a^*_{z}a_{x_3} \end{aligned}\end{aligned}$$

and

$$\begin{aligned} \begin{aligned} T_{4,1}&=\int dzdz'dx_1dx_2dx_3dx_4 \, g(z-z') \\  &\quad \times \check{A}^{(6)} (x_1-x_2,x_1-z')\check{A}^{(3)} (x_3-x_4,x_3-x_1)a^*_{z}a^*_{x_2}a_{x_3}a_{x_4}\\T_{4,2}&=\int dzdz'dx_1dx_2dx_3dx_4 \, g(z-z') \\  &\quad \times \check{A}^{(3)} (x_1-x_2,x_1-z')\check{A}^{(3)} (x_3-x_4,x_3-z)a^*_{x_1}a^*_{x_2}a_{x_3}a_{x_4} \end{aligned} \end{aligned}$$

where we recall the kernels $\check{A}^{(3)}, \check{A}^{(6)}$ defined in (76). Using (79), we pass to momentum space, writing

$$\begin{aligned} T_2 = \sum _{p,q \in \Lambda ^*} \hat{g}_p \, A^{(6)}_{q,p} \, A^{(3)}_{q,p} \big ( a_p^* a_p + a_q^* a_q + a_{-p-q}^* a_{-p-q} \big ). \end{aligned}$$

With (79) and with Lemma 9, we estimate

$$\begin{aligned}\begin{aligned} \big | \langle \xi _{s_2}, T_2 \xi _{s_2} \rangle \big |&\le C N^{3\kappa /2-1} \Vert \hat{g} \Vert _\infty \langle \xi _{s_2}, (\mathcal {N}+ \tilde{\mathcal {N}}) \xi _{s_2} \rangle \\  &\le C N^{\beta /4-1} \big (\sum _{u \in \Lambda _B} \Vert h_u \Vert _2 \big )^2 \langle \xi _{s_2}, (\mathcal {N}+ \tilde{\mathcal {N}}) \xi _{s_2} \rangle .\end{aligned} \end{aligned}$$

We now consider the term $T_{4,1}$. Using Lemma 11 to insert the cutoff Θ (choosing α>0 and $n \in \mathbb {N}$ large enough), it is enough to estimate

$$\begin{aligned} \begin{aligned}&|\langle \xi _{s_2}, \Theta T_{4,1} \Theta \xi _{s_2} \rangle |\\&\quad \le \int dy dz' dx_1 dx_2 dx_3 dx_4 |h (y-z') | |\check{A}^{(6)} (x_1-x_2,x_1-z') | |\check{A}^{(3)} (x_3-x_4,x_3-x_1) | \\&\qquad \times \Big | \int dz\, h (y-z)\langle \Theta \xi _{s_2}, a^*_z a_{x_2}^* a_{x_3} a_{x_4} \Theta \xi _{s_2} \rangle \Big |. \end{aligned} \end{aligned}$$

Next, we localize *h* and the kernels $\check{A}^{(3)}, \check{A}^{(6)}$. With Cauchy–Schwarz, we obtain

$$\begin{aligned} \begin{aligned}&|\langle \xi _{s_2}, \Theta T_{4,1}\Theta \xi _{s_2} \rangle |\\&\quad \le \sum _{u,v,\tilde{u}, \tilde{v}, w \in \Lambda _B} \Big (\int dy dz' dx_1 dx_2 dx_3 dx_4 \, |h_{w} (y-z')|^2 |\check{A}^{(3)}_{\tilde{u},\tilde{v}} (x_3-x_4,x_3-x_1) |^2 \\&\qquad \times \mathbb {1}(|x_1-x_2-u |, |x_1-z'-v |\le 10 \ell _B)\Big \Vert \int dz\, h (y-z) a_{x_2} a_z \Theta \xi _{s_2} \Big \Vert ^2\Big )^{1/2} \\  &\qquad \times \Big (\int dy dz' dx_1 dx_2 dx_3 dx_4 |\check{A}^{(6)}_{u,v}(x_1-x_2,x_1-z') |^2 \\&\qquad \times \mathbb {1}(|x_3-x_4-\tilde{u} |,|x_3-x_1-\tilde{v} |,|y-z'-w | \le 10 \ell _B) \Vert a_{x_3} a_{x_4} \Theta \xi _{s_2} \Vert ^2\Big )^{1/2}.\end{aligned}\end{aligned}$$

Applying Lemma 11 and then Lemma 9 we find, for any ϵ>0,

$$\begin{aligned} \begin{aligned} |\langle \xi _{s_2},&\Theta T_{4,1} \Theta \xi _{s_2} \rangle | \\  &\le C \ell _B^{9/2} N^{\epsilon /2} \big (\sum _{ w\in \Lambda _B} \Vert h_{w} \Vert _2\big ) \big (\sum _{u,v\in \Lambda _B} \Vert \check{A}^{(6)}_{u,v} \Vert _2\big )\big (\sum _{\tilde{u},\tilde{v}\in \Lambda _B} \Vert \check{A}^{(3)}_{\tilde{u},\tilde{v}} \Vert _2\big ) \\  &\quad \times \Vert \mathcal {N}^{1/2} \Theta \xi _{s_2} \Vert \Big ( \int dy\; \Big \Vert \int dz\, h (y-z) a_z \Theta \xi _{s_2} \Big \Vert ^2 \Big )^{1/2} \\&\le C N^{\beta /4-1+\epsilon } \big ( \sum _u \Vert h_u \Vert _2 \big )^2 \langle \xi _{s_2}, \mathcal {N}\xi _{s_2} \rangle + C \langle \xi _{s_2}, \mathcal {N}_g \xi _{s_2} \rangle + C N^{-5\beta /8+\epsilon } \big ( \sum _u \Vert h_u \Vert _2 \big )^2 \end{aligned} \end{aligned}$$

if the parameters α>0 and *n* in the definition of Θ are chosen sufficiently large.

To bound $T_{4,2}$, we expand it once again, estimating

$$\begin{aligned} \begin{aligned} \text {Re }&\int _0^t ds_1 \int _0^{s_1} ds_2 \, \langle \xi _{s_2}, T_{4,2} \xi _{s_2} \rangle \\  &\le \text {Re } \int _0^t ds_1 \int _0^{s_1} ds_2 \int _0^{s_2} ds_3 \, \langle \xi _{s_3}, \Theta [T_{4,2}, A^* ] \Theta \xi _{s_3} \rangle + C N^{-5\beta /8+\epsilon } \big ( \sum _u \Vert h_u \Vert _2 \big )^2 \end{aligned} \end{aligned}$$

for α>0 and $n\in \mathbb {N}$ large enough (using again Lemma 11 to insert Θ). We compute

$$\begin{aligned} [T_{4,2},A^*]= T_{4,2,3} + T_{4,2,5} \end{aligned}$$

with

$$\begin{aligned} \begin{aligned} T_{4,2,3}&= \int dzdz'dx_1dx_2dx_3dx_4dx_5 \, g(z-z')\check{A}^{(3)} (x_1-x_2,x_1-z')\\  &\quad \times \check{A}^{(3)} (x_3-x_4,x_3-z)\check{A}^{(6)} (x_5-x_3,x_5-x_4)a^*_{x_1}a^*_{x_2}a^*_{x_5}\\ T_{4,2,5}&= \int dzdz'dx_1dx_2dx_3dx_4dx_5dx_6 \, g(z-z')\check{A}^{(3)} (x_1-x_2,x_1-z') \\  &\quad \times \check{A}^{(6)} (x_3-x_4,x_3-z)A^{(3)} (x_5-x_6,x_5-x_4)a^*_{x_1}a^*_{x_2}a^*_{x_5}a^*_{x_6}a_{x_3}. \end{aligned} \end{aligned}$$

For the quintic term, we find

130 $$\begin{aligned} \begin{aligned} |\langle&\xi _{s_3}, \Theta T_{4,2,5} \Theta \xi _{s_3} \rangle |\\  &\le \int dz dz' dx_1 dx_2 dx_3 dx_4 dx_5 dx_6 \, |g(z-z') | |\check{A}^{(3)} (x_1-x_2,x_1-z') | \\&\hspace{.4cm} \times |\check{A}^{(6)} (x_3-x_4,x_3-z) | |\check{A}^{(3)} (x_5-x_6,x_5-x_4) | \Vert a_{x_{1}} a_{x_{2}} a_{x_{5}}a_{x_{6}}\Theta \xi _{s_3} \Vert \Vert a_{x_3}\Theta \xi _{s_3} \Vert . \end{aligned} \end{aligned}$$

Localizing the kernels $\check{A}^{(3)}, \check{A}^{(6)}$ and applying Cauchy–Schwarz, Lemma 11 and Lemma 9, we obtain

$$\begin{aligned} \begin{aligned} |\langle&\xi _{s_3}, \Theta T_{4,2,5} \Theta \xi _{s_3} \rangle |\\  &\le \sum _{u_i,v_i \in \Lambda _B: \, i=1,2,3} \Big (\int dzdz'dx_1 dx_2 dx_3 dx_4 dx_5 dx_6 \, |g(z-z') ||\check{A}^{(6)}_{u_2,v_2}(x_3-x_4,x_3-z) |^2 \\  &\hspace{.5cm} \times \mathbb {1}(|x_1-x_2-u_1|,|x_1-z'-v_1|,|x_5-x_6-u_3|,|x_5-x_4-v_3|\le 10\ell _B)\\  &\hspace{.5cm}\times \Vert a_{x_{1}} a_{x_{2}} a_{x_{5}}a_{x_{6}}\Theta \xi _{s_3} \Vert ^2\Big )^{1/2}\\&\qquad \times \Big (\int dzdz'dx_1 dx_2 dx_3 dx_4 dx_5 dx_6 \, |g(z-z') | |\check{A}^{(3)}_{u_1,v_1}(x_1-x_2,x_1-z') |^2 \\&\hspace{.5cm} \times |\check{A}^{(3)}_{u_3,v_3}(x_5-x_6,x_5-x_4) |^2 \mathbb {1}(|x_3-x_4-u_2|,|x_3-z-v_2|\le 10\ell _B) \Vert a_{x_{3}}\Theta \xi _{s_3} \Vert ^2\Big )^{1/2}\\&\le C N^{\epsilon } \Big (\sum _{u,v\in \Lambda _B} \ell _B^{3/2}\Vert \check{A}^{(3)}_{u,v} \Vert \Big )^{2}\Big (\sum _{u,v\in \Lambda _B} \ell _B^{3/2}\Vert \check{A}^{(6)}_{u,v} \Vert \Big ) \Vert g \Vert _1 \langle \Theta \xi _{s_3}, \mathcal {N}\Theta \xi _{s_3} \rangle \\&\le C N^{3\beta /8 -1 + \epsilon } \big ( \sum _{u \in \Lambda _B} \Vert h_u \Vert \big )^2 \, \langle \xi _{s_3}, \mathcal {N}\xi _{s_3} \rangle + C N^{-5\beta /8+\epsilon } \big ( \sum _u \Vert h_u \Vert _2 \big )^2 \end{aligned}\end{aligned}$$

for $n \in \mathbb {N}$ large enough. As for the cubic terms $T_{4,2,3}$, we need to expand it a last time, writing

$$\begin{aligned}\begin{aligned}&\text {Re } \int _0^1 ds_1 \int _0^{s_1} ds_2 \int _0^{s_2} d{s_3} \, \langle \xi _{s_3}, T_{4,2,3} \xi _{s_3} \rangle \\&\quad \le \text {Re } \int _0^1 ds_1 \int _0^{s_1} ds_2 \int _0^{s_2} d{s_3} \int _0^{s_3} ds_4 \, \langle \xi _{s_4}, \Theta [ T_{4,2,3}, A^* ] \Theta \xi _{s_4} \rangle \\&\qquad + C N^{-5\beta /8+\epsilon } \big ( \sum _u \Vert h_u \Vert _2 \big )^2. \end{aligned} \end{aligned}$$

A long but straightforward computation shows that

$$\begin{aligned} [T_{4,2,3},A^*]= T_{4,2,3,0} + T_{4,2,3,2}+T_{4,2,3,4} \end{aligned}$$

with

$$\begin{aligned} T_{4,2,3,0}= &   \int dzdz'dx_1 \dots dx_5\;g(z-z')\check{A}^{(3)} (x_1-x_2,x_1-z')\\    &   \times \check{A}^{(3)} (x_3-x_4,x_3-z)\check{A}^{(6)} (x_5-x_3,x_5-x_4)\check{A}^{(6)} (x_1-x_2,x_1-x_5)\\ T_{4,2,3,2}= &   \int dzdz'dx_1 \dots dx_6 \;g(z-z')\check{A}^{(3)} (x_1-x_2,x_1-z')\check{A}^{(3)} (x_3-x_4,x_3-z) \\    &   \times \check{A}^{(6)} (x_5-x_3,x_5-x_4)\check{A}^{(6)} (x_6-x_1,x_6-x_2)a^*_{x_5}a_{x_6}\\    &   +\int dzdz'dx_1 \dots dx_6 \;g(z-z')\check{A}^{(6)} (x_1-x_2,x_1-z')\check{A}^{(3)} (x_3-x_4,x_3-z) \\    &   \times \check{A}^{(6)} (x_5-x_3,x_5-x_4)\check{A}^{(6)} (x_6-x_5,x_6-x_2)a^*_{x_1}a_{x_6}\\ T_{4,2,3,4}= &   \int dzdz'dx_1 \dots dx_7\;g(z-z')\check{A}^{(6)} (x_1-x_2,x_1-z')\check{A}^{(3)} (x_3-x_4,x_3-z) \\    &   \times \check{A}^{(6)} (x_5-x_3,x_5-x_4)\check{A}^{(3)} (x_6-x_7,x_6-x_1)a^*_{x_5}a^*_{x_2}a_{x_6}a_{x_7}\\    &   +\int dzdz'dx_1 \dots dx_7\;g(z-z')\check{A}^{(3)} (x_1-x_2,x_1-z') \check{A}^{(3)} (x_3-x_4,x_3-z) \\    &   \times \check{A}^{(6)} (x_5-x_3,x_5-x_4)\check{A}^{(3)} (x_6-x_7,x_6-x_5)a^*_{x_1}a^*_{x_2}a_{x_6}a_{x_7}. \end{aligned}$$

For $T_{4,2,3,0}$ and $T_{4,2,3,2}$ we switch to momentum space. With (76) and Lemma 9, we obtain

$$\begin{aligned} \begin{aligned} |T_{4,2,3,0} |&\le \sum _{p,q,r \in \Lambda ^*} |\hat{g}_p| |A^{(6)}_{r,p} ||A^{(3)}_{r,p} ||A^{(6)}_{q,p} ||A^{(3)}_{q,p} | \\  &\le C \Vert \hat{g} \Vert _\infty \sum _{p,q,r \in \Lambda ^*} |A^{(6)}_{r,p} |^2 |A^{(3)}_{q,p}|^2 \\&\le C N^{-\beta /2} \Vert \hat{g}\Vert _\infty \sum _{p,r \in \Lambda ^*}(1+\sigma _p^2)|A^{(6)}_{r,p} |^2 \le C N^{-3\beta /4+\epsilon } \big (\sum _u \Vert h_u \Vert _2 \big )^2 \end{aligned} \end{aligned}$$

for any ϵ>0. Similarly,

$$\begin{aligned} \begin{aligned} | \langle \xi _{s_4}&, T_{4,2,3,2} \xi _{s_4} \rangle | \\&\le C \sum _{p,q,r \in \Lambda ^*} |\hat{g}_p| |A^{(6)}_{r,p} ||A^{(3)}_{r,p} ||A^{(6)}_{q,p} ||A^{(3)}_{q,p} | \langle \xi _{s_4}, (a_{p}^* a_{p} + a_{q}^* a_{q} + a_{p+q}^* a_{p+q}) \xi _{s_4} \rangle \\&\le C N^{-\beta /2} \Vert \hat{g} \Vert _\infty (1+ \Vert \sigma \Vert _\infty ^2) \sum _{j=3,6} \sum _{p,q \in \Lambda ^*} |A^{(j)}_{q,p} |^2 \langle \xi _{s_4}, (a_{p}^* a_{p} + a_{q}^* a_{q} + a_{p+q}^* a_{p+q}) \xi _{s_4} \rangle \\&\le C N^{-1+\epsilon } \big ( \sum _u \Vert h_u \Vert _2 \big )^2 \langle \xi _{s_4}, (\mathcal {N}+ \tilde{\mathcal {N}}) \xi _{s_4} \rangle . \end{aligned} \end{aligned}$$

As for the quartic contribution, we find that, for any ϵ>0,

$$\begin{aligned} \begin{aligned}&| \langle \Theta \xi _{s_4}, T_{4,2,3,4} \Theta \xi _{s_4} \rangle |\\&\quad \le C N^{\epsilon } \Big (\big (\sum _{u,v \in \Lambda _B} \ell _B^{3/2}\Vert \check{A}^{(3)}_{u,v} \Vert \big )^{4} + \big (\sum _{u,v \in \Lambda _B} \ell _B^{3/2}\Vert \check{A}^{(6)}_{u,v} \Vert \big )^{4} \Big ) \Vert g \Vert _1 \langle \xi _{s_4}, \mathcal {N}\xi _{s_4} \rangle \\&\quad \le C N^{\beta /4 -1 + \epsilon }\big ( \sum _u \Vert h_u \Vert _2 \big )^2 \langle \xi _{s_4}, \mathcal {N}\xi _{s_4}\rangle , \end{aligned} \end{aligned}$$

if $n \in \mathbb {N}$ in the definition of Θ is large enough. This bound can be shown proceeding similarly as we did for $T_{4,2,5}$ in (130); we omit the details.

Combining all the bounds we proved, we obtain

$$\begin{aligned} \begin{aligned} \langle \xi _t, \mathcal {N}_g \, \xi _t \rangle&\le \; \text {Re } \langle \Omega , [ A, [ \mathcal {N}_g, A^* ]] \Omega \rangle \\&\quad + C N^{3\beta /8 -1 + \epsilon } \big ( \sum _{u \in \Lambda _B} \Vert h_u \Vert \big )^2 \, \int _0^t ds \langle \xi _s, (\mathcal {N}+ \tilde{\mathcal {N}}) \xi _s \rangle \\&\quad + C \int _0^t ds \langle \xi _s, \mathcal {N}_g \xi _s \rangle + C N^{-5\beta /8+\epsilon } \big (\sum _{u \in \Lambda _B} \Vert h_u \Vert _2 \big )^2 \end{aligned} \end{aligned}$$

for all t∈[0;1]. With Lemma 15, we arrive at

131 $$\begin{aligned} \begin{aligned} \langle \xi _t, \mathcal {N}_g \xi _t \rangle \le \;&\text {Re } \langle \Omega , [ A, [ \mathcal {N}_g, A^* ]] \Omega \rangle + C N^{3\beta /8 -1 + \epsilon } \big ( \sum _{u \in \Lambda _B} \Vert h_u \Vert \big )^2 \, \int _0^t ds \langle \xi _s, \tilde{\mathcal {N}} \xi _s \rangle \\  &+ C \int _0^t ds \langle \xi _s, \mathcal {N}_g \xi _s \rangle + C N^{-5\beta /8+\epsilon } \big (\sum _{u \in \Lambda _B} \Vert h_u \Vert _2 \big )^2. \end{aligned} \end{aligned}$$

Consider first the choice $h = \check{\sigma }$. By Lemma 6, we have $\sum _u \Vert \check{\sigma }_u \Vert _2 \le C N^{3\kappa /4+\epsilon }$, for any ϵ>0. Since $\tilde{\mathcal {N}} = \mathcal {N}_{\check{\sigma } * \check{\sigma }}$, (131) implies that

$$\begin{aligned} \begin{aligned} \langle \xi _t, \tilde{\mathcal {N}} \xi _t \rangle&\le \text {Re } \langle \Omega , [ A, [ \tilde{\mathcal {N}}, A^* ]] \Omega \rangle \\&\quad + C (1 + N^{-\beta /8 + \epsilon }) \, \int _0^t ds \langle \xi _s, \tilde{\mathcal {N}} \xi _s \rangle + C N^{3\kappa -1-\beta /8+\epsilon } \end{aligned} \end{aligned}$$

Here, we have

$$\begin{aligned}  &   \langle \Omega , [ A, [ \tilde{\mathcal {N}}, A^* ]] \Omega \rangle \\  &   \quad = \int dz dz' dx_1 dx_2 \, (\check{\sigma } * \check{\sigma } ) (z-z') A^{(3)} (x_1 - x_2, x_1 - z') A^{(6)} (z-x_1, z-x_2) \end{aligned}$$

and thus, passing to momentum space and using Lemma 9,

$$\begin{aligned} \big | \langle \Omega , [ A, [ \tilde{\mathcal {N}}, A^* ]] \Omega \rangle \big | = \Big | \sum _{p,q \in \Lambda ^*} \sigma _q^2 A^{(3)}_{p,q} A^{(6)}_{p,q} \Big | \le \sum _{p,q \in \Lambda ^*} \sigma _q^2 |A^{(3)}_{p,q}| | A^{(6)}_{p,q}| \le C N^{3\kappa -1}.\end{aligned}$$

Hence, choosing ϵ>0 small enough, we find

$$\begin{aligned} \begin{aligned} \langle \xi _t, \tilde{\mathcal {N}} \xi _t \rangle \le \;&C N^{3\kappa -1}+ C \int _0^t ds \langle \xi _s, \tilde{\mathcal {N}} \xi _s \rangle .\end{aligned} \end{aligned}$$

By Gronwall, we conclude that $\langle \xi _t, \tilde{\mathcal {N}} \xi _t \rangle \le C N^{3\kappa -1}$ for all t∈[0;1].

Inserting this bound back on the r.h.s. of (131) we find, now for a general $h \in L^2 (\Lambda )$,

$$\begin{aligned} \langle \xi _t, \mathcal {N}_{h \star h} \xi _t \rangle\le &   \text {Re } \langle \Omega , [ A, [ \mathcal {N}_{h\star h}, A^* ]] \Omega \rangle + C \int _0^t ds \langle \xi _s, \mathcal {N}_g \xi _s \rangle \\  &   + C N^{-5\beta /8+\epsilon } \big (\sum _{u \in \Lambda _B} \Vert h_u \Vert _2 \big )^2. \end{aligned}$$

Thus, again by Gronwall’s lemma, we conclude that

$$\begin{aligned} \langle \xi _t, \mathcal {N}_{h*h} \xi _t \rangle \le C | \langle \Omega , [ A, [ \mathcal {N}_{h*h}, A^* ]] \Omega \rangle | + C N^{-5\beta /8+\epsilon } \big (\sum _{u \in \Lambda _B} \Vert h_u \Vert _2 \big )^2 \end{aligned}$$

for all t∈[0;1].

The remark after the lemma follows by the remark that the operator $\ddot{\mathcal {N}}_{h \star h}$ coincides exactly with the term $T_{4,2}$, introduced in (129). □

Finally, we estimate the expectation $\langle \xi , \tilde{\mathcal {V}}_N \xi \rangle$, with $\tilde{\mathcal {V}}_N$ as defined in (108).

#### Lemma 20

We have

$$\begin{aligned} \langle e^{A^* \Theta - \text {h.c.} } \Omega , \tilde{\mathcal {V}}_N e^{A^* \Theta - \text {h.c.} } \Omega \rangle \le C N^{4\kappa -1} \end{aligned}$$

if the parameters α and then *n* in the definition (90) of the cutoff Θ are large enough.

#### Proof

We are going to bound the expectation of

$$\begin{aligned} \tilde{\mathcal {V}}_N^{(0)} = \frac{N^\kappa }{2N} \sum _{p,q,r\in \Lambda ^*} \hat{V}(r/N^{1-\kappa }) (\gamma _{p+r} \gamma _{q-r}\gamma _{q}\gamma _{p}-1) a_{p+r}^* a_{q-r}^* a_q a_p. \end{aligned}$$

All other contributions to $\tilde{\mathcal {V}}_N$ can be controlled analogously, replacing factors of γ-1 by factors of σ. To estimate $\langle \xi , \tilde{\mathcal {V}}_N^{(0)} \xi \rangle$, we observe that, setting $\theta _p = \gamma _p -1$, we have

$$\begin{aligned} \begin{aligned} \gamma _{p+r} \gamma _{q-r} \gamma _q \gamma _p - 1&= \theta _{p+r} \theta _{q-r} \theta _q \theta _p \!+\! \theta _{p+r} \theta _{q-r} \theta _q \!+ \!\theta _{p+r} \theta _{q-r} \theta _p \!+\! \theta _{p+r} \theta _q \theta _p + \theta _{q-r} \theta _q \theta _p \\  &\quad + \theta _{p+r} \theta _{q-r}+ \theta _{p+r} \theta _q + \theta _{p+r} \theta _p + \theta _{q-r} \theta _q+ \theta _{q-r} \theta _p + \theta _{q} \theta _p \\  &\quad + \theta _{p+r} + \theta _{q-r} + \theta _{q} + \theta _{p}. \end{aligned} \end{aligned}$$

Next, we switch to position space. Setting $V_N (x) = N^{2-2\kappa } V (N^{1-\kappa }x)$, we find

132 $$\begin{aligned} \begin{aligned} \tilde{\mathcal {V}}^{(0)}_N= \;&\frac{1}{2}\int dxdy \, V_N(x-y) \, a^* (\check{\theta }_x) a^* (\check{\theta }_y) a (\check{\theta }_x) a (\check{\theta }_y) \\  &+ \int dxdy \, V_N(x-y) \, a^*_{x} a^* (\check{\theta }_y) a (\check{\theta }_x) a (\check{\theta }_y) + \text {h.c.}\\  &+ \int dxdy \, V_N(x-y) \, a^*_{x}a^* (\check{\theta }_y) a_{x} a (\check{\theta }_y) \\  &+ \int dxdy \, V_N(x-y) \, a^*_{x}a^* (\check{\theta }_y) a (\check{\theta }_x) a_{y} \\  &+ \frac{1}{2}\int dxdy \, V_N(x-y) a^*_{x}a^*_{y}a (\check{\theta }_x) a (\check{\theta }_y) + \text {h.c.}\\  &+\int dxdy \, V_N(x-y) \, a^*_{x}a^*_{y}a_{x}a (\check{\theta }_y) + \text {h.c.} \end{aligned} \end{aligned}$$

where we used the notation $\check{\theta }_x (s) = \check{\theta } (x-s)$, for $x \in \Lambda$. When estimating the expectation of all these terms, we can insert, on both sides of the inner products, the cutoff Θ. For the first four terms (the ones without $V_N(x-y)a_x a_y$ factor), this is done as before. As an example, we now show how to insert Θ for the last term in (132) which has to be handled differently (even though we need to use Duhamel for showing that it is negligible).

133 $$\begin{aligned} \begin{aligned} \int&dx dy \, V_N (x-y) \langle \xi , a_x^* a_y^* a_x a (\check{\theta }_y) \xi \rangle \\&\quad = \int dx dy \, V_N (x-y) \langle \Theta \xi , a_x^* a_y^* a_x a (\check{\theta }_y) \Theta \xi \rangle \\&\qquad + \int dx dy \, V_N (x-y) \langle \xi , a_x^* a_y^* a_x a (\check{\theta }_y) (1-\Theta ) \xi \rangle \\&\qquad + \int dx dy \, V_N (x-y) \langle (1-\Theta ) \xi , a_x^* a_y^* a_x a (\check{\theta }_y) \Theta \xi \rangle . \end{aligned} \end{aligned}$$

The second (and analogously the third) term on the r.h.s. can be bounded by

$$\begin{aligned} \begin{aligned} \Big | \int&dx dy \, V_N (x-y) \langle \xi , a_x^* a_y^* a_x a (\check{\theta }_y) (1-\Theta ) \xi \rangle \Big | \\&\quad \le \Big ( \int dx dy \Vert a_x a_y (\mathcal {N}+1) \xi \Vert ^2 \Big )^{1/2} \Big ( \int dx dy \, V_N^2 (x-y) \Vert a_x a (\check{\theta }_y) (\mathcal {N}+1)^{-1} (1-\Theta ) \xi \Vert ^2 \Big )^{1/2} \\&\quad \le \sup _y \Vert \check{\theta }_y \Vert _2 \Vert V_N \Vert _2 \Vert (\mathcal {N}+1)^2 \xi \Vert \Vert (1-\Theta ) \xi \Vert . \end{aligned} \end{aligned}$$

With Lemma 11, we conclude that, for any ν>0,

$$\begin{aligned} \Big | \int dx dy \, V_N (x-y) \langle \xi , a_x^* a_y^* a_x a (\check{\theta }_y) (1-\Theta ) \xi \rangle \Big | \le C N^{-\nu } \end{aligned}$$

if α>0 and then $n \in \mathbb {N}$ are chosen large enough in (92). Hence, we are left only with the first term on the r.h.s. of (133), where ξ has been replaced by $\Theta \xi$.

In Lemma 21 below, we are going to show that

134 $$\begin{aligned} \begin{aligned} \int dx dy \, |V_N (x-y)| \Vert a (\check{\theta }_x) a (\check{\theta }_y) \Theta \xi \Vert ^2 \le C N^{4\kappa -1} N^{-\beta /4 + \epsilon } \\ \int dx dy\, | V_N (x-y)| \Vert a_x a (\check{\theta }_y) \Theta \xi \Vert ^2 \le C N^{4\kappa -1} N^{-\beta /2 + \epsilon }. \end{aligned} \end{aligned}$$

With these bounds and with Cauchy–Schwarz, we can estimate the expectation of the first 4 terms on the r.h.s. of (132).

Let us consider the fifth term on the r.h.s. of (132). Recalling the definition (76) of the kernels $\check{A}^{(3)}, \check{A}^{(6)}$, we find

$$\begin{aligned} [a^*_xa^*_y,-A]=\mathcal {T}_1(x,y)+\mathcal {T}_3(x,y) \end{aligned}$$

with

135 $$\begin{aligned} \mathcal {T}_3(x,y)= &   \int dx_1dx_2 \, \Big ( \check{A}^{(3)} (x_1-x_2,x_1-x)a^*_ya_{x_1}a_{x_2}+\check{A}^{(3)} (x_1-x_2,x_1-y)a^*_xa_{x_1}a_{x_2} \Big ),\nonumber \\ \mathcal {T}_1(x,y)= &   \int dz \check{A}^{(6)} (z-y,z-x)a_z . \end{aligned}$$

Expanding $e^{-A^* \Theta + \text {h.c.} } a_x^* a_y^* e^{A^* \Theta - \text {h.c.} }$ we find

$$\begin{aligned} \begin{aligned} \int dx dy \,&V_N(x-y)\;\langle \xi ,a^*_xa^*_ya(\check{\theta }_x) a(\check{\theta }_y) \xi \rangle \\ = \;&\int _0^1 ds \int dxdy \, V_N(x-y) \;\langle \Theta \xi _s, \mathcal {T}_3 (x,y)e^{-(1-s) A^*\Theta -\text {h.c.} } a(\check{\theta }_x) a(\check{\theta }_y) \Theta \xi \rangle \\  &+\int _0^1 ds \int dxdy \, V_N(x-y) \; \langle \xi _s, \mathcal {T}_1 (x,y) e^{-(1-s)A^*\Theta -\text {h.c.} } a(\check{\theta }_x) a(\check{\theta }_y) \xi \rangle + \mathcal {E}_\Theta \end{aligned} \end{aligned}$$

with an arbitrarily small error $\mathcal {E}_\Theta$. In the second term, we expand the action of $e^{(1-s)A^* \Theta - \text {h.c.} }$ on $\mathcal {T}_1 (x,y)$. We find

$$\begin{aligned} \begin{aligned} \int dx&dy \, V_N(x-y)\;\langle \xi ,a^*_xa^*_ya(\check{\theta }_x) a(\check{\theta }_y) \xi \rangle \\ = \;&\int _0^1 ds \int dxdy \, V_N(x-y) \;\langle \Theta \xi _s, \mathcal {T}_3 (x,y)e^{-(1-s) A^*\Theta -\text {h.c.} } a(\check{\theta }_x) a(\check{\theta }_y) \Theta \xi \rangle \\  &+ \int dxdy \, V_N(x-y) \;\langle \xi , \mathcal {T}_1 (x,y) a(\check{\theta }_x) a(\check{\theta }_y) \xi \rangle \\  &+ \int _0^1 ds \int _0^{1-s} dt \int dx dy \, V_N (x-y) \langle \Theta \xi _{1-t}, [ \mathcal {T}_1 (x,y), A^* ] e^{-t A^* \Theta - \text {h.c.} } a (\check{\theta }_x) a (\check{\theta }_y) \Theta \xi \rangle \\  &+ \mathcal {E}_\Theta . \end{aligned} \end{aligned}$$

Finally, in the second term we expand the action of $e^{A^* \Theta - \text {h.c.} }$on $\mathcal {T}_1 (x,y) a (\check{\theta }_x) a (\check{\theta }_y)$. We arrive at

136 $$\begin{aligned} \begin{aligned} \int dx&dy \, V_N(x-y)\;\langle \xi ,a^*_xa^*_ya(\check{\theta }_x) a(\check{\theta }_y) \xi \rangle \\ = \;&\int _0^1 ds \int dxdy \, V_N(x-y) \;\langle \Theta \xi _s, \mathcal {T}_3 (x,y)e^{-(1-s) A^*\Theta -\text {h.c.} } a(\check{\theta }_x) a(\check{\theta }_y) \Theta \xi \rangle \\  &+ \int _0^1 ds \int _0^{1-s} dt \int dx dy \, V_N (x-y) \langle \Theta \xi _{1-t}, [ \mathcal {T}_1 (x,y), A^* ] e^{-t A^* \Theta - \text {h.c.} } a (\check{\theta }_x) a (\check{\theta }_y) \Theta \xi \rangle \\  &+ \int _0^1 ds \int dxdy \, V_N(x-y) \;\langle \Theta \xi _s, [ \mathcal {T}_1 (x,y), A^*] a(\check{\theta }_x) a(\check{\theta }_y) \Theta \xi _s \rangle \\  &+ \int _0^1 ds \int dxdy \, V_N(x-y) \;\langle \Theta \xi _s, [ a(\check{\theta }_x) a(\check{\theta }_y), A^*] \mathcal {T}_1 (x,y) \Theta \xi _s \rangle \\  &+ \int _0^1 ds \int dxdy \, V_N(x-y) \;\langle \Theta \xi _s, \big [ \mathcal {T}_1 (x,y), [ a(\check{\theta }_x) a(\check{\theta }_y), A^*] \big ] \Theta \xi _s \rangle + \mathcal {E}_\Theta . \end{aligned} \end{aligned}$$

In Lemma 22, we will prove that

137 $$\begin{aligned} \begin{aligned} \int dxdy \, | V_N(x-y)| \Vert \mathcal {T}_3^*(x,y) \Theta \xi _s\Vert ^2&\le CN^{\epsilon } N^{4\kappa -1}N^{-\beta } \\ \int dxdy \, |V_N(x-y)| \Vert [a^*(\check{\theta }_x) a^*(\check{\theta }_y),A]\Theta \xi _s\Vert ^2&\le CN^{\epsilon } N^{4\kappa -1}N^{-\beta /2} \\ \int dxdy\, | V_N(x-y)| \Vert [\mathcal {T}_1^*(x,y),A] \Theta \xi _s\Vert ^2&\le CN^{\epsilon } N^{4\kappa -1}\\ \int dxdy \, |V_N(x-y)| \Vert \mathcal {T}_1(x,y)\Theta \xi _s\Vert ^2&\le CN^{\epsilon } N^{4\kappa -1+\beta /4}.\end{aligned} \end{aligned}$$

Together with (134), these bounds can be used to control the first 4 terms on the r.h.s. of (136). In Lemma 23, we will also show that

138 $$\begin{aligned} \Big | \int dx dy \, V_N (x-y) \, \langle \Theta \xi _s, \big [ \mathcal {T}_1 (x,y), [ a (\check{\theta }_x) a (\check{\theta }_y), A^*] \big ] \Theta \xi _s \rangle \Big | \le C N^{4\kappa -1} \,.\end{aligned}$$

By (136), this implies that

139 $$\begin{aligned} \Big | \int dx dy V_N (x-y) \langle \xi _s a_x^* a_y^* a (\check{\theta }_x) a (\check{\theta }_y) \xi _s \rangle \Big | \le C N^{4\kappa -1}.\end{aligned}$$

Proceeding very similarly as in the proof of (139), we can also show

$$\begin{aligned} \Big | \int dx dy V_N (x-y) \langle \xi a_x^* a_y^* a _x a (\check{\theta }_y) \xi \rangle \Big | \le C N^{4\kappa -1}. \end{aligned}$$

The only term for which the details of the analysis needs some additional adjustments is

$$\begin{aligned} \begin{aligned} \int _0^1 ds \int dxdy \, V_N(x-y) \;\langle \Theta \xi _s, [ a_x a(\check{\theta }_y), A^*] \mathcal {T}_1 (x,y) \Theta \xi _s \rangle , \end{aligned} \end{aligned}$$

which is of order $N^{4\kappa -1 +\epsilon -\beta /4}$.

From (132), we obtain $\langle \xi , \tilde{\mathcal {V}}_N^{(0)} \xi \rangle \le C N^{4\kappa -1}$, as claimed. □

In the next lemma, we show the bounds (134).

#### Lemma 21

As in the proof of Lemma 20, we let $\xi _s = e^{A^*\Theta -\text {h.c.} }\Omega$, for all s∈[0;1], $\theta = \gamma -1$, $V_N (x) = N^{2-2\kappa } V (N^{1-\kappa }x)$. For every ϵ>0, there is C>0 such that

140 $$\begin{aligned} \begin{aligned} T_0&:= \int dxdy \, |V_N(x-y)| \Vert a(\check{\theta }_x) a(\check{\theta }_y) \Theta \xi _s \Vert ^2 \le C N^{4\kappa -1} N^{-\beta /4+\epsilon } \\ T_1&:= \int dxdy \, |V_N(x-y)| \Vert a_x a(\check{\theta }_y) \Theta \xi _s \Vert ^2 \le C N^{4\kappa -1} N^{-\beta /2+\epsilon } \end{aligned} \end{aligned}$$

for all s∈[0;1], if α and then *n* are chosen sufficiently large in the definition (90) of the cutoff Θ.

#### Proof

We write

141 $$\begin{aligned} \begin{aligned} T_0&=\int dxdydrdr' dt dt' \, \check{\theta } (r) \check{\theta } (r')\check{\theta } (t) \check{\theta } (t') |V_N (x-y)| \langle \Theta \xi _s, a^*_{y-t}a^*_{x-r}a_{y-t'}a_{x-r'} \Theta \xi _s\rangle . \end{aligned}\nonumber \\ \end{aligned}$$

Next, we localize $\check{\theta } = \sum _{u \in \Lambda _B} \check{\theta }_u$, where we recall that $\check{\theta }_u = \check{\theta } \mathbb {1}_{B_u}$ and $B_u = \{x\in \Lambda , \inf _{v\in \Lambda _B} |x-v | =|x-u | \}$ with $\Lambda _B = \Lambda \cap \ell _B \mathbb {Z}^3$. With Cauchy–Schwarz, we obtain

$$\begin{aligned} \begin{aligned} |T_0|&\le \sum _{u, u',v,v'}\Big (\int dxdydrdr'dtdt' \; |V_N(x-y)| \mathbb {1}_{B_u}(r) \mathbb {1}_{B_v} (t) \\  &\quad \times \check{\theta }_{u'}(r')^2\check{\theta }_{v'}(t')^2\; \Vert a_{y-t}a_{x-r} \Theta \xi _s\Vert ^2\Big )^{1/2}\\  &\quad \times \Big (\int dxdydrdr'dtdt' \; |V_N(x-y)| \mathbb {1}_{B_{u'}}(r')\mathbb {1}_{B_{v'}} (t') \check{\theta }_{u}(r)^2\check{\theta }_{v}(t)^2 \Vert a_{y-{t'}}a_{x-{r'}}\Theta \xi _s\Vert ^2\Big )^{1/2} \\  &\le CN^{\epsilon } \Vert V_N\Vert _1 \ell _{B}^3 \Big (\sum _{u \in \Lambda _B} \Vert \check{\theta }_u \Vert _2 \Big )^4 \Vert \mathcal {N}^{1/2}\xi _s\Vert ^2. \end{aligned} \end{aligned}$$

With Lemma 6, Lemma 15 and since $\Vert V_N \Vert _1 \le C N^{-1+\kappa }$, we conclude that

$$\begin{aligned} |T_0| \le C N^{4\kappa -1} N^{-\beta /4+\epsilon }. \end{aligned}$$

The second bound in (140) can be shown similarly. In this case, we can remove two factors of $$\check{\theta }$$ and the integrals over $r,r^{\prime }$; as a result, we obtain

$$\begin{aligned} |T_1| \le C N^\epsilon \Vert V_N \Vert _1 \Big (\sum _{u \in \Lambda _B} \Vert \check{\theta }_u \Vert _2 \Big )^2 \langle \xi _s, \mathcal {N}\xi _s\rangle \le C N^{4\kappa -1} N^{-\beta /2+\epsilon }. \end{aligned}$$

□

Next, we show the estimates (137).

#### Lemma 22

As in the proof of Lemma 20, we let $\xi _s = e^{A^*\Theta -\text {h.c.} }\Omega$, for all s∈[0;1], $\theta = 1-\gamma$, $V_N (x) = N^{2-2\kappa } V (N^{1-\kappa }x)$. For any ϵ>0, we have

142 $$\begin{aligned} \int dxdy \, |V_N(x-y)| \Vert \mathcal {T}_3^*(x,y) \Theta \xi _s\Vert ^2\le CN^{\epsilon } N^{4\kappa -1}N^{-\beta } \end{aligned}$$

143 $$\begin{aligned} \int dxdy \, |V_N(x-y)| \Vert [a^*(\check{\theta }_x) a^*(\check{\theta }_y),A]\Theta \xi _s\Vert ^2\le CN^{\epsilon } N^{4\kappa -1}N^{-\beta /2} \end{aligned}$$

144 $$\begin{aligned} \int dxdy \, |V_N(x-y)| \Vert [\mathcal {T}_1^*(x,y),A] \Theta \xi _s\Vert ^2\le CN^{\epsilon } N^{4\kappa -1} \end{aligned}$$

145 $$\begin{aligned} \int dxdy \, |V_N(x-y)| \Vert \mathcal {T}_1(x,y)\Theta \xi _s\Vert ^2\le CN^{\epsilon } N^{4\kappa -1+\beta /4} \end{aligned}$$

if α and then *n* are chosen large enough.

#### Proof

From (135), we find

$$\begin{aligned} \mathcal {T}_3 (x,y) \mathcal {T}_3^* (x,y) = \mathcal {T}_{3,2} + \mathcal {T}_{3,4} + \mathcal {T}_{3,6} \end{aligned}$$

with

$$\begin{aligned} \begin{aligned} \mathcal {T}_{3,2} (x,y)&= \sum \int dx_1 dx_2 \, \check{A}^{(3)} (x_1 - x_2, x_1 - z_1) \check{A}^{(6)} (x_1 - x_2, x_1 - w_1) a_{z_2}^* a_{w_2} \\ \mathcal {T}_{3,4} (x,y)&= \sum \int dx_1 dx_2 dx_3 \check{A}^{(6)} (x_1 - x_2, x_1 - z_1) \check{A}^{(6)} (x_3 - x_2, x_3 - w_1) a_{x_3}^* a_{z_2}^* a_{w_2} a_{x_1} \\ \mathcal {T}_{3,6} (x,y)&= \sum \int dx_1 dx_2 dx_3 dx_4 \check{A}^{(3)} (x_1 - x_2, x_1 - z_1) \check{A}^{(3)} (x_3 - x_4, x_3 - w_1) \\  &\qquad \times a_{z_2}^* a_{x_3}^* a_{x_4}^* a_{x_1} a_{x_2} a_{w_2} \end{aligned} \end{aligned}$$

where the sums run over the four possible choices of $(z_1, z_2), (w_1, w_2) \in \{ (x,y), (y,x) \}$ (i.e. we either have $z_1 = x, z_2 = y$ or $z_1 = y, z_2 = x$ and similarly for $(w_1, w_2)$). Let us denote by $\mathcal {T}_{3,2}^{(0)} (x,y)$ the contribution to $\mathcal {T}_{3,2} (x,y)$ associated with $z_1= w_1 =x$, $z_2 = w_2 = y$. With Cauchy–Schwarz, we can estimate

$$\begin{aligned} \begin{aligned} \Big | \int dx dy&| V_N (x-y)| \langle \xi , \mathcal {T}_{3,2}^{(0)} (x,y) \xi \rangle \Big | \\ \le \;&\Big ( \int dx dy dx_1 dx_2 \, |V_N (x-y)| \check{A}^{(3)} (x_1 - x_2, x_1 - x)^2 \Vert a_y \xi \Vert ^2 \Big )^{1/2} \\  &\times \Big ( \int dx dy dx_1 dx_2 \, |V_N (x-y)| \check{A}^{(6)} (x_1 - x_2, x_1 - x)^2 \Vert a_y \xi \Vert ^2 \Big )^{1/2} \\ \le \;&C \Vert V_N \Vert _1 \Vert \mathcal {N}^{1/2} \xi \Vert ^2 \Vert A^{(6)} \Vert _2 \Vert A^{(3)} \Vert _2 \le C N^{4\kappa -1 - \beta } \end{aligned} \end{aligned}$$

where we used $\Vert V_N \Vert _1 \le C N^{-1 + \kappa }$, the bounds in Lemma 9 (in particular, $\Vert A^{(3)} \Vert _2, A^{(6)} \Vert _2 \le C N^{(3\kappa -1)/2}$) and the estimate $\Vert \mathcal {N}^{1/2} \xi \Vert ^2 \le C N^{3\kappa -1}$ from Lemma 15. The other contributions arising from $\mathcal {T}_{3,2}$ can be bounded similarly.

Next, let us denote by $\mathcal {T}_{3,4}^{(0)} (x,y)$ the contribution to $\mathcal {T}_{3,4} (x,y)$ associated with $z_1 = w_1 = x$ and $z_2 = w_2 = y$. We can estimate

$$\begin{aligned} \begin{aligned} \Big | \int dx dy&|V_N (x-y)| \langle \Theta \xi , \mathcal {T}_{3,4}^{(0)} (x,y) \Theta \xi \rangle \Big | \\ \le \;&\sum _{u, u',v,v'\in \Lambda _B} \int dx dy dx_1 dx_2 dx_3 |V_N(x-y)| \\&\quad \times |\check{A}^{(6)}_{u,v}(x_1-x_2,x_1-x) | |\check{A}^{(6)}_{u',v'}(x_3-x_2,x_3-x) |\Vert a_{x_1}a_y \Theta \xi \Vert \Vert a_{x_3}a_y \Theta \xi \Vert \\ \le \;&\sum _{u, u',v,v' \in \Lambda _B} \Big ( \int dxdy dx_1dx_2dx_3 \, |V_N(x-y)| \mathbb {1}(|x_3-x_2-u' | \le \ell _{B}) \\  &\quad \times |\check{A}^{(6)}_{u,v} (x_1-x_2,x_1-x)|^2 \Vert a_{x_3}a_{y} \Theta \xi \Vert ^2 \Big )^{1/2} \\  &\quad \times \Big ( \int dxdy dx_1dx_2dx_3 \, |V_N(x-y)| \mathbb {1}(|x_1-x_2-u|\le \ell _{B}) \\  &\quad \times |\check{A}^{(6)}_{u',v'}(x_3-x_2,x_3-x)|^2\Vert a_{x_1}a_{y}\Theta \xi \Vert ^2 \Big )^{1/2} \\ \le \;&CN^{\epsilon } \Vert V_N\Vert _1 \Vert \mathcal {N}^{1/2}\xi \Vert ^2\Big (\sum _{u,v \in \Lambda _B} \Vert \check{A}^{(6)}_{u,v} \Vert _2 \Big )^2\le C N^{4\kappa -1-\beta +\epsilon }, \end{aligned} \end{aligned}$$

where we used (94), $\Vert V_N \Vert _1 \le C N^{-1 + \kappa }$, the estimate $\Vert \mathcal {N}^{1/2} \xi \Vert ^2 \le C N^{3\kappa -1}$ from Lemma 15 and Lemma 9 to bound the sum associated to $\check{A}^{(6)}$. The other contributions arising from $\mathcal {T}_{3,4}$ and those arising from $\mathcal {T}_{3,6}$ can be estimated analogously.

Next, we consider the bound (143). Similarly to (135), we have

146 $$\begin{aligned} [a^* (\check{\theta }_x) a^* (\check{\theta }_y),A]= - \int dr dt \;\check{\theta } (r) \check{\theta } (t) \big (\mathcal {T}_1 (x-r,y-t)+ \mathcal {T}_3 (x-r,y-t)\big ). \nonumber \\\end{aligned}$$

Therefore, we find

147 $$\begin{aligned} \begin{aligned} \int dx&dy \, |V_N (x-y)| \Vert \big [ a^* (\check{\theta }_x) a^* (\check{\theta }_y), A \big ] \Theta \xi _s \Vert ^2 \\  &\le \sum _{i=1,3} \int dx dy \, |V_N (x-y)| \Big \Vert \int dr dt \, \check{\theta } (r) \check{\theta } (t) \mathcal {T}_i (x-r, y-t) \Theta \xi _s \Big \Vert ^2.\end{aligned} \end{aligned}$$

To bound the term with i=3, we observe that

$$\begin{aligned} \begin{aligned} \Big \Vert&\int dr dt \, \check{\theta } (x-r) \check{\theta } (y-t) \mathcal {T}_3 (r,t) \Theta \xi _s \Big \Vert ^2 \\&\quad = \int dr dt dr' dt' \check{\theta } (x-r) \check{\theta } (y-t) \check{\theta } (x-r') \check{\theta } (y-t') \sum _{\begin{array}{c} (z_1,z_2)\in \{(r,t),(t,r)\},\\ (w_1,w_2)\in \{(r',t'),(t',r')\} \end{array}} \int dx_1dx_2dx_3dx_4 \, \\  &\times \check{A}^{(3)} (x_1-x_2,x_1-z_1)\check{A}^{(3)} (x_3-x_4,x_3-w_1) \langle \Theta \xi _s,a^*_{z_2}a^*_{x_3}a^*_{x_4}a_{x_1}a_{x_2}a_{w_2} \Theta \xi _s \rangle \\&\qquad + \int dr dt dr' \check{\theta } (x-r) \check{\theta } (y-t) \sum _{\begin{array}{c} (z_1,z_2)\in \{(r,t),(t,r)\} \end{array}} \big (\check{\theta } (x-r') \check{\theta } (y-z_2) +\check{\theta } (x-z_2) \check{\theta } (y-r') \big ) \\  &\qquad \times \int dx_1 dx_2 dx_3 dx_4 \check{A}^{(3)} (x_1-x_2,x_1-z_1)\check{A}^{(3)} (x_3-x_4,x_3-r' ) \langle \Theta \xi _s,a^*_{x_3} a^*_{x_4}a_{x_1}a_{x_2} \Theta \xi _s \rangle \\&\quad = \langle \Theta \xi _s, \tilde{\mathcal {T}}_{3,6}(x,y) \Theta \xi _s \rangle + \langle \Theta \xi _s, \tilde{\mathcal {T}}_{3,4} (x,y) \Theta \xi _s \rangle . \end{aligned} \end{aligned}$$

Let us denote by $\tilde{\mathcal {T}}_{3,6}^{(0)} (x,y)$ the contribution to $\tilde{\mathcal {T}}_{3,6} (x,y)$ associated with the choice $z_1 = r, w_1 = r', z_2 = t, w_2 = t'$. Localizing the kernels $\check{\theta }$ and $\check{A}^{(3)}$ and applying Cauchy–Schwarz, we can estimate

$$\begin{aligned} \begin{aligned} \Big | \int dx dy \,&|V_N (x-y)| \langle \Theta \xi _s, \tilde{\mathcal {T}}^{(0)}_{3,6} (x,y) \Theta \xi _s \rangle \Big | \\ \le \;&\sum _{u, u',v,v',w,w',m,m' \in \Lambda _B}\Big ( \int dr dt dr' dt' dx_1dx_2dx_3dx_4 dxdy \, \, |V_N(x-y)| \\  &\quad \times \check{\theta }_{u}(x-r)^2 \check{\theta }_{u'} (y-t)^2 \, \check{A}^{(3)}_{m',w'} (x_3-x_4,x_3-r')^2 \\  &\quad \times \mathbb {1}_{B_v} (x-r') \mathbb {1}_{B_{v'}}(y-t') \mathbb {1}_{B_{m}} (x_1 - x_2) \mathbb {1}_{B_{w}}(x_1-r) \, \Vert a_{x_1}a_{x_2}a_{t'} \Theta \xi _s\Vert ^2\Big )^{1/2} \\  &\quad \times \Big ( \int dr dt dr' dt' dx_1dx_2dx_3dx_4 dxdy\; |V_N(x-y)| \\  &\quad \times \check{\theta }_{v} (x-r')^2\check{\theta }_{v'} (y-t')^2 \check{A}^{(3)}_{m,w} (x_1-x_2,x_1-r)^2 \\  &\quad \times \mathbb {1}_{B_u}(x-r)\mathbb {1}_{B_{u'}}(y-t) \mathbb {1}_{B_{m'}}(x_3-x_4)\mathbb {1}_{B_{w'}} (x_3-r') \Vert a_{x_4}a_{x_3}a_{t} \Theta \xi _s \Vert ^2\Big )^{1/2} \\\le \;&CN^{\epsilon } \ell _{B}^6 \Vert V_N\Vert _1 \Vert \mathcal {N}^{1/2}\xi _s\Vert ^2 \Big (\sum _{u,v \in \Lambda _B} \Vert \check{A}^{(3)}_{u,v} \Vert _2 \Big )^2 \Big (\sum _{u \in \Lambda _B} \Vert \check{\theta }_u \Vert _2 \Big )^4 \le C N^{4\kappa - 1 - \beta /2 + \epsilon }. \end{aligned} \end{aligned}$$

where we used $\Vert V_N \Vert _1 \le C N^{-1 + \kappa }$, $\sum _u \Vert \check{\theta }_u \Vert _2 \le N^{3\kappa /4+\epsilon }$ from Lemma 6, $\sum _{u,v} \Vert A^{(3)}_{u,v} \Vert _2 \le C N^{(3\kappa -1)/2}$ from Lemma 9 and the estimate $\Vert \mathcal {N}^{1/2} \xi \Vert ^2 \le C N^{3\kappa -1}$ from Lemma 15. The other contributions arising from $\tilde{\mathcal {T}}_{3,6} (x,y)$ can be controlled analogously.

As for the term $\tilde{\mathcal {T}}_{3,4} (x,y)$, we consider the contribution $\tilde{\mathcal {T}}^{(0)}_{3,4} (x,y)$, associated with $z_1 =r$, $z_2 = t$, proportional to $\check{\theta } (x-r) \check{\theta } (x-r') \check{\theta } (y-t)^2$. Localizing all kernels $\check{\theta }, \check{A}^{(3)}$, we find

$$\begin{aligned} \begin{aligned}\Big | \int&dx dy |V_N (x-y)| \langle \Theta \xi _s, \tilde{\mathcal {T}}^{(0)}_{3,4} (x,y) \Theta \xi _s \rangle \Big | \\  &\le \sum _{u, u',v,v',w,w',m \in \Lambda _B} \Big ( \int dxdy dx_1dx_2dx_3 dr dt dr' \, | V_N(x-y) | \check{\theta }_m (y-t)^2\check{\theta }_w (x-r)^2 \\  &\quad \times \check{A}^{(3)}_{u,v} (x_3-x_4,x_3-r')^2 \mathbb {1}_{B_{w'}}(x-r') \mathbb {1}_{B_{v'}}(x_1-r) \mathbb {1}_{B_{u'}}(x_1-x_2) \Vert a_{x_1} a_{x_2} \Theta \xi \Vert ^2 \Big )^{1/2} \\  &\quad \times \Big ( \int dxdy dx_1dx_2dx_3dr dt dr' \, |V_N (x-y)| \check{\theta }_m (y-t)^2 \check{\theta }_{w'} (x-r')^2\\  &\quad \times \check{A}^{(3)}_{u',v'} (x_1-x_2,x_1-r)^2 \mathbb {1}_{B_{w}}(x-r) \mathbb {1}_{B_{v}}(x_3-r') \mathbb {1}_{B_{u}}(x_3-x_4) \Vert a_{x_3} a_{x_4} \Theta \xi \Vert ^2 \Big )^{1/2} \\  &\le CN^{\epsilon } \ell _{B}^6 \Vert V_N\Vert _1 \Vert \mathcal {N}^{1/2}\xi \Vert ^2\Big (\sum _{u,v \in \Lambda _B} \Vert \check{A}^{(3)}_{u,v} \Vert _2 \Big )^2 \Big (\sum _{u \in \Lambda _B} \Vert \check{\theta }_{u} \Vert _2 \Big )^4\le C N^{4\kappa -1-\beta /2+\epsilon }. \end{aligned} \end{aligned}$$

The other contributions arising from $\tilde{\mathcal {T}}_{3,4} (x,y)$ can be handled similarly. As for the term with i=1 on the r.h.s of (147), we compute

$$\begin{aligned} \begin{aligned}&\Big \Vert \int dr dt \; \check{\theta } (x-r) \check{\theta } (y-t) \mathcal {T}_1 (r,t) \Theta \xi _s \Big \Vert ^2 \\&\quad =\int dr dt dr' dt' dx_1dx_2 \, \check{\theta } (x-r) \check{\theta } (y-t) \check{\theta } (x-r') \check{\theta } (y-t') \\  &\qquad \times \check{A}^{(6)} (x_1-t,x_1-r)\check{A}^{(6)} (x_2-t',x_2-r') \langle \xi ,a^*_{x_1}a_{x_2}\xi \rangle . \end{aligned} \end{aligned}$$

Hence, proceeding as we did above to control the terms $\tilde{\mathcal {T}}_{3,6}^{(0)}, \tilde{\mathcal {T}}_{3,4}^{(0)}$, we find

$$\begin{aligned} \begin{aligned} \int&dx dy |V_N (x-y)| \Big \Vert \int dr dt \; \check{\theta } (x-r) \check{\theta } (y-t) \mathcal {T}_1 (r,t) \Theta \xi _s \Big \Vert ^2 \\&\quad \le C \ell _B^6 \Vert V_N \Vert _1 \Vert \mathcal {N}^{1/2} \xi _s \Vert ^2 \Big ( \sum _{u,v \in \Lambda _B} \Vert A^{(6)}_{u,v} \Vert _2 \Big )^2 \Big ( \sum _{w \in \Lambda _B} \Vert \check{\theta }_w \Vert _2 \Big )^4 \le C N^{4\kappa -1-\beta /2+\epsilon }. \end{aligned} \end{aligned}$$

Next, we show (144). From (135), we find

148 $$\begin{aligned} [\mathcal {T}^*_1(x,y),A] = \int dz dx_1 dx_2 \, \check{A}^{(6)} (z-y,z-x)\check{A}^{(3)} (x_1-x_2,x_2-z) a_{x_1}a_{x_2}.\nonumber \\ \end{aligned}$$

Hence

149 $$\begin{aligned} \begin{aligned} \Vert [\mathcal {T}_1^*&(x,y),A] \Theta \xi _s\Vert ^2\\&\quad = \int dz dz' dx_1 dx_2 dx_3 dx_4 \, \check{A}^{(6)} (z-y,z-x) \check{A}^{(3)} (x_1-x_2,x_2-z) \\&\qquad \times \check{A}^{(6)} (z'-y,z'-x) \check{A}^{(3)} (x_3-x_4,x_4-z') \langle \Theta \xi , a^*_{x_3}a^*_{x_4}a_{x_1}a_{x_2} \Theta \xi \rangle . \end{aligned} \end{aligned}$$

Therefore, setting $D^{(6)} (x) = \sup _s |A^{(6)} (x,s)|$ and $D_u^{(6)} (x) = D^{(6)} (x) \mathbb {1}_{B_u} (x)$ for $u \in \Lambda _B$, we obtain

$$\begin{aligned} \begin{aligned} \int&dx dy \, |V_N (x-y)| \Vert [\mathcal {T}_1^* (x,y),A] \Theta \xi _s\Vert ^2 \\ \le \;&\sum _{w,u,v,w',u',v' \in \Lambda _B} \Big ( \int dx dy dz dz' dx_1 dx_2 dx_3 dx_4 \, |V_N (x-y)| D_w^{(6)} (z-y)^2 \\  &\quad \times \check{A}^{(3)}_{u',v'} (x_3 - x_4, x_4 - z')^2 \mathbb {1}_{B_{w'}} (z'-y) \mathbb {1}_{B_u} (x_1 - x_2) \mathbb {1}_{B_v} (x_2 -z) \Vert a_{x_1} a_{x_2} \Theta \xi _s \Vert ^2 \Big )^{1/2} \\&\quad \times \Big ( \int dx dy dz dz' dx_1 dx_2 dx_3 dx_4 \, |V_N (x-y)| D_{w'}^{(6)} (z'-y)^2 \\  &\quad \times \check{A}^{(3)}_{u,v} (x_1 - x_2, x_2 - z)^2 \mathbb {1}_{B_{w}} (z-y) \mathbb {1}_{B_{u'}} (x_3 - x_4) \mathbb {1}_{B_{v'}} (x_4 -z') \Vert a_{x_3} a_{x_4} \Theta \xi _s \Vert ^2 \Big )^{1/2} \\ \le \;&CN^\epsilon \ell _B^6 \Vert V_N \Vert _1 \Vert \mathcal {N}^{1/2} \xi _s \Vert ^2 \, \Big ( \sum _{u,v} \Vert A_{u,v}^{(3)} \Vert _2 \Big )^2 \Big (\sum _w \Vert D^{(6)}_w \Vert _2 \Big )^2 \le C N^{4\kappa -1 + \epsilon } \end{aligned} \end{aligned}$$

where we used the bounds in Lemma 9 and Lemma 15 to estimate $\Vert \mathcal {N}^{1/2} \xi _s \Vert ^2 \le C N^{3\kappa -1}$.

Finally, we consider (145). From (135), setting again $D^{(6)} (x) = \sup _s |A^{(6)} (s,x)|$, we find

$$\begin{aligned} \begin{aligned}&\int dx dy |V_N (x-y)| \Vert \mathcal {T}_1 (x,y) \Theta \xi _s \Vert ^2 \\&\qquad = \int dx dy dz dz' \, |V_N (x-y)| A^{(6)} (z-y, z-x) A^{(6)} (z'-y, z' -x) \langle a_z \Theta \xi _s, a_{z'} \Theta \xi _s \rangle \\&\qquad \le \int dx dy dz dz' \, |V_N (x-y)| D^{(6)} (z-x) D^{(6)} (z' -x) \Vert a_z \Theta \xi _s \Vert \Vert a_{z'} \Theta \xi _s \Vert . \end{aligned} \end{aligned}$$

Localizing $D^{(6)}$ and applying Cauchy–Schwarz, we arrive at

$$\begin{aligned} \begin{aligned} \int dx&dy \, |V_N (x-y)| \Vert \mathcal {T}_1 (x,y) \Theta \xi _s \Vert ^2 \\ \le \;&\sum _{u,u' \in \Lambda _B} \Big ( \int dx dy dz dz' \, |V_N (x-y)| |D_u^{(6)} (z-x)|^2 \mathbb {1}_{B_{u'}} (z'-x) \Vert a_{z'} \Theta \xi _s \Vert ^2 \Big )^{1/2} \\  &\quad \times \Big ( \int dx dy dz dz' \, |V_N (x-y)| |D_{u'}^{(6)} (z'-x)|^2 \mathbb {1}_{B_{u}} (z-x) \Vert a_{z} \Theta \xi _s \Vert ^2 \Big )^{1/2} \\ \le \;&C \ell _B^3 \Vert V_N \Vert _1 \Big ( \sum _u \Vert D^{(6)}_u \Vert _2 \Big )^2 \Vert \mathcal {N}^{1/2} \xi _s \Vert ^2 \le C N^{4\kappa -1+\beta /4+\epsilon }, \end{aligned} \end{aligned}$$

having used in particular $\Vert V_N\Vert \le CN^{\kappa -1}$, Lemma 9 and Lemma 15. □

Finally, we prove (138).

#### Lemma 23

As in the proof of Lemma 20, we let $\xi _s = e^{A^*\Theta -\text {h.c.} }\Omega$, for all s∈[0;1], $\theta = \gamma -1$, $V_N (x) = N^{2-2\kappa } V (N^{1-\kappa }x)$. Then

$$\begin{aligned} \Big | \int dx dy \, V_N (x-y) \, \langle \Theta \xi _s, \big [ \mathcal {T}_1 (x,y), [ a (\check{\theta }_x) a (\check{\theta }_y), A^*] \big ] \Theta \xi _s \rangle \Big | \le C N^{4\kappa -1} \,.\end{aligned}$$

#### Proof

From (146), we find

150 $$\begin{aligned} \begin{aligned} \int dx&dy \, V_N (x-y) \, \langle \Theta \xi _s, \big [ \mathcal {T}_1 (x,y), [ a (\check{\theta }_x) a (\check{\theta }_y), A^*] \big ] \Theta \xi _s \rangle \\  &= \sum _{j=1,3} \int dx dy dr dt \, \check{\theta } (x-r) \check{\theta } (y-t) \langle \Theta \xi _s, \big [ \mathcal {T}_1 (x,y), \mathcal {T}_j^* (r, t)\big ] \Theta \xi _s \rangle .\end{aligned} \end{aligned}$$

Let us first focus on j=3. With (135), we compute

$$\begin{aligned} \begin{aligned} \big [ \mathcal {T}_1&(x,y), \mathcal {T}_3^* (r, t)\big ] \\  &= \int dx_1 dx_2 \, \sum _{\begin{array}{c} (z_1, w_1) \in \{ (x_1,x_2), (x_2,x_1) \} \\ (z_2,w_2) \in \{ (r,t), (t, r) \} \end{array}} A^{(6)} (z_1 - y, z_1 - x) A^{(3)} (x_1 -x_2, x_1 - z_2) a_{w_1}^* a_{w_2}. \end{aligned} \end{aligned}$$

The contribution of the term $[\mathcal {T}_1 (x,y), \mathcal {T}^*_3 (r,t)]^{(0)}$, associated with $z_1 =x_1, w_1 = x_2, z_2 = r, w_2 = t$, to (150) can be bounded, localizing the kernels $\check{\theta }$ and $A^{(3)}$ and introducing $D^{(6)} (x) = \sup _s A^{(6)} (s, x)$, by

$$\begin{aligned} \begin{aligned} \Big | \int dx&dy \, V_N (x-y) \, \langle \Theta \xi _s, [\mathcal {T}_1 (x,y), \mathcal {T}^*_3 (r,t)]^{(0)} \Theta \xi _s \rangle \Big | \\ = \;&\Big | \int dx dy dr dt dx_1 dx_2 V_N (x-y) \check{\theta } (x-r) \check{\theta } (y-t) \\  &\quad \times A^{(6)} (x_1 - y, x_1 -x) A^{(3)} (x_1 -x_2, x_1- r) \langle \Theta \xi _s, a_{x_2}^* a_t \Theta \xi _s \rangle \Big | \\ \le \;&\sum _{u,v,w,w' \in \Lambda _B} \Big ( \int dx dy dr dt dx_1 dx_2 \, |V_N (x-y)| \check{\theta }_w (x-r)^2 \check{\theta }_{w'} (y-t)^2 \\  &\quad \times A^{(3)}_{u,v} (x_1 - x_2, x_1 - r)^2 \Vert a_t \Theta \xi _s \Vert ^2 \Big )^{1/2} \\  &\quad \times \Big ( \int dx dy dr dt dx_1 dx_2 \, |V_N (x-y)| D^{(6)} (x_1 - x)^2 \\  &\quad \times \mathbb {1}_{B_{w'}} (y-t) \mathbb {1}_{B_u} (x_1 - x_2) \mathbb {1}_{B_v} (x_1 - r) \Vert a_{x_2} \Theta \xi _s \Vert ^2 \Big )^{1/2} \\ \le \;&C \ell _B^{9/2} \Vert V_N \Vert _1 \Vert \mathcal {N}^{1/2} \xi _s \Vert ^2 \Vert D^{(6)} \Vert _2 \Big ( \sum _{u,v} \Vert A^{(3)}_{u,v} \Vert _2 \Big ) \Big ( \sum _w \Vert \check{\theta }_w \Vert _2 \Big )^2 \!\le \! C N^{4\kappa -1-\beta /8+ \epsilon }, \end{aligned} \end{aligned}$$

having used $\Vert V_N\Vert _1\le CN^{\kappa -1}$, Lemma 6, Lemma 9 and the estimate $\Vert \mathcal {N}^{1/2} \xi _\tau \Vert ^2 \le CN^{3\kappa -1}$ from Lemma 15. The other contributions from the commutator $[\mathcal {T}_1 (x,y), \mathcal {T}^*_3 (r,t)]$ can be bounded similarly. As for the term on the r.h.s. of (150) with j=1, we compute

$$\begin{aligned} [\mathcal {T}_1 (x,y), \mathcal {T}^*_1 (r,t) ] = \int dz \, \check{A}^{(6)} (z-y, z-x) \check{A}^{(6)} (z-t, z-r). \end{aligned}$$

The contribution of this term to (150) can be estimated switching to Fourier space. We find

$$\begin{aligned} \begin{aligned}&\Big | \int dx dy dr dt dz V_N (x-y) \check{\theta } (x-r) \check{\theta } (y-t) \check{A}^{(6)} (z-y, z-x) \check{A}^{(6)} (z-t, z-r) \Big | \\&\quad = \Big | \sum _{p,q,k \in \Lambda ^*} \hat{V}_N (k) \theta _q \theta _p A^{(6)}_{q-k, p+k} A^{(6)}_{q,p} \Big |\\&\quad \le CN^{-1+\kappa } \sum _{k,p,q \in \Lambda ^*} |\theta _q | | \theta _p| |A^{(6)}_{q-k,p+k}| |A^{(6)}_{p,q}|. \end{aligned} \end{aligned}$$

With the bounds in Lemma 9, we conclude that

$$\begin{aligned}  &   \Big | \int dx dy dr dt dz V_N (x-y) \check{\theta } (x-r) \check{\theta } (y-t) \check{A}^{(6)} (z-y, z-x) \check{A}^{(6)} (z-t, z-r) \Big |\\  &   \quad \le C N^{4\kappa -1}. \end{aligned}$$

□

### Bounds for relevant cubic expectations

In this subsection, we conclude the estimate for the energy of the trial state $\psi _N = W_{N_0} e^{B^* - B} \xi$, proving bounds for the expectations $\langle \xi , (\mathcal {C}_N^* + \mathcal {C}_N) \xi \rangle$, $\langle \xi , \mathcal {V}_N \xi \rangle$ and $\langle \xi , \mathcal {K}\xi \rangle$, with $\xi = e^{A^* \Theta -\text {h.c.} } \Omega$.

#### Lemma 24

In position space, we write the operator $\mathcal {C}_N$ from (107) as

151 $$\begin{aligned} \mathcal {C}_N= N_0^{1/2}\int dxdydz N^{2-2\kappa }V(N^{1-\kappa }(x-y))\check{\sigma }(x-z)a^*_xa^*_ya^*_z. \end{aligned}$$

Recalling the definition (76) of the kernel $\check{A}^{(6)}$, we find

152 $$\begin{aligned} \begin{aligned}&\langle e^{A^*\Theta -\text {h.c.} }\Omega , (\mathcal {C}_N+\mathcal {C}_N^*) e^{A^*\Theta -\text {h.c.} }\Omega \rangle \\&\qquad = 2N_0^{1/2}\int dxdy N^{2-2\kappa }V(N^{1-\kappa }x)\check{\sigma } (y) \check{A}^{(6)}(x,y) + O (N^{4\kappa -1}). \end{aligned} \end{aligned}$$

#### Proof

Expanding $e^{-A^* \Theta +\text {h.c.} } a_x^* a_y^* e^{A^* \Theta - \text {h.c.} }$, recalling the notation $\xi _s = e^{s A^* \Theta - \text {h.c.} } \Omega$, $V_N (x) = N^{2-2-\kappa } V (N^{1-\kappa } x)$ and the definition (135) of the operators $\mathcal {T}_3 (x,y), \mathcal {T}_1 (x,y)$, we find

153 $$\begin{aligned} \begin{aligned}&\langle \xi , \mathcal {C}_N \xi \rangle \\&\quad = N_0^{1/2} \int _0^1 ds \, \int dx dy dz \, V_N (x-y) \check{\sigma } (x-z) \langle \xi _s, [A, a_x^* a_y^*] e^{-(1-s) A^* \Theta + \text {h.c.} } a_z^* \xi \rangle + \mathcal {E}_\Theta \\&\quad = N_0^{1/2} \int _0^1 ds \, \int dx dy dz \, V_N (x-y) \check{\sigma } (x-z) \langle \xi _s, \mathcal {T}_1 (x,y) e^{-(1-s) A^* \Theta + \text {h.c.} } a_z^* \xi \rangle \\&\qquad + N_0^{1/2} \int _0^1 ds \, \int dx dy dz \, V_N (x-y) \check{\sigma } (x-z) \langle \Theta \xi _s,\mathcal {T}_3 (x,y) e^{-(1-s) A^* \Theta + \text {h.c.} } a_z^* \xi \rangle + \mathcal {E}_\Theta \\  &=: S_1 + S_3 + \mathcal {E}_\Theta \end{aligned} \end{aligned}$$

where the error term $\mathcal {E}_\Theta$ includes the contribution of all terms proportional to 1-Θ (to handle these terms and show that $\pm \mathcal {E}_\Theta \le C N^{-\nu }$ for arbitrary ν>0, we can proceed as in (133), choosing α>0 and $n \in \mathbb {N}$ large enough). We can bound

154 $$\begin{aligned} \begin{aligned} |S_3|&\le C N^{1/2} \int _0^1 ds \Big ( \int dx dy |V_N (x-y)| \Vert \mathcal {T}_3^* (x,y) \Theta \xi _s \Vert ^2 \Big )^{1/2} \\  &\quad \times \Big ( \int dx dy \, |V_N (y)| \Big \Vert \int dz \, \check{\sigma } (x-z) a_z^* \xi \Big \Vert ^2 \Big )^{1/2}.\end{aligned} \end{aligned}$$

From Lemma 22, we find

$$\begin{aligned} \int dx dy \, |V_N (x-y)| \Vert \mathcal {T}_3^* (x,y) \Theta \xi _s \Vert ^2 \le C N^{4\kappa -1-\beta +\epsilon } \end{aligned}$$

for any ϵ>0. On the other hand, we have

155 $$\begin{aligned} \int dx \, \Big \Vert \int dz \, \check{\sigma } (x-z) a_z^* \xi _s \Big \Vert ^2 = \Vert \check{\sigma } \Vert _2^2 + \Vert \tilde{\mathcal {N}}^{1/2} \xi _s\Vert ^2 \le C N^{3\kappa /2} \end{aligned}$$

for all s∈[0;1], by Lemma 5 and Lemma 19. With $\Vert V_N \Vert _1 \le C N^{-1+\kappa }$, we conclude that

$$\begin{aligned} |S_3| \le C N^{4\kappa -1} N^{-\beta /4 + \epsilon } \end{aligned}$$

for any ϵ>0.

As for the term $S_1$, we expand

$$\begin{aligned} \begin{aligned} S_1 = \;&N_0^{1/2} \int _0^1 ds \int dx dy dz dz' \, V_N (x-y) \check{\sigma } (x-z) \check{A}^{(6)} (z' - y, z' - x) \\  &\quad \times \langle \xi , e^{(1-s) A^* \Theta -\text {h.c.} } a_{z'} e^{-(1-s) A^* \Theta + \text {h.c.} } a_z^* \xi \rangle \\ = \;&N_0^{1/2} \int dx dy dz dz' \, V_N (x-y) \check{\sigma } (x-z) \check{A}^{(6)} (z' - y, z' - x) \langle \xi , a_{z'} a_z^* \xi \rangle \\&\quad +N_0^{1/2} \int _0^1 ds \int _0^{1-s} dt \int dx dy dz dz' \, V_N (x-y) \check{\sigma } (x-z) \check{A}^{(6)} (z' - y, z' - x) \\  &\quad \times \langle \xi , e^{t A^* \Theta -\text {h.c.} } [ A^*, a_{z'} ] e^{-t A^* \Theta + \text {h.c.} } a_z^* \xi \rangle \end{aligned} \end{aligned}$$

and thus

$$\begin{aligned} \begin{aligned} S_1 = \;&N_0^{1/2} \int dx dy dz \, V_N (x-y) \check{\sigma } (x-z) \check{A}^{(6)} (z - y, z - x) \\  &\quad + N_0^{1/2} \int dx dy dz dz' \, V_N (x-y) \check{\sigma } (x-z) \check{A}^{(6)} (z' - y, z' - x) \langle \xi ,a_z^* a_{z'} \xi \rangle \\&\quad + N_0^{1/2} \int _0^1 ds \int _0^{1-s} dt \int dx dy dz dz' \, V_N (x-y) \check{\sigma } (x-z) \check{A}^{(6)} (z' - y, z' - x) \\  &\quad \times \langle \xi _{1-t}, [ A^*, a_{z'} ] e^{-t A^* \Theta + \text {h.c.} } a_z^* e^{t A^* \Theta -\text {h.c.} } \xi _{1-t} \rangle . \end{aligned} \end{aligned}$$

Expanding $e^{-t A^* \Theta + \text {h.c.} } a_z^* e^{tA^* \Theta -\text {h.c.} }$ and using the symmetry (77), we conclude that

156 $$\begin{aligned} \begin{aligned} S_1&= \; N_0^{1/2} \int dx dy \, V_N (y) \check{\sigma } (x) \check{A}^{(6)} (y, x) \\  &\quad + N_0^{1/2} \int dx dy dz dz' \, V_N (y) \check{\sigma } (x-z) \check{A}^{(6)} (y, x-z') \langle \xi ,a_z^* a_{z'} \xi \rangle \\&\quad + N_0^{1/2} \int _0^1 ds \int _0^{1-s} dt \int dx dy dz dz' \, V_N (y) \check{\sigma } (x-z) \check{A}^{(6)} (y, x-z') \\&\quad \times \langle \xi _{1-t}, [ A^*, a_{z'} ] a_z^* \xi _{1-t} \rangle \\&\quad + N_0^{1/2} \int _0^1 ds \int _0^{1-s} dt \int _0^{t} d\tau \int dx dy dz dz' \, V_N (y) \check{\sigma } (x-z) \check{A}^{(6)} (y, x-z') \\  &\quad \times \langle \xi _{1-t}, [ A^*, a_{z'} ] e^{-\tau A^* \Theta + \text {h.c.} } [ A, a_z^* ] \xi _{1-t +\tau } \rangle \\  &=: S_{1,1} + S_{1,2} + S_{1,3} + S_{1,4}. \end{aligned} \end{aligned}$$

The term $S_{1,1}$ produces the main term on the r.h.s. of (152). To control $S_{1,2}, S_{1,3}, S_{1,4}$, we need bounds for

$$\begin{aligned} \begin{aligned} \int dx \Big \Vert \int dz' \, \check{A}^{(6)} (y, x-z') a_{z'} \xi _s \Big \Vert ^2&= \Vert \mathcal {N}^{1/2}_{A_y \star A_y} \xi _s \Vert ^2 \\ \int dx \Big \Vert \int dz \, \check{A}^{(6)} (y,x-z) \big [ a_z^*, A \big ] \xi _s \Big \Vert ^2&= \Vert \ddot{\mathcal {N}}^{1/2}_{A_y \star A_y} \xi _s \Vert ^2 \end{aligned} \end{aligned}$$

where we set $A_y (x) = \check{A}^{(6)} (y,x)$. To obtain such bounds, we use Lemma 19 (and the subsequent remark). We have

$$\begin{aligned} \begin{aligned}&\langle \Omega , [ A, [ \mathcal {N}_{A_y \star A_y}, A^* ] ] \Omega \rangle = \int dx_1 dx_2 dx_3 dz dz' \, \check{A}^{(6)} (y, z' - z) \check{A}^{(6)} (y, z' - x_3) \\  &\quad \times \check{A}^{(3)} (x_1 - x_2, x_1 - x_3) \check{A}^{(6)} (z-x_1, z-x_2).\end{aligned} \end{aligned}$$

Switching to Fourier, we find

$$\begin{aligned} \langle \Omega , [ A, [ \mathcal {N}_{A_y \star A_y}, A^* ] ] \Omega \rangle = \sum _{p,q,r,s \in \Lambda ^*} A^{(6)}_{r,q} A^{(6)}_{s,q} A^{(3)}_{p,q} A^{(6)}_{p,q} e^{i (r+s) \cdot y}.\end{aligned}$$

With Lemma 5 and Lemma 9, we conclude that

$$\begin{aligned} \begin{aligned} | \langle \Omega , [ A, [ \mathcal {N}_{A_y \star A_y}, A^* ] ] \Omega \rangle |&\le \sum _{p,q,r,s} |A^{(6)}_{r,q}| |A^{(6)}_{s,q}| | A^{(3)}_{p,q}| | A^{(6)}_{p,q} | \\  &\le N \sum _{j=3,6} \sum _{p,q \in \Lambda ^*} (1+ \sigma ^2_q) |A_{p,q}^{(j)}|^2 \le C N^{3\kappa } \end{aligned} \end{aligned}$$

uniformly in *y*.

On the other hand, setting $D^{(6)} (x) = \sup _y |\check{A}^{(6)} (y,x) |$ and $D_u^{(6)} (x)= D^{(6)} (x) \mathbb {1}_{B_u} (x)$ for every $u \in \Lambda _B$, we observe that, with Lemma 9,

$$\begin{aligned} \sup _y \sum _{u \in \Lambda _B} \Vert (A_y)_u \Vert _2 \le \sum _{u \in \Lambda _B} \Vert D^{(6)}_u \Vert _2 \le C N^{3\kappa /4 + 1/2 + \epsilon } \end{aligned}$$

for any ϵ>0. Hence, Lemma 19 (and the remark after it) implies that

$$\begin{aligned} \begin{aligned} \sup _y \Vert \mathcal {N}_{A_y \star A_y}^{1/2} \xi _s \Vert ^2&\le C N^{3\kappa } (1 + N^{-\beta /8+\epsilon }) \\ \sup _y \Vert \ddot{\mathcal {N}}_{A_y \star A_y}^{1/2} \xi _s \Vert ^2&\le C N^{3\kappa - \beta /8 + \epsilon } \end{aligned} \end{aligned}$$

for every ϵ>0. Choosing ϵ>0 small enough, we conclude that

157 $$\begin{aligned} \sup _y \Vert \mathcal {N}_{A_y \star A_y}^{1/2} \xi _s \Vert , \; \sup _y \Vert \ddot{\mathcal {N}}_{A_y \star A_y}^{1/2} \xi _s \Vert \le C N^{3\kappa /2} \end{aligned}$$

for all s∈[0;1]. With (157) and with the bounds

$$\begin{aligned} \begin{aligned} \int dx \Big \Vert \int dz \, \check{\sigma } (x-z) a_z \xi _s \Vert ^2 = \Vert \mathcal {N}_{\check{\sigma } \star \check{\sigma }}^{1/2} \xi _s \Vert = \Vert \tilde{\mathcal {N}}^{1/2} \xi _s \Vert \le C N^{(3\kappa -1)/2} \\ \int dx \Big \Vert \int dz \, \check{\sigma } (x-z) [ a^*_z, A] \xi _s \Vert ^2 = \Vert \ddot{\mathcal {N}}_{\check{\sigma } \star \check{\sigma }}^{1/2} \xi _s \Vert \le C N^{(3\kappa -1)/2} \end{aligned} \end{aligned}$$

which also follow from Lemma 19 and the subsequent remark, we can now estimate the r.h.s. of (156). We have

158 $$\begin{aligned} \begin{aligned} |S_{1,2}| \le \;&C N^{1/2} \int dx dy |V_N (y)| \,\Big \Vert \int dz \, \check{\sigma } (x-z) a_z \xi \Big \Vert \Big \Vert \int dz' \, \check{A}^{(6)}_y (x-z') a_{z'} \xi \Big \Vert \\ \le \;&C N^{1/2} \Vert V_N \Vert _1 \, \Vert \tilde{\mathcal {N}}^{1/2} \xi \Vert \sup _y \Vert \mathcal {N}^{1/2}_{A_y \star A_y} \xi \Vert \le C N^{4\kappa -1} \end{aligned}\qquad \end{aligned}$$

and, similarly,

159 $$\begin{aligned} \begin{aligned} |S_{1,4}| \le \;&C N^{1/2} \Vert V_N \Vert _1 \int _0^1 ds \int _0^{1-s} dt \int _0^{t} d\tau \, \Vert \ddot{\mathcal {N}}^{1/2}_{\check{\sigma } \star \check{\sigma }} \xi _{1-t+\tau } \Vert \, \sup _y \Vert \ddot{\mathcal {N}}^{1/2}_{A_y \star A_y} \xi _{1-t} \Vert \\ \le \;&C N^{4\kappa -1}. \end{aligned}\qquad \end{aligned}$$

To estimate $S_{1,3}$, we expand it once again, writing

160 $$\begin{aligned} \begin{aligned} S_{1,3}&= N_0^{1/2} \int _0^1 ds \int _0^{1-s} dt \int _0^{1-t} d\tau \int dx dy dz dz' \, V_N (y) \check{\sigma } (x-z) \check{A}^{(6)} (y, x-z') \\  &\quad \times \big ( \langle \xi _{\tau }, [A^*, a_{z'} ] [ A, a_z^* ] \xi _\tau \rangle + \langle \xi _{\tau }, [A, [A^*, a_{z'} ]] a_z^* \xi _\tau \rangle \big ) \\  &=: S_{1,3,1} + S_{1.3.2} \end{aligned} \end{aligned}$$

We find

$$\begin{aligned} |S_{1,3,1}| \le C N^{1/2} \Vert V_N \Vert _1 \int _0^1 d\tau \, \Vert \ddot{\mathcal {N}}^{1/2}_{\check{\sigma } \star \check{\sigma }} \xi _\tau \Vert \, \sup _y \Vert \ddot{\mathcal {N}}^{1/2}_{A_y \star A_y} \xi _\tau \Vert \le C N^{4\kappa -1}. \end{aligned}$$

As for $S_{1,3,2}$, we write

161 $$\begin{aligned} S_{1,3,2} = T_0 + T_2 + T_4 + \mathcal {E}_\Theta \end{aligned}$$

with

$$\begin{aligned} \begin{aligned} T_0&= N_0^{1/2} \int _0^1 ds \int _0^{1-s} dt \int _0^{1-t} d\tau \, \int dx dy dz dz' dx_1 dx_2 \, V_N (y) \check{\sigma } (x-z) \check{A}^{(6)} (y, x-z') \\  &\quad \times \check{A}^{(3)} (x_1 - x_2, x_1 -z') \check{A}^{(6)} (x_1 - x_2, x_1 - z) \\ T_2&= N_0^{1/2} \int _0^1 ds \int _0^{1-s} dt \int _0^{1-t} d\tau \int dx dy dz dz' dx_1 dx_2 dx_3 \, V_N (y) \check{\sigma } (x-z) \\  &\quad \times \check{A}^{(6)} (y, x-z') \check{A}^{(6)} (x_1 - x_2, x_1 - z') \check{A}^{(6)} (x_3 - x_2, x_3 - z) \langle \xi _\tau , a_{x_1}^* a_{x_3} \xi _\tau \rangle \\  &\quad +N_0^{1/2} \int _0^1 ds \int _0^{1-s} dt \int _0^{1-t} d\tau \int dx dy dz dz' dx_1 dx_2 dx_3 \, V_N (y) \check{\sigma } (x-z) \\  &\quad \times \check{A}^{(6)} (y, x-z') \check{A}^{(3)} (x_1 - x_2, x_1 - z') \check{A}^{(6)} (x_3 - x_1, x_3 - x_2) \langle \xi _\tau , a_{z}^* a_{x_3} \xi _\tau \rangle \\ T_4&= N_0^{1/2} \int _0^1 ds \int _0^{1-s} dt \int _0^{1-t} d\tau \int dx dy dz dz' dx_1 dx_2 dx_3 dx_4 \, V_N (y) \check{\sigma } (x-z) \\  &\quad \times \check{A}^{(6)} (y, x-z') \check{A}^{(6)} (x_1 -x_2, x_1 - z') \check{A}^{(3)} (x_3 - x_4, x_3 - x_1) \langle \Theta \xi _\tau , a_z^* a_{x_2}^* a_{x_3} a_{x_4} \Theta \xi _\tau \rangle \end{aligned} \end{aligned}$$

and where $\pm \mathcal {E}_\Theta \le C N^{-\nu }$ for arbitrary ν>0, if α>0 and $n \in \mathbb {N}$ are large enough. The identity (161) follows similarly as (129), because the operator

$$\begin{aligned} N_0^{1/2} \int dx dy dz dz' \, V_N (y) \check{\sigma } (x-z), \check{A}^{(6)} (y, x-z') [ A^*, a_{z'} ] a_z^* \end{aligned}$$

has exactly the form of $[\mathcal {N}_g, A^* ]$ in (128), if we choose

$$\begin{aligned} g (z) = N_0^{1/2} \int dx dy \, V_N (y) \check{\sigma } (x) \check{A}^{(6)} (y, x-z). \end{aligned}$$

In contrast to (129), however, the contribution $T_{4,2}$ does not appear in (161) (it corresponds to the term $S_{1,3,1}$). To bound $T_0$, we switch to Fourier space. We find

162 $$\begin{aligned} T_0 = \frac{N_0^{1/2}}{3} \sum _{r,k,p \in \Lambda ^*} \hat{V}_N (r) \sigma _k \, A^{(6)}_{r,k} A^{(3)}_{p,k} A^{(6)}_{p, k}. \end{aligned}$$

With Lemma 9 (and Lemma 5, for the bound $\Vert \sigma \Vert _4^4 \le CN^{3\kappa /2}$), we find

$$\begin{aligned} |T_0| \le C N \Vert \hat{V}_N \Vert _\infty \sum _{j=3,6} \sum _{p,k \in \Lambda ^*} (1 + \sigma _k^2) |A_{p,k}^{(j)}|^2 \le C N^{4\kappa -1}. \end{aligned}$$

Also $T_2$ is conveniently expressed in Fourier space as

163 $$\begin{aligned} \begin{aligned} T_2&= \frac{N_0^{1/2}}{2} \int _0^1 d\tau \, (1- \tau ^2) \\  &\quad \times \sum _{r,k,p \in \Lambda ^*} \hat{V}_N (r) \, \sigma _k \, A_{r,k}^{(6)} \, A_{p,k}^{(3)} A_{p,k}^{(6)} \, \big \langle \xi _\tau , \big ( a_k^* a_k + a_p^* a_p + a_{p+k}^* a_{p+k} \big ) \xi _\tau \big \rangle .\end{aligned} \end{aligned}$$

With $\Vert \hat{V}_N \Vert _\infty \le C N^{-1+\kappa }$, $\Vert \sigma \Vert _\infty \le C N^{\beta /8}$, Lemma 9 (and the identity $A^{(6)}_{p,k} = A^{(6)}_{p+k, -k}$ to handle the contribution proportional to $a_{p+k}^* a_{p+k}$), we obtain

$$\begin{aligned} |T_2| \le C N^{\kappa -\beta /4} \int _0^1 d\tau \, \big \langle \xi _\tau , (\mathcal {N}+ \tilde{\mathcal {N}}) \xi _\tau \rangle . \end{aligned}$$

With Lemmas 15 and 19, we conclude that $|T_2| \le C N^{4\kappa -1-\beta /4}$. Finally, we consider $T_4$. Setting $D^{(6)} (x) = \sup _y |\check{A}^{(6)} (y,x)|$, we can bound

$$\begin{aligned} \begin{aligned} |T_4|&\le C N^{1/2} \int _0^1 d\tau \int dx dy dz' dx_1 dx_2 dx_3 dx_4 \, |V_N (y)| |D^{(6)} (x \!-\! z')| |\check{A}^{(6)} (x_1 \!-\! x_2, x_1 \!-\! z')| \\  &\quad \times |\check{A}^{(3)} (x_3 - x_4, x_3 -x_1)| \Vert a_{x_3} a_{x_4} \Theta \xi _\tau \Vert \Big \Vert \int dz \check{\sigma } (x-z) a_z a_{x_2} \Theta \xi _\tau \Big \Vert .\end{aligned} \end{aligned}$$

Next, we localize the kernels $D^{(6)}, \check{A}^{(6)}$ and we apply Cauchy–Schwarz. We find

$$\begin{aligned} \begin{aligned} |T_4|&\le CN^{1/2} \int _0^1 d\tau \sum _{u_1, u_2,v_1, v_2,w \in \Lambda _B} \\  &\quad \times \Big (\int dx dy dz' dx_1 dx_2 dx_3 dx_4 \, |V_N (y)| |D_w^{(6)} (x-z')|^2 |A_{u_1, v_1}^{(3)} (x_3 - x_4, x_3 -x_1)|^2 \\  &\quad \times \mathbb {1} (|x_1 - x_2 - u_2 |, |x_1 - z' - v_2| \le 10 \ell _B) \Big \Vert \int dz \, \check{\sigma } (x-z) a_{x_2} a_z \Theta \xi _\tau \Big \Vert ^2 \Big )^{1/2} \\&\quad \times \Big (\int dx dy dz' dx_1 dx_2 dx_3 dx_4 \, |V_N (y)| |A_{u_2, v_2}^{(6)} (x_1 - x_2, x_1 -z')|^2 \\  &\quad \times \mathbb {1} (|x-z'-w|, |x_3 - x_4-u_1|, |x_3 - x_1-v_1| \le 10\ell _B) \Vert a_{x_3} a_{x_4}\Theta \xi _\tau \Vert ^2 \Big )^{1/2}. \end{aligned} \end{aligned}$$

Applying Lemma 11 and appropriately shifting the integration variables, we obtain

164 $$\begin{aligned} \begin{aligned} |T_4|&\le C N^{1/2+\epsilon } \int _0^1 d\tau \sum _{u_1, u_2,v_1, v_2,w \in \Lambda _B} \\  &\quad \times \Big (\int dx dy dz' dx_1 dx_2 dx_3 dx_4 \, |V_N (y)| |D_w^{(6)} (z')|^2 |A_{u_1, v_1}^{(3)} (x_4, x_3)|^2 \\  &\quad \times \mathbb {1} ( |x_1| \le 10 \ell _B) \Big \Vert \int dz \, \check{\sigma } (x-z) a_z \xi _\tau \Big \Vert ^2 \Big )^{1/2} \\&\quad \times \Big (\int dx dy dz' dx_1 dx_2 dx_3 dx_4 \, |V_N (y)| |A_{u_2, v_2}^{(6)} (x_2, z')|^2 \\  &\quad \times \mathbb {1} (|x|, |x_1| \le 10\ell _B) \Vert a_{x_3} \xi _\tau \Vert ^2 \Big )^{1/2} \\&\le C N^{1/2+\epsilon } \Vert V_N \Vert _1 \ell _B^{9/2} \big (\sum _u \Vert D^{(6)}_u \Vert _2 \big ) \big ( \sum _{u,v} \Vert \check{A}^{(6)}_{u,v} \Vert _2 \big ) \big ( \sum _{u,v} \Vert \check{A}^{(3)}_{u,v} \Vert _2 \big ) \\  &\quad \times \int _0^1 d\tau \, \Vert \mathcal {N}^{1/2} \xi _\tau \Vert \Vert \tilde{\mathcal {N}}^{1/2} \xi _\tau \Vert \\  &\le C N^{4\kappa -1 -\beta /8 + \epsilon } \end{aligned} \end{aligned}$$

for any ϵ>0, having used $\Vert V_N\Vert _1\le CN^{\kappa -1}$, Lemma 9 to estimate the sums associated to $\check{A}^{(3)}$, $\check{A}^{(6)}$ and $\check{D}^{(6)}$, and the estimate $\Vert \mathcal {N}^{1/2} \xi _\tau \Vert ^2+\Vert \tilde{\mathcal {N}}^{1/2} \xi _\tau \Vert ^2 \le CN^{3\kappa -1}$ from Lemma 15 and Lemma 19. Choosing ϵ>0 small enough, we conclude that $|T_4| \le C N^{4\kappa -1}$ and therefore that $|S_{1,3}| \le C N^{4\kappa -1}$. This concludes the proof of (152). □

In the next lemma, we estimate the expectation of the potential energy operator $\mathcal {V}_N$.

#### Lemma 25

From (107), recall the operator

165 $$\begin{aligned} \mathcal {V}_N= \frac{1}{2}\int dxdy N^{2-2\kappa }V(N^{1-\kappa }(x-y))a^*_xa^*_ya_x a_y. \end{aligned}$$

We have

166 $$\begin{aligned} \begin{aligned} \langle e^{A^*\Theta -\text {h.c.} }\Omega ,&\mathcal {V}_N e^{A^*\Theta -\text {h.c.} }\Omega \rangle \\  &= \frac{1}{2}\int dxdy N^{2-2\kappa }V(N^{1-\kappa }x)\check{A}^{(6)}(x,y)^2 + O (N^{4\kappa -1}). \end{aligned} \end{aligned}$$

#### Proof

With $\xi _s = e^{sA^* \Theta -\text {h.c.} } \Omega$, we find, for any t∈[0;1],

167 $$\begin{aligned} \begin{aligned}&\langle e^{t A^*\Theta -\text {h.c.} }\Omega , \mathcal {V}_N e^{t A^*\Theta -\text {h.c.} }\Omega \rangle = \int _0^t ds \langle \xi _s, \big ( [\mathcal {V}_N, A^*] + \text {h.c.} \big ) \xi _s \rangle + \mathcal {E}_\Theta \end{aligned} \end{aligned}$$

where, as usual, $\mathcal {E}_\Theta$ collects all contributions proportional to 1-Θ. Note that we used $[\mathcal {V}_N, \Theta ]=0$ as both are position space multiplication operators for each fixed number of particles. Using Lemma 11 and proceeding similarly as in (133), we can include this term in the error $O (N^{4\kappa -1})$, if we choose α>0 and $n \in \mathbb {N}$ large enough. We write

168 $$\begin{aligned} \begin{aligned} [\mathcal {V}_N,A^*] +\text {h.c.} =&\; \frac{1}{2}\int dxdydz \, V_N (x-y) \check{A}^{(6)}(x-y,x-z) a_x^*a_y^*a_z^* + \text {h.c.} \\  &+\int dx dy dz_1 dz_2 \, V_N (x-y) \check{A}^{(3)}(z_1-z_2,z_1-x)a_x^*a_y^* a_{z_1}^* a_{z_2}^* a_y + \text {h.c.} \\=&\; T_3+T_5, \end{aligned} \end{aligned}$$

where we used again the notation $V_N (x) = N^{2-2\kappa } V (N^{1-\kappa } x)$. To bound the expectation $\langle \xi _s, T_5 \xi _s \rangle$, we introduce the operator

$$\begin{aligned} \mathcal {W}_N =\frac{1}{2}\int dxdy \, |V_N(x-y)|\;a^*_{x}a^*_{y}a_{x}a_{y}. \end{aligned}$$

and we localize the kernel $\check{A}^{(3)}$. Applying Cauchy–Schwarz and Lemma 11, we find

$$\begin{aligned} \begin{aligned}&|\langle \xi _s,T_{5} \xi _s \rangle | \\&\quad \le \; C\sum _{u,v\in \Lambda _{\sigma }} \Big ( \int dxdydz_1dz_2 |V_N(x-y)| |\check{A}^{(3)}_{u,v}(z_1-z_2,z_1-x)|^2\Vert a_y \Theta \xi _s\Vert ^2 \Big )^{\frac{1}{2}} \\&\qquad \times \Big ( \int dxdydz_1dz_2 |V_N(x-y)| \mathbb {1}( |z_1-z_2-u|,|z_1-x+v|\le \ell _B) \Vert a_{z_1}a_{z_2} a_xa_y\Theta \xi _s\Vert ^2 \Big )^{\frac{1}{2}}\\&\qquad +\mathcal {E}_\Theta \\&\quad \le CN^{\epsilon } \Vert V_N\Vert _1^{1/2} (\sum _{u,v\in \Lambda _{\sigma }}\Vert \check{A}^{(3)}_{u,v}\Vert ) \Vert \mathcal {N}^{1/2} \xi _s \Vert \Vert \mathcal {W}_N^{1/2} \xi _s \Vert + \mathcal {E}_\Theta \end{aligned} \end{aligned}$$

and thus, using $\Vert V_N \Vert _1 \le C N^{-1 + \kappa }$, the estimate $\Vert \mathcal {N}^{1/2} \xi \Vert ^2 \le C N^{3\kappa -1}$ from Lemma 15 and Lemma 9 to bound the sum associated to $\check{A}^{(3)}_{u,v}$, we find

169 $$\begin{aligned} |\langle \xi _s, T_5 \xi _s \rangle | \le C N^{4\kappa -1} + C N^{-\beta + \epsilon } \langle \xi _s, \mathcal {W}_N \xi _s \rangle \end{aligned}$$

for all s∈[0;t]. We consider now the expectation of the cubic term $T_3$ in (168). We observe that this term has exactly the same form as the cubic operator $\mathcal {C}_N$ considered in Lemma 24, with $N_0^{1/2} \check{\sigma } (x-z)$ replaced by $\check{A}^{(6)} (x-y, x-z)/2$. Thus, we can bound the expectation of $T_3$ exactly as we estimated $\mathcal {C}_N$. Similarly as in (153), we write

$$\begin{aligned} \langle \xi _s, T_3 \xi _s \rangle =\text {Re } \big ( \widetilde{S}_1 + \widetilde{S}_3 \big ) + \mathcal {E}_\Theta \end{aligned}$$

where

$$\begin{aligned} \begin{aligned} \widetilde{S}_1&= \int _0^s d\tau \int dx dy dz \, V_N (x-y) \check{A}^{(6)} (x-y,x-z) \langle \xi _\tau , \mathcal {T}_1 (x,y) e^{-(1-\tau ) A^* \Theta + \text {h.c.} } a_z^* \xi _s \rangle \\ \widetilde{S}_3&= \int _0^s d\tau \int dx dy dz \, V_N (x-y) \check{A}^{(6)} (x-y,x-z) \langle \xi _\tau , \mathcal {T}_3 (x,y) e^{-(1-\tau ) A^* \Theta + \text {h.c.} } a_z^* \xi _s \rangle . \end{aligned} \end{aligned}$$

Replacing the bound (155) by the estimate $\sup _y \Vert \check{A}^{(6)} (y, \cdot ) \Vert _2 \le C N^{3\kappa /4+1/2}$ and by (157), we obtain $|\widetilde{S}_3| \le C N^{4\kappa -1-\beta /4+\epsilon }$. Proceeding as in the derivation of (156), we write

$$\begin{aligned} \widetilde{S}_1= &   s \int dx dy \, V_N (y) \, |\check{A}^{(6)} (y, x)|^2 \\    &   + s \int dx dy dz dz' \, V_N (y) \check{A}^{(6)} (y, x-z) \check{A}^{(6)} (y, x-z') \langle \xi ,a_z^* a_{z'} \xi \rangle \\  &   + \int _0^s d\tau _1 \int _0^{1-\tau _1} d\tau _2 \int dx dy dz dz' \, V_N (y) \check{A}^{(6)} (y,x-z) \check{A}^{(6)} (y, x-z') \\  &   \times \langle \xi _{1-\tau _2}, [ A^*, a_{z'} ] a_z^* \xi _{1-\tau _2} \rangle \\  &   + \int _0^s d\tau _1 \int _0^{1-\tau _1} d\tau _2 \int _0^{\tau _2} d\tau _3 \int dx dy dz dz' \, V_N (y) \check{A}^{(6)} (y,x-z) \check{A}^{(6)} (y, x-z') \\  &   \times \langle \xi _{1-\tau _2}, [ A^*, a_{z'} ] e^{-\tau A^* \Theta + \text {h.c.} } [ A, a_z^* ] \xi _{1-\tau _2+\tau _3} \rangle \\=: &   \widetilde{S}_{1,1} + \widetilde{S}_{1,2} + \widetilde{S}_{1,3} + \widetilde{S}_{1,4}. \end{aligned}$$

Analogously as in (158), (159), replacing the bounds

$$\begin{aligned} N^{1/2} \Vert \tilde{\mathcal {N}}^{1/2} \xi \Vert \le C N^{3\kappa /2}, \quad N^{1/2} \Vert \mathcal {N}^{1/2}_{\check{\sigma } \star \check{\sigma }} \xi _{1-t-\tau } \Vert \le C N^{3\kappa /2} \end{aligned}$$

with the estimates (157), we obtain $|\widetilde{S}_{1,2}|, |\widetilde{S}_{1,4}| \le CN^{4\kappa -1}$. Similarly to (160), we can decompose $\widetilde{S}_{1,3} = \widetilde{S}_{1,3,1} + \widetilde{S}_{1,3,2}$ with

$$\begin{aligned} \begin{aligned} \widetilde{S}_{1,3,1}&= \int _0^s d\tau _1 \int _0^{1-\tau _1} d\tau _2 \int _0^{1-\tau _2} d\tau _3 \\&\hspace{.5cm} \times \int dx dy dz dz' \, V_N (y) \check{A}^{(6)} (y,x-z) \check{A}^{(6)} (y, x-z') \langle \xi _{\tau _3}, [A^*, a_{z'} ] [ A, a_z^* ] \xi _{\tau _3} \rangle \\ \widetilde{S}_{1,3,2}&= \int _0^s d\tau _1 \int _0^{1-\tau _1} d\tau _2 \int _0^{1-\tau _2} d\tau _3 \\&\hspace{.5cm} \times \int dx dy dz dz' \, V_N (y) \check{A}^{(6)} (y,x-z) \check{A}^{(6)} (y, x-z') \langle \xi _{\tau _3}, [A, [A^*, a_{z'} ]] a_z^* \xi _{\tau _3} \rangle . \end{aligned} \end{aligned}$$

Replacing again $N^{1/2} \Vert \ddot{\mathcal {N}}^{1/2}_{\check{\sigma } \star \check{\sigma }} \xi _\tau \Vert \le C N^{3\kappa /2}$ with (157), we can show $|\widetilde{S}_{1,3,1}| \le C N^{4\kappa -1}$. As for the term $\widetilde{S}_{1,3,2}$, we can write it as $\widetilde{S}_{1,3,2} = \widetilde{T}_0 + \widetilde{T}_2 + \widetilde{T}_4$, where $\widetilde{T}_0, \widetilde{T}_2, \widetilde{T}_4$ are defined like the contributions $T_0, T_2, T_4$ in (161), but with $N_0^{1/2} \check{\sigma } (x-z)$ replaced by $\check{A}^{(6)} (y, x-z)$ (and with adjusted time integrals). The bound $|\widetilde{T}_4| \le C N^{4\kappa -1-\beta /8 + \epsilon }$ can be shown exactly as in (164) (replacing, in the last step, the estimate $N^{1/2} \Vert \tilde{\mathcal {N}}^{1/2} \xi _\tau \Vert \le C N^{3\kappa /2}$ by (157)). Similarly to (162), (163), we write

$$\begin{aligned} \begin{aligned} \widetilde{T}_0&= \frac{s}{2} (1-s^2/3) \sum _{r,q,k,p \in \Lambda ^*} \hat{V}_N (r) A_{q,k}^{(6)} A^{(6)}_{q+r, k} A^{(3)}_{p,k} A^{(6)}_{p,k} \\ \widetilde{T}_2&= \int _0^1 d\tau \,\big [s (1-\tau ) - \frac{(s-\tau )^2}{2} \chi (\tau \le s) \big ] \\&\quad \times \sum _{r,q,k,p \in \Lambda ^*} \hat{V}_N (r) A_{q,k}^{(6)} A^{(6)}_{q+r, k} A^{(3)}_{p,k} A^{(6)}_{p,k} \big \langle \xi _\tau , \big (a_p^* a_p + a_k^* a_k + a_{p+k}^* a_{p+k} \big ) \xi _\tau \big \rangle \end{aligned} \end{aligned}$$

in Fourier space. With the bounds in Lemma 9, Lemma 15, Lemma 19, we find that $|\widetilde{T}_0| \le C N^{4\kappa -1}$ and $|\widetilde{T}_2| \le C N^{4\kappa -1 - \beta /4 + \epsilon }$, for any ϵ>0. Choosing ϵ>0 small enough, we conclude that $|\widetilde{S}_{1,3,2}| \le C N^{4\kappa -1}$ and thus that

$$\begin{aligned} \langle \xi _s, T_3 \xi _s \rangle \le s \int dx dy \, V_N (y) |\check{A}^{(6)} (y,x)|^2 + C N^{4\kappa -1} \end{aligned}$$

for all s∈[0;1]. Combining this bound with (167), (168), (169), we conclude that

170 $$\begin{aligned}  &   \langle e^{t A^* \Theta -\text {h.c.} } \Omega , \mathcal {V}_N e^{t A^* \Theta -\text {h.c.} } \Omega \rangle \nonumber \\    &   \quad \le \frac{t^2}{2} \int dx dy \, V_N (y) |\check{A}^{(6)} (y,x)|^2 + C N^{-\beta + \epsilon } \int _0^t ds \, \langle \xi _s, \mathcal {W}_N \xi _s \rangle + C N^{4\kappa -1}. \qquad \qquad \end{aligned}$$

Similarly, replacing $V_N$ with $|V_N|$ (and observing that all estimates only depend on $\Vert V_N \Vert _1$, $\Vert \hat{V}_N \Vert _2$), we obtain171With , we findChoosing ϵ>0 small enough, (171) implies that

$$\begin{aligned} \langle \xi _t, \mathcal {W}_N \xi _t \rangle \le C N^{5\kappa /2} + C \int _0^t ds \, \langle \xi _s, \mathcal {W}_N \xi _s \rangle \end{aligned}$$

for all t∈[0;1]. By Gronwall’s Lemma, we conclude that $\langle \xi _t, \mathcal {W}_N \xi _t \rangle \le C N^{5\kappa /2}$ for all t∈[0;1]. Inserting this bound in (170) and observing that $5\kappa /2 - \beta = 4\kappa -1 - \beta /2$, we obtain (166). □

Finally, we evaluate the expectation of the kinetic energy operator in our trial state $\xi = e^{A^* \Theta - \text {h.c.} } \Omega$.

#### Lemma 26

We have

$$\begin{aligned} \begin{aligned} \langle e^{A^* \Theta - \text {h.c.} }&\Omega , \mathcal {K}e^{A^* \Theta - \text {h.c.} } \Omega \rangle \\  &= - N^{1/2} \int dx dy \, N^{2-2\kappa } (Vf) (N^{1-\kappa } x) \check{\sigma } (y) \check{A}^{(6)} (x,y) + O (N^{4\kappa -1}). \end{aligned} \end{aligned}$$

#### Proof

We use the notation $\xi _t = e^{t A^* \Theta - \text {h.c.} } \Omega$. Since $\langle \Omega , \mathcal {K}\Omega \rangle = 0$, we have

172 $$\begin{aligned} \begin{aligned} \langle \xi _t, \mathcal {K}\xi _t \rangle = \;&2 \text {Re } \int _0^t ds \, \langle \xi _s, [ \mathcal {K}, A^* \Theta ] \xi _t \rangle \\ = \;&2\text {Re } \int _0^t ds \, \langle \xi _s, [\mathcal {K}, A^*] \Theta \xi _s \rangle + 2\text {Re } \int _0^t ds \, \langle \xi _s, A^* [ \mathcal {K}, \Theta ] \xi _s \rangle . \end{aligned} \end{aligned}$$

Expressing the cubic operator $A^*$ in momentum space, as

$$\begin{aligned} A^* = \sum _{p,q \in \Lambda ^*} A_{p,q} a_{p+q}^* a_{-p}^* a_{-q}^* \,, \end{aligned}$$

and recalling the definition (63) of the coefficients $A_{p,q}$, we find173With Lemma 11, it follows that

174 $$\begin{aligned} 2 \text {Re } \int _0^t ds \, \langle \xi _s, [ \mathcal {K}, A^*] \Theta \xi _s \rangle = 2 \text {Re } \int _0^t ds \, \langle \xi _s, [\mathcal {K}, A^* ] \xi _s \rangle + O (N^{4\kappa -1}) \end{aligned}$$

if we choose the parameter α>0 in the definition of the cutoff Θ large enough. Moreover, from (173), we obtain $\Vert (\mathcal {N}+1)^{-3/2} [ \mathcal {K}, A^* ] \Vert \le C N^{1 + \kappa /4}$. From Lemma 11, this implies the rough but useful a-priori bound

175 $$\begin{aligned} \Big | \int _0^t ds \langle \xi _s, [\mathcal {K}, A^* ] \Theta \xi _s \rangle \Big | \le C N^{5\kappa /2 + 1}. \end{aligned}$$

Let us now consider the second term on the r.h.s. of (172). We observe that the cutoff Θ preserves the number of particles and, on the *m*-particle sector of the Fock space $\mathcal {F}= \oplus _{m=0}^\infty L^2_s (\Lambda ^m)$, it acts as multiplication operator with the function

$$\begin{aligned} F_m (x_1, \dots , x_m) = \Theta \Big ( \int _{\Lambda } dw \, \big ( \sum _{j=1}^m \chi _{\ell _B} (x_j - w) + 1 \big )^n / N^\alpha \Big ).\end{aligned}$$

Since also the kinetic energy operator $\mathcal {K}$ leaves the number of particles invariant, on the *m*-particle sector, the action of the commutator $[\mathcal {K}, \Theta ]$ is given by

176 $$\begin{aligned} \begin{aligned} \sum _{j=1}^m \big [&-\Delta _{x_j}, F_m (x_1, \dots , x_m) \big ] \\  &= - 2 \sum _{j=1}^m (\nabla _{x_j} F_m) (x_1, \dots , x_m) \cdot \nabla _{x_j} - \sum _{j=1}^m (\Delta _{x_j} F_m) (x_1, \dots , x_m).\end{aligned} \end{aligned}$$

We compute

$$\begin{aligned} \begin{aligned} \nabla _{x_j} F_m (x_1, \dots , x_m) = \;&n N^{-\alpha } \Theta ' \Big ( \int _{\Lambda } dw \, \big ( \sum _{j=1}^m \chi _{\ell _B} (x_j - w) + 1 \big )^n / N^\alpha \Big ) \\  &\times \int _{\Lambda } dw \, \big ( \sum _{j=1}^m \chi _{\ell _B} (x_j - w) + 1 \big )^{n-1} \nabla \chi _{\ell _B} (x_j - w).\end{aligned} \end{aligned}$$

The fact that $\Theta^{\prime }$ is supported in the interval [1; 2] implies, on the one hand, that

$$\begin{aligned} [\mathcal {K}, \Theta ] = \mathbb {1}_{[1;\infty )} (\mathcal {L}_n / N^\alpha ) [ \mathcal {K}, \Theta ] \end{aligned}$$

and, on the other hand, the bound

$$\begin{aligned} \begin{aligned} |\nabla _{x_j} F_m (x_1, \dots , x_m)|^2 \le \;&n^2 N^{-2\alpha } \Big | \Theta ' \Big ( \int _{\Lambda } dw \, \big ( \sum _{j=1}^m \chi _{\ell _B} (x_j - w) + 1 \big )^n / N^\alpha \Big ) \Big |^2 \\  &\times \Big | \int _{\Lambda } dw \big ( \sum _{j=1}^m \chi _{\ell _B} (x_j - w) + 1 \big )^{n-1} |\nabla \chi _{\ell _B} (x_j - w)| \Big |^2 \\ \le \;&C \Vert \nabla \chi _{\ell _B} \Vert _\infty ^2 \le C \ell _B^{-2} \end{aligned} \end{aligned}$$

for a constant C>0 (depending on the parameter $n \in \mathbb {N}$). Similarly, we find

$$\begin{aligned} |\Delta _{x_j} F_m (x_1, \dots , x_m)| \le C \ell _B^{-2}. \end{aligned}$$

From (176), we conclude that

$$\begin{aligned} \Vert \frac{1}{(\mathcal {N}+1)} [\mathcal {K}, \Theta ] \xi _s \Vert \le C \ell _B^{-2} \Vert (\mathcal {K}+1)^{1/2} \xi _s \Vert \end{aligned}$$

for every s∈[0;1] and therefore that

$$\begin{aligned} \Big | \int _0^t ds \, \langle \xi _s, A^* [ \mathcal {K}, \Theta ] \xi _s \rangle \Big | \!\le \! C \ell _B^{-2} \int _0^t ds \, \Vert (\mathcal {N}+1) \mathbb {1}_{[1;\infty )} (\mathcal {L}_n / N^\alpha ) A \xi _s \Vert \Vert (\mathcal {K}\!+\! 1)^{1/2} \xi _s \Vert . \end{aligned}$$

With Lemma 11 we obtain that, for any ν>0,

177 $$\begin{aligned} \Big | \int _0^t ds \, \langle \xi _s, A^* [ \mathcal {K}, \Theta ] \xi _s \rangle \Big | \le C N^{-\nu } \int _0^t ds \Vert (\mathcal {K}+1)^{1/2} \xi _s \Vert \end{aligned}$$

for every t∈[0;1], if α>0 is large enough. Inserting the last bound together with (175) in (172), we arrive at

$$\begin{aligned} \langle \xi _t, \mathcal {K}\xi _t \rangle \le C N^{5\kappa /2+1} + C N^{-\nu } \int _0^t ds \langle \xi _s, \mathcal {K}\xi _s \rangle \end{aligned}$$

for every t∈[0;1]. By Gronwall’s Lemma, we obtain $\langle \xi _t, \mathcal {K}\xi _t \rangle \le C N^{5\kappa /2+1}$ for all t∈[0;1]. Plugging this back into (177), we conclude from (172) and (174) that

178 $$\begin{aligned} \langle \xi _t, \mathcal {K}\xi _t \rangle = 2 \text {Re } \int _0^t ds \langle \xi _s, [\mathcal {K}, A^* ] \xi _s \rangle + O (N^{4\kappa -1}) \end{aligned}$$

if the parameter α>0 is large enough. Translating (173) to position space, we find

179 $$\begin{aligned} \begin{aligned} [\mathcal {K}, A^* ] = \;&- \sqrt{N} \int dx dy dz \, N^{2-2\kappa } (Vf) (N^{1-\kappa } (x-y)) \check{\sigma } (x-z) a_x^* a_y^* a_z^* \\  &+ \int dx dy dz \, \check{A}_{\mathcal {K}}^\text {low} (x-y,x-z) \, a_x^* a_y^* a_z^* \end{aligned} \end{aligned}$$

with $A_{\mathcal {K}}^\textrm{low}$ as defined in (84). To bound the second term, with the kernel $A_{\mathcal {K}}^\textrm{low}$, we apply Lemma 14; using the bounds $\Vert A_{\mathcal {K}}^\textrm{low} \Vert _2 \le C N^{(5\kappa -1)/2}$ and

$$\begin{aligned} \sum _{u,v \in \Lambda _B} \Vert \check{A}^\text {low}_{\mathcal {K},u,v} \Vert _2 \le C N^{(5\kappa -1)/2 + \epsilon } \end{aligned}$$

from Lemma 10, we conclude that this contribution is negligible, of the order $N^{4\kappa -1}$. As for the first term on the r.h.s. of (179), we compare it with (151) and we observe that it has exactly the same form as the cubic operator $\mathcal {C}_N$, just with the prefactor $N_0^{1/2}$ replacing by $N^{1/2}$ and with the potential *V* replaced by *Vf*. Hence, proceeding exactly as in Lemma 24, we conclude that

$$\begin{aligned} 2 \text {Re } \langle \xi _s, [\mathcal {K}, A^* ] \xi _s \rangle= &   -2s N^{1/2} \int dx dy \, N^{2-2\kappa } (Vf) (N^{1-\kappa } x) \check{\sigma } (y) \check{A}^{(6)} (x,y)\\  &   + O (N^{4\kappa -1}) \end{aligned}$$

and therefore, from (178), that

$$\begin{aligned} \begin{aligned} \langle e^{A^* \Theta -\text {h.c.} } \Omega ,&\mathcal {K}e^{A^* \Theta -\text {h.c.} } \Omega \rangle \\  &= -N^{1/2} \int dx dy \, N^{2-2\kappa } (Vf) (N^{1-\kappa } x) \check{\sigma } (y) \check{A}^{(6)} (x,y) + O (N^{4\kappa -1}) \,. \end{aligned} \end{aligned}$$

□

### Proof of Theorem 2

We combine the results of Lemma 24, Lemma 25 and Lemma 26.

#### Lemma 27

Let $\mathcal {C}_N$ be the cubic operator defined in (108), $\mathcal {V}_N$ the quartic potential energy operator and $\mathcal {K}$ the quadratic kinetic energy operator. Then, we have

#### Proof

From Lemmas 24 and 26, we find, writing all contributions in momentum space180Note that we have replaced $N_0$ by *N* in the contribution from $\mathcal {C}_N$ using that $|N_0^{1/2}-N^{1/2} |\le N^{3\kappa /2-1}$ and that the whole term is of order $N^{5\kappa /2}$, see Lemma 5 and 7. As for the expectation of $\mathcal {V}_N$, we find, from Lemma 25,

$$\begin{aligned} \langle \xi , \mathcal {V}_N \xi \rangle = \frac{N^{\kappa -1}}{2} \sum _{p,q,r \in \Lambda ^*} \hat{V} (r/N^{1-\kappa }) A^{(6)}_{p,q} A^{(6)}_{p-r,q} + O(N^{4\kappa -1}).\end{aligned}$$

Since $A^{(6)}_{p,q} = A^{(6)}_{-p-q,q}$, we have

$$\begin{aligned}  &   \frac{N^{\kappa -1}}{2} \sum _{p,q,r \in \Lambda ^*} \hat{V} (r/N^{1-\kappa }) A^{(6)}_{p,q} A^{(6)}_{p-r,q} \\  &   \quad = N^{\kappa -1} \sum _{p,q,r \in \Lambda ^*} \hat{V} (r/N^{1-\kappa })(A_{p-r,q} + A_{q,p-r} + A_{-p-q+r, p-r}) A^{(6)}_{p,q} \\  &   \quad = N^{\kappa -1} \sum _{p,q,r \in \Lambda ^*} \hat{V} (r/N^{1-\kappa })(A_{p-r,q} + A_{q,p-r}) \\    &   \qquad \times (A_{p,q}+A_{q,p} + A_{-p-q,q}+A_{q,-p-q} + A_{p,-p-q}+A_{-p-q,p}) \\    &   \qquad +N^{\kappa -1} \sum _{p,q,r \in \Lambda ^*} \hat{V} (r/N^{1-\kappa }) A_{p,q} \\    &   \qquad \times (A_{q+r,-p-q} + A_{-p-q, q+r} + A_{p-r, -p-q} + A_{-p-q, p-r} + A_{p-r, q+r} + A_{q+r, p-r}). \end{aligned}$$

The last term corresponds to the contribution proportional to $A_{-p-q+r,p-r}$ in the first line, after switching to new variables $-p-q +r \rightarrow p, p-r \rightarrow q$. Rewriting

$$\begin{aligned}\begin{aligned} \sum _{p,q,r} \hat{V} (r/N^{1-\kappa })&A_{p.q} (A_{q+r,-p-q} + A_{-p-q, q+r} + A_{p-r,-p-q} + A_{-p-q,p-r}) \\  &= \sum _{p,q,r} \hat{V} (r/N^{1-\kappa }) (A_{p,q} + A_{q,p}) (A_{p-r,-p-q} + A_{-p-q,p-r}) \\  &= \sum _{p,q,r} \hat{V} (r/N^{1-\kappa }) (A_{p-r,q} + A_{q,p-r}) (A_{p,-p-q} + A_{-p-q,p}) \end{aligned} \end{aligned}$$

we conclude that

$$\begin{aligned} \begin{aligned} \langle \xi , \mathcal {V}_N \xi \rangle&= N^{\kappa -1} \sum _{p,q,r \in \Lambda ^*} \hat{V} (r/N^{1-\kappa })(A_{p-r,q} + A_{q,p-r}) \\&\quad \times (A_{p,q}+A_{q,p} + A_{-p-q,q}+A_{q,-p-q} + 2A_{p,-p-q}+2A_{-p-q,p}) \\&\quad +N^{\kappa -1} \sum _{p,q,r \in \Lambda ^*} \hat{V} (r/N^{1-\kappa }) A_{p,q} (A_{p-r, q+r} + A_{q+r, p-r}) + O(N^{4\kappa -1}). \end{aligned} \end{aligned}$$

Next, we observe that

181 $$\begin{aligned}  &   A_{p-r,q} + A_{q,p-r} = N^{-1/2} \eta _{p-r} \sigma _q - N^{-1/2} \frac{2(p-r) \cdot q}{(p-r)^2 + q^2 + (p-r+q)^2} \sigma _q \eta _{p-r} \nonumber \\    &   \quad + N^{-1/2} \frac{2q^2 \eta _q (\sigma _{p-r} - \eta _{p-r})}{(p-r)^2 + q^2 + (p-r+q)^2} + N^{-1/2} \frac{2 q^2 \eta _{p-r} (\eta _q -\sigma _q)}{(p-r)^2 + q^2 + (p-r+q)^2}. \nonumber \\ \end{aligned}$$

Hence, we can decompose

$$\begin{aligned} \langle \xi , \mathcal {V}_N \xi \rangle = \sum _{j=1}^5 C_{\mathcal {V},j} + O(N^{4\kappa -1}) \end{aligned}$$

where

182 $$\begin{aligned} C_{\mathcal {V},1}= &   N^{\kappa -3/2} \sum \hat{V} (r/N^{1-\kappa }) \eta _{p-r} \sigma _q \nonumber \\  &   \times (A_{p,q}+A_{q,p} + A_{-p-q,q}+A_{q,-p-q} + 2A_{p,-p-q}+2A_{-p-q,p}) \nonumber \\ C_{\mathcal {V},2}= &   -N^{\kappa -3/2} \sum \hat{V} (r/N^{1-\kappa }) \frac{2 (p-r) \cdot q}{(p-r)^2 + q^2 + (p-r+q)^2} \eta _{p-r} \sigma _q \nonumber \\  &   \times (A_{p,q}+A_{q,p} + A_{-p-q,q}+A_{q,-p-q} + 2A_{p,-p-q}+2A_{-p-q,p}) \nonumber \\ C_{\mathcal {V},3}= &   N^{\kappa -3/2} \sum \frac{2q^2 (\eta _q - \sigma _q) \eta _{p-r}}{(p-r)^2 + q^2 + (p-r+q)^2} \nonumber \\  &   \times (A_{p,q}+A_{q,p} + A_{-p-q,q}+A_{q,-p-q} + 2A_{p,-p-q}+2A_{-p-q,p}) \nonumber \\ C_{\mathcal {V},4}= &   N^{\kappa -3/2} \sum \frac{2q^2 \eta _q (\sigma _{p-r} - \eta _{p-r})}{(p-r)^2 + q^2 + (p-r+q)^2} \nonumber \\  &   \times (A_{p,q}+A_{q,p} + A_{-p-q,q}+A_{q,-p-q} + 2A_{p,-p-q}+2A_{-p-q,p}) \nonumber \\ C_{\mathcal {V},5}= &   N^{\kappa -1} \sum \hat{V} (r/N^{1-\kappa }) A_{p,q} (A_{p-r,q+r} + A_{q+r,p-r}). \end{aligned}$$

The last three terms are negligible. In fact, with the bounds in Lemma 7, we find

$$\begin{aligned} \begin{aligned} |C_{\mathcal {V},5} |&\le C N^{5\kappa -2} \sum _{\begin{array}{c} p,q,r\in \Lambda ^*:\\ p,q, p+r, q-r\ne 0 \end{array}} |\hat{V}(r/N^{1-\kappa }) ||p |^{-2}|p+r |^{-2}|q |^{-2}|q-r |^{-2}\\&\le C N^{5\kappa -2} \sum _{r\in \Lambda ^*_+} |\hat{V}(r/N^{1-\kappa }) ||r |^{-2} \le C N^{4\kappa -1}. \end{aligned} \end{aligned}$$

Moreover,

$$\begin{aligned} \begin{aligned} |C_{\mathcal {V},4} |&\le C N^{\kappa -2} \Vert \eta -\sigma \Vert _1 (\Vert \eta \Vert _2^2+\Vert \sigma \Vert _2^2)(\Vert \eta \Vert _1+\Vert \sigma \Vert _1) \le C N^{4\kappa -1} \end{aligned} \end{aligned}$$

and

$$\begin{aligned} \begin{aligned} |C_{\mathcal {V}, 3} |&\le C N^{\kappa -2} \Vert \eta /|\cdot |^2 \Vert \Vert |\cdot |^2(\eta -\sigma ) \Vert _2(\Vert \eta \Vert _2+\Vert \sigma \Vert _2)(\Vert \eta \Vert _1+\Vert \sigma \Vert _1) \le C N^{4\kappa -1}. \end{aligned} \end{aligned}$$

Next, we consider the term $C_{\mathcal {V},1}$. From Lemma 3, we haveThis implies thatWith (180), we obtain183Expanding $A_{p,q} + A_{q,p}$ and $A_{-p-q,q} + A_{q,-p-q}$ similarly to (181) and observing that the contribution of all terms containing the difference $\sigma - \eta$ are negligible (similarly as $C_{\mathcal {V},3}, C_{\mathcal {V}_4}$ above), we conclude that

$$\begin{aligned} \begin{aligned} C_1 = \;&- N^{\kappa -1} \sum _{p,q \in \Lambda ^*} \hat{V}(p/N^{1-\kappa }) \sigma _q^2 \Big ( \frac{2\eta _p \, p \cdot q }{p^2 + q^2 + (p+q)^2} - \frac{2\eta _{p+q} (p+q) \cdot q}{p^2 + q^2 + (p+q)^2} \Big ) \\  &+ N^{\kappa -1}\sum _{p,q\in \Lambda ^*} \hat{V}(p/N^{1-\kappa }) \sigma ^2_q (\eta _p + \eta _{p+q}) + O (N^{4\kappa -1}).\end{aligned} \end{aligned}$$

Switching p+q→-p in the second term on the first line and using that $\Vert \eta - \eta _\infty \Vert _1 \le C N^{3\kappa /2}$ in the second line, we conclude that

184 $$\begin{aligned} \begin{aligned} C_1 = \;&- N^{\kappa -1} \sum _{p,q \in \Lambda ^*} \big ( \hat{V}(p/N^{1-\kappa }) + \hat{V} ((p+q)/N^{1-\kappa }) \big ) \frac{2\eta _p \sigma _q^2 \, p \cdot q }{p^2 + q^2 + (p+q)^2} \\  &+ N^{\kappa -1}\sum _{p,q\in \Lambda ^*} \hat{V}(p/N^{1-\kappa }) \sigma ^2_q (\eta _{\infty ,p} + \eta _{\infty , p+q}) + O (N^{4\kappa -1}).\end{aligned} \end{aligned}$$

As for $C_2$, we expand $A_{p,-p-q} + A_{-p-q,p}$ similarly to (181) and we observe that contributions proportional to $\eta -\sigma$ are negligible (similarly as $C_{\mathcal {V},3}, C_{\mathcal {V}_4}$ above) and that the remaining terms can be combined into

$$\begin{aligned} \begin{aligned} -&N^{-1/2} \frac{2p \cdot (-p-q)}{p^2 + q^2 + (p+q)^2} \eta _p \sigma _{p+q} + N^{-1/2} \eta _p \sigma _{p+q} \\  &= 2 N^{-1/2} \frac{p^2 + (p+q)^2}{p^2 + q^2 + (p+q)^2} \eta _p \eta _{p+q} \!+\! 2 N^{-1/2} \frac{p^2 + (p+q)^2}{p^2 + q^2 + (p+q)^2} \eta _p (\sigma _{p+q} \!-\! \eta _{p+q}). \end{aligned} \end{aligned}$$

Since also the contribution of the last term, proportional to $\sigma _{p+q} - \eta _{p+q}$, is negligible, we arrive atAgain, we can replace $\sigma _q$ by $\eta _q$, with a negligible error. Also, using the definition (64) for replacing $2(p+q)^2 \eta _{p+q}$ by  and then removing the momentum restriction on p+q produces contributions of order $N^{4\kappa -1}$. Similarly, we can replace the smooth cutoff contained in η by a sharp one at $N^{\kappa /2}$ noting that the difference is supported on momenta between $N^{\kappa /2}$ and $2N^{\kappa /2}$. We conclude that185Next, we consider the term $C_{\mathcal {V},2}$, from (182). We start by observing that

$$\begin{aligned} \begin{aligned} \Big | N^{\kappa -3/2}&\sum _{p,q,r} \hat{V} (r/N^{1-\kappa }) \frac{2 (p-r) \cdot q}{(p-r)^2 + q^2 + (p-r+q)^2} \eta _{p-r} \sigma _q (A_{p, -p-q} + A_{-p-q,p}) \Big | \\  &\le C N^{\kappa -3/2} \sum _{p,q,r} \frac{|r||q|}{r^2 + q^2 + (r+q)^2} |\eta _{r}| |\sigma _q| (|A_{p,-p-q}| + |A_{-p-q,p}|) \\  &\le C N^{4\kappa -2} \sum _{p,q,r} \frac{|r| |\eta _r|}{q^2 (q+r)^2} \le C N^{4\kappa -1} \end{aligned} \end{aligned}$$

where we applied (71), from Lemma 7. Thus, we have

$$\begin{aligned} \begin{aligned} C_{\mathcal {V},2} = \;&-N^{\kappa -3/2} \sum \hat{V} (r/N^{1-\kappa }) \frac{2 (p-r) \cdot q}{(p-r)^2 + q^2 + (p-r+q)^2} \eta _{p-r} \sigma _q \\  &\hspace{3cm} \times (A_{p,q}+A_{q,p} + A_{-p-q,q}+A_{q,-p-q}) + O(N^{4\kappa -1}) \\ = \;&-N^{\kappa -3/2} \sum \hat{V} ((p-r)/N^{1-\kappa }) \frac{2 p \cdot q}{p^2 + q^2 + (p+q)^2} \eta _{p} \sigma _q \\  &\quad \times (A_{r,q}+A_{q,r} + A_{-r-q,q}+A_{q,-r-q}) + O(N^{4\kappa -1}) \end{aligned} \end{aligned}$$

after switching $p-r \rightarrow p, r \rightarrow p$. We expand $A_{r,q} + A_{q,r}$ and $A_{-r-q,q} + A_{q,-r-q}$ similarly as in (181) and, as usual, we observe that contributions containing the difference $\sigma -\eta$ are negligible. Thus, we arrive at

$$\begin{aligned} \begin{aligned} C_{\mathcal {V},2}&= -N^{\kappa -2} \sum (\hat{V} ((p-r)/N^{1-\kappa }) \\&\quad + \hat{V} ((p+q-r)/N^{1-\kappa }) ) \frac{2 p \cdot q}{p^2 + q^2 + (p+q)^2} \eta _{p} \eta _{r} \sigma ^2_q \\  &\quad + N^{\kappa -2} \sum \big ( \hat{V} ((p-r)/N^{1-\kappa }) + \hat{V} ((p+q-r)/N^{1-\kappa }) \big ) \\  &\quad \times \frac{2 p \cdot q}{p^2 + q^2 + (p+q)^2} \frac{2r \cdot q}{r^2 + q^2 + (r+q)^2} \eta _{p} \eta _r \sigma ^2_q + O(N^{4\kappa -1}). \end{aligned} \end{aligned}$$

Let us consider the second term, for example the contribution proportional to $\hat{V} ((p-r)/N^{1-\kappa })$ (the contribution proportional to $\hat{V} ((p+q-r)/N^{1-\kappa })$ can be treated analogously). With the change of variable r→-r, we can write

$$\begin{aligned} \begin{aligned} N^{\kappa -2}&\sum \hat{V} ((p-r)/N^{1-\kappa }) \frac{2 p \cdot q}{p^2 + q^2 + (p+q)^2} \frac{2r \cdot q}{r^2 + q^2 + (r+q)^2} \eta _{p} \eta _r \sigma ^2_q \\ = \;&\frac{N^{\kappa -2}}{2} \sum \big ( \hat{V} ((p-r)/N^{1-\kappa }) - \hat{V} ((p+r)/N^{1-\kappa }) \big ) \\  &\quad \times \frac{2 p \cdot q}{p^2 + q^2 + (p+q)^2} \frac{2r \cdot q}{r^2 + q^2 + (r+q)^2} \eta _{p} \eta _r \sigma ^2_q \\  &-N^{\kappa -2} \sum \hat{V} ((p+r)/N^{1-\kappa }) \frac{2 p \cdot q}{p^2 + q^2 + (p+q)^2} \\  &\quad \times \frac{(2 r \cdot q)^2}{\big (r^2 + q^2 + (r+q)^2\big ) \big ( r^2 + q^2 + (r-q)^2 \big )} \eta _{p} \eta _r \sigma ^2_q.\end{aligned} \end{aligned}$$

In the first term, we bound $|\hat{V}((p-r)/N^{1-\kappa })-\hat{V}((p+r)/N^{1-\kappa })|\le C |r| / N^{1-\kappa }$ and, using $\Vert \eta \Vert _1 \le CN$,

$$\begin{aligned} \begin{aligned} N^{2\kappa -3}&\sum _{p,q,r} \frac{|p| |r|^2 |q|^2 |\eta _p| |\eta _r| |\sigma _q|^2}{(p^2 + q^2 + (p+q)^2) (r^2 + q^2 + (r+q)^2)} \\&\quad \le CN^{2\kappa -2} \sum _{p,q} \frac{|p| |q|^2 |\eta _p| |\sigma _q|^2}{(p^2 + q^2 + (p+q)^2)} \le CN^{4\kappa -2} \sum _{p,q} \frac{|p| |\eta _p|}{(p+q)^2 q^2} \le C N^{4\kappa -1}. \end{aligned} \end{aligned}$$

In the second term we proceed similarly, using $|r|^2 |\eta _r| \le C N^{\kappa }$ and $\sum _r |r|^{-2} |r+q|^2 \le C |q|^{-1}$. We conclude that

$$\begin{aligned} N^{\kappa -2} \sum \hat{V} ((p-r)/N^{1-\kappa }) \frac{2 p \cdot q}{p^2 + q^2 + (p+q)^2} \frac{2r \cdot q}{r^2 + q^2 + (r+q)^2} \eta _{p} \eta _r \sigma ^2_q \!=\! O (N^{4\kappa -1}) \end{aligned}$$

and thus thatCombined with (183), (184), (185), we obtain186We can replace $\eta _p$ by $\eta _{\infty ,p}\mathbb {1}(|\cdot |\ge N^{\kappa /2})$, because

$$\begin{aligned} N^{\kappa -1} \sum _{|p| < 2N^{\kappa /2}, q\in \Lambda ^*} \frac{|p| |q| |\eta _{\infty ,p}| |\sigma _q|^2}{p^2 + q^2 + (p+q)^2} \le N^{5\kappa /2 - 1} \sum _{p,q \in \Lambda ^*} \frac{|q| |\sigma _q|^2}{p^2 (p+q)^2} \le C N^{4\kappa -1}. \end{aligned}$$

Similarly, we can also replace $\sigma _q^2$ by $\eta _{\infty ,q}^2\mathbb {1}(|\cdot |\ge N^{\kappa /2})$. This follows from a symmetrization using a change of variable q→-q, because

$$\begin{aligned} \begin{aligned} N^{\kappa -1}&\sum _{p,q\in \Lambda ^*:|p |\ge N^{\kappa /2}} \frac{|p\cdot q|^2 |\eta _{\infty ,p}| |\sigma _q^2-\eta _{\infty ,q}^2\mathbb {1}(|\cdot |\ge N^{\kappa /2}) |}{(p^2 + q^2 + (p+q)^2)(p^2 + q^2 + (p-q)^2)} \\&\le N^{2\kappa -1} \sum _{p,q\in \Lambda ^*:|p |\ge N^{\kappa /2}} \frac{|q|^2 |\sigma _q^2-\eta _{\infty ,q}^2\mathbb {1}(|\cdot |\ge N^{\kappa /2}) |}{p^4}\\&\le N^{5\kappa /2 -1} \sum _{q\in \Lambda ^*} |\sigma _q-\eta _{\infty ,q}\mathbb {1}(|\cdot |\ge N^{\kappa /2}) | \le C N^{4\kappa -1} \end{aligned} \end{aligned}$$

and

$$\begin{aligned} \begin{aligned}&N^{2\kappa -2} \sum _{\begin{array}{c} p,q\in \Lambda ^*:|p |\ge N^{\kappa /2} \end{array}} \frac{|p| |q|^2 |\eta _{\infty ,p}| |\sigma _q^2-\eta _{\infty ,q}^2\mathbb {1}(|\cdot |\ge N^{\kappa /2}) |}{p^2 + q^2 + (p+q)^2}\\&\quad \le N^{3\kappa -2} \sum _{\begin{array}{c} p,q\in \Lambda ^*:|p |\ge N^{\kappa /2} \end{array}} \frac{ |\eta _{\infty ,p}| |\sigma _q-\eta _{\infty ,q}\mathbb {1}(|\cdot |\ge N^{\kappa /2}) |}{|p |} \le C \log (N) N^{4\kappa -1-\beta /2}. \end{aligned} \end{aligned}$$

We can therefore rewrite the last term on the r.h.s. of (186) asconcluding the proof of the lemma. □

We are now ready to show Theorem 2.

#### Proof of Theorem 2

Combining Lemma 13 with Lemmas 15, 18, 19, 20 and 27, we obtain, for any $\kappa \ge 1/2$,187Let us consider the term $S_1$ first. Since the summand decays as $N^{3\kappa } |p|^{-4}$, as $|p| \rightarrow \infty$, we can restrict the sum to $|p| \le N^{1-\kappa }$, up to a negligible error. We defineThen

$$\begin{aligned} \begin{aligned} S_1 =&\; \frac{1}{2}\sum _{\begin{array}{c} p\in \Lambda ^*_+\\ |p |\le N^{1-\kappa } \end{array}} \left( \sqrt{|p|^4+16\pi \mathfrak {a} N^\kappa p^2} - p^2 - 8\pi \mathfrak {a}N^\kappa + \frac{N^{2\kappa }(8\pi \mathfrak {a})^2}{2|p |^2} \right) \\&+ \frac{1}{2}\int _0^1 ds\int _0^s dt \sum _{\begin{array}{c} p\in \Lambda ^*_+\\ |p |\le N^{1-\kappa } \end{array}} \frac{d^2}{dt^2} g_p(t) +O(N^{4\kappa -1}) \end{aligned} \end{aligned}$$

where we used  and $g'_p (0) = 0$, because ∫xV(x)f(x)dx=0 by symmetry. Explicit computation of the derivatives of $g_p$ leads to the estimate

$$\begin{aligned} |g_p''(t) | \le \left\{ \begin{array}{ll} C N^{4\kappa -2} & \text { if } |p | \le K N^{\kappa /2}\\ C N^{4\kappa -2} \frac{N^{\kappa }}{|p |^2} & \text { if } |p | > K N^{\kappa /2} \end{array} \right. \end{aligned}$$

if K>0 is large enough. Hence,

$$\begin{aligned} \begin{aligned} S_1&= \frac{1}{2}\sum _{\begin{array}{c} p\in \Lambda ^*_+ \end{array}} \left( \sqrt{p^4+16\pi \mathfrak {a} N^\kappa p^2} - p^2 - 8\pi \mathfrak {a}N^\kappa + \frac{N^{2\kappa }(8\pi \mathfrak {a})^2}{2|p |^2} \right) +O(N^{4\kappa -1}).\end{aligned} \end{aligned}$$

Defining

$$\begin{aligned} h(p) = \sqrt{p^4+16\pi \mathfrak {a} N^\kappa p^2} - p^2 - 8\pi \mathfrak {a}N^\kappa + \frac{N^{2\kappa }(8\pi \mathfrak {a})^2}{2|p |^2} \end{aligned}$$

we can write

$$\begin{aligned} \begin{aligned} S_1 =\;&\frac{1}{2(2\pi )^3}\int dp^3 \left( \sqrt{p^4+16\pi \mathfrak {a} N^\kappa p^2} - p^2 - 8\pi \mathfrak {a}N^\kappa + \frac{N^{2\kappa }(8\pi \mathfrak {a})^2}{2|p |^2} \right) \\&+ \frac{1}{2(2\pi )^3}\sum _{\begin{array}{c} p\in \Lambda ^*_+ \end{array}} \int _{[-\pi ,\pi ]^3} d\zeta \, (h(p)-h(p+\zeta )) +O(N^{2\kappa })+O(N^{4\kappa -1})\\ \end{aligned} \end{aligned}$$

where the error $O (N^{2\kappa })$ arises from the integral around p=0. By explicit computation, we find

$$\begin{aligned} \begin{aligned} \Big | \frac{d^2}{ds^2} h(p+s\zeta ) \Big | \le C \frac{N^{2\kappa }}{|p+s\zeta |^4}. \end{aligned} \end{aligned}$$

Thus,

$$\begin{aligned} \begin{aligned} \sum _{\begin{array}{c} p\in \Lambda ^*_+ \end{array}} \int _{[-\pi ,\pi ]^3} d\zeta \, (h(p)-h(p+\zeta ))&= - \sum _{\begin{array}{c} p\in \Lambda ^*_+ \end{array}} \int _{[-\pi ,\pi ]^3} d\zeta \int _0^1 ds \, \frac{d}{ds} h(p+s\zeta )\\&= - \sum _{\begin{array}{c} p\in \Lambda ^*_+ \end{array}} \int _{[-\pi ,\pi ]^3} d\zeta \int _0^1 ds \int _0^s \, \frac{d^2}{dt^2} h(p+t\zeta )\\&=O(N^{2\kappa }) \end{aligned} \end{aligned}$$

because (d/ds)h(p+sζ), evaluated at s=0, is an odd function of *p* (and therefore its sum over *p* vanishes). We conclude that, for $\kappa \ge 1/2$,

$$\begin{aligned} \begin{aligned} S_1&= \frac{1}{2(2\pi )^3}\int dp^3 \left( \sqrt{p^4+16\pi \mathfrak {a} N^\kappa p^2} - p^2 - 8\pi \mathfrak {a}N^\kappa + \frac{N^{2\kappa }(8\pi \mathfrak {a})^2}{2|p |^2} \right) +O(N^{4\kappa -1})\\&= \frac{(8\pi \mathfrak {a}N^\kappa )^{5/2}}{2(2\pi )^3}\int dp^3 \left( \sqrt{p^4+2 p^2} - p^2 - 1 + \frac{1}{2|p |^2} \right) +O(N^{4\kappa -1})\\&= \frac{512\sqrt{\pi }}{15}\mathfrak {a}^{5/2}N^{5\kappa /2} +O(N^{4\kappa -1}). \end{aligned} \end{aligned}$$

Next, we consider the term $S_2$, as defined in (187). Sincewe can restrict the sum to $|p|, |q| \le N^{1-\kappa }$. Estimating , we obtain

$$\begin{aligned} S_2 = - 2048 N^{4\kappa -1} \pi ^4 \mathfrak {a}^4 \sum _{\begin{array}{c} p,q\in \Lambda ^*\\ N^{\kappa /2}\le |p |,|q |\le N^{1-\kappa } \end{array}} \frac{1}{p^2q^2(p^2+q^2+(p+q)^2)} +O(N^{4\kappa -1}). \end{aligned}$$

As for the term $S_3$, we can proceed as we did for $S_2$ to restrict the sum to $|q| \le N^{1-\kappa }$. In order to restrict it to $|p| \le N^{1-\kappa }$, on the other hand, we need additionally a symmetrization argument (replacing *q* with -q). This argument is very similar to [10, Eq. (2.39)]; we skip the details. After restricting to $|p|,|q| \le N^{1-\kappa }$, we can use  as we did for $S_2$, to conclude that

$$\begin{aligned} S_3 = 2048 N^{4\kappa -1} \pi ^4 \mathfrak {a}^4 \sum _{\begin{array}{c} p,q\in \Lambda ^*\\ N^{\kappa /2}\le |p |, |q |\le N^{1-\kappa } \end{array}} \frac{p\cdot q}{q^4 p^2(p^2+q^2+(p+q)^2)} +O(N^{4\kappa -1}). \end{aligned}$$

We arrive at

$$\begin{aligned} S_2 + S_3 = 2048 \,N^{4\kappa -1} \pi ^4 \mathfrak {a}^4 \sum _{\begin{array}{c} p,q\in \Lambda ^*\\ N^{\kappa /2}\le |p |, |q |\le N^{1-\kappa } \end{array}} \frac{p\cdot q - q^2}{q^4 p^2(p^2+q^2+(p+q)^2)} +O(N^{4\kappa -1}). \end{aligned}$$

Proceeding exactly as in [10, Section 2], we can replace the sum by an integral, with only a negligible error. We find

$$\begin{aligned} \begin{aligned} S_2 + S_3 = \;&\frac{16 \mathfrak {a}^4}{\pi ^2} \, N^{4\kappa -1} \int _{\begin{array}{c} N^{\kappa /2}\le |p |,|q |\le N^{1-\kappa } \end{array}} dpdq \frac{p\cdot q -q^2}{p^2q^4(p^2+q^2+p\cdot q)} \\  &+ O(N^{2\kappa -\beta /2}) + O(N^{4\kappa -1}). \end{aligned} \end{aligned}$$

Rescaling the integration variables, $p,q \rightarrow p/N^{\kappa /2}, q/N^{\kappa /2}$, we obtain

$$\begin{aligned} \begin{aligned} S_2 +S_3 = \frac{16 \mathfrak {a}^4}{\pi ^2} \, N^{4\kappa -1} \int _{\begin{array}{c} 1 \le |p |,|q |\le N^{\beta /2} \end{array}} dpdq \frac{p\cdot q -q^2}{p^2q^4(p^2+q^2+p\cdot q)} + O(N^{4\kappa -1}). \end{aligned} \end{aligned}$$

for $\kappa \ge 1/2$. Up to an error of order $N^{4\kappa -1}$, we can remove the restrictions on *p*. By explicit computation, we conclude that

$$\begin{aligned}\begin{aligned} S_2 + S_3&= \frac{16 \mathfrak {a}^4}{\pi ^2} \, N^{4\kappa -1} \int _{\begin{array}{c} 1 \le |q |\le N^{\beta /2} \end{array}} dpdq \frac{p\cdot q -q^2}{p^2q^4(p^2+q^2+p\cdot q)} +O(N^{4\kappa -1}) \\  &= -32\pi \left( \frac{4\pi }{3}-\sqrt{3}\right) \mathfrak {a}^4 N^{4\kappa -1} \log (N^{\beta }) +O(N^{4\kappa -1} ) \,. \end{aligned} \end{aligned}$$

□

## Data Availability

This manuscript has no associated data.
